# Halide-Free Continuous Synthesis of Hydrophobic Ionic Liquids

**DOI:** 10.1021/acssuschemeng.2c02871

**Published:** 2022-08-16

**Authors:** Kristof Stagel, Andrea Szpecht, Dawid Zielinski, Marcin Smiglak, Michael Schnürch, Katharina Bica-Schröder

**Affiliations:** † Institute of Applied Synthetic Chemistry, TU Wien, Getreidemarkt 9/163, Vienna 1060, Austria; ‡ Poznan Science and Technology Park, Adam Mickiewicz University Foundation, St. Rubiez 46, Poznan 61-612, Poland; § Faculty of Chemistry, Adam Mickiewicz University in Poznan, St. Uniwersytetu Poznanskiego 8, Poznan 61-614, Poland

**Keywords:** alkyl bistriflimide, triflic anhydride, ionic liquid, bis(trifluoromethanesulfonyl)imide, solvent-free

## Abstract

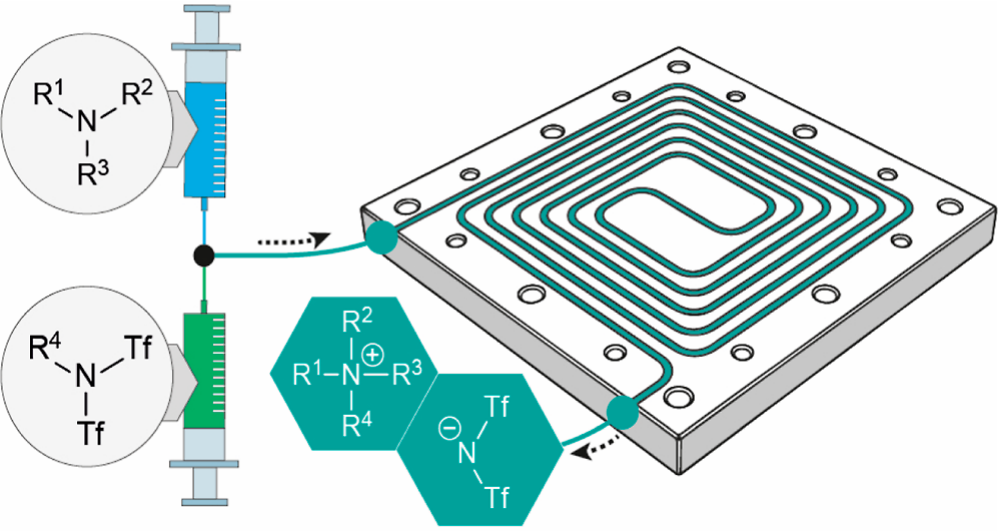

Herein, we present a novel approach for the halide-free, continuous-flow preparation of hydrophobic ionic liquids (ILs) relying on the bis(trifluoromethanesulfonyl)imide (bistriflimide, NTf_2_^–^) anion. The simple yet fast two-step synthetic route, which involves the formation of different alkyl bistriflimides (R^4^NTf_2_), followed by the quaternization with an amine nucleophile, led to the desired ILs in high yields and excellent purities without any byproduct formation. The variable alkyl chain (R^4^) length and the broad range of the applicable nucleophiles (R^1^R^2^R^3^N) offer considerable flexibility to the synthetic protocol. The quaternization can be performed under solvent-free conditions; moreover, the homogeneous nature of these reactions allows the application of modern continuous-flow technologies. Given these advantages, the methodology can afford not just a fast and efficient alternative for the conventional synthesis of such compounds with reduced waste water production but their negligible halide content might provide a significantly broader application range of the IL products, especially for the field of materials science.

## Abstract 2

A halide- and solvent-free method is applied for the continuous-flow production of NTf_2_^−^-based ionic liquids.

## Introduction

Within the past decades, the application of ionic liquids (ILs) has spread into various fields, including analytics,^1^ catalysis, extractions, various electrochemical applications,^2−6^ and lubrication technologies.^7^ While some applications do not call for high purity, others are extremely sensitive with regard to their metal and halide content, which can cause several problems such as corrosion when they are used as lubricants^8^ or catalyst poisoning in organic synthesis.^9−12^

Until today, the preparation of hydrophobic ILs has mostly relied on a two-step process; the initial quaternization of a nucleophile with an alkyl halide is followed by anion metathesis. Although this state-of-the-art strategy has been described in detail and is well established,^13^ several challenges still exist for the reliable production, and large discrepancies observed in the reported properties of ILs can be traced to metal and halide residuals of the ILs.^14,15^

The initial formation of the organic halide precursor can be challenging as the use of low-volatile alkyl halides as alkylating agents, for example, chloroalkanes in the first step, is time-consuming and might lead to preparative issues on both laboratory and industrial scale. Similarly, the anion metathesis step is problematic due to the onerous removal of metal and halide residues that decidedly influence the physical and chemical behavior of the ILs. Additionally, the stoichiometric formation of an alkali salt, for example, LiCl, as a byproduct is yet another issue in the light of sustainability as exhaustive extractive work-up is required to suppress the residual halide content to a minimum.^16^

These problems with ion metathesis and residual metal and/or halide content can be circumvented in the synthetic route that relies on dimethyl carbonate as a cheap methylating agent.^17^ The method is based on the formation of a methyl carbonate intermediate which is further reacted with equimolar amounts of acid to form the IL upon release of CO_2_ and methanol.^18^ Although this methodology provides access to a broad range of hydrophilic ILs, the complete neutralization of the methyl carbonate salts can cause contamination with excess reagents.

In 2002, Holbrey et al.^19^ reported a method that eliminates the anion metathesis step; direct alkylation of imidazole derivatives with the corresponding dialkyl sulfate led to the formation of the desired IL in one step. A similar strategy has been reported by Kuhlmann and co-workers^20^ for the preparation of imidazolium dialkyl phosphates. The obtained methyl sulfate- or dimethyl phosphate-based ILs can serve as a precursor for further modifications, for example, acid-catalyzed transesterification toward the more hydrophobic esters. The high reactivity of the dialkyl sulfates drastically reduces the time and energy demand of the alkylation step; however, these benefits are outweighed by their enormous toxicity as is the case of dimethyl sulfate. Kim et al.^21^ have developed a synthetic method for halide-free ILs using orthoesters in the presence of an acid. Even though they obtained outstanding yields and a broad range of ILs could be produced with this method, the relatively long reaction time required and the formed byproducts are unfavorable from an environmentally benign perspective.

Although strategies already exist for the halide-free preparation of ILs, unfortunately, these methods have certain limitations. Some of them require significantly toxic reactants, while others are hampered by the complicated removal of contaminants associated with excess reagents. There is clearly a need for an entirely halide-free preparation method for hydrophobic ILs that eliminates either the necessity of anion metathesis with its exhaustive extraction steps or the formation of byproducts in the final step.

**Scheme 1:**
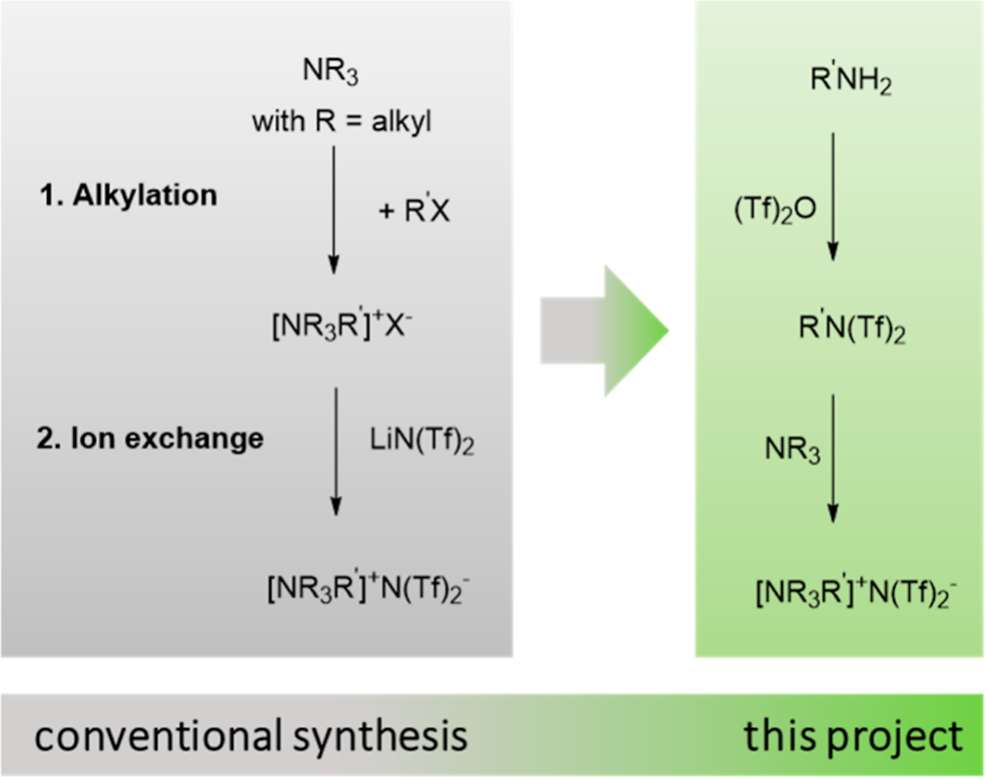
Toward the Halide-Free Synthesis of Hydrophobic ILs

The present research focuses on the development of a halide-free approach for the synthesis of bistriflimide-based hydrophobic ILs *via* formation of alkyl bistriflimides, thus circumventing the problems associated with the state of the art (Scheme 1). The strategy we present offers a benign synthetic route and considerable flexibility in the design of sulfonamide-based ILs, starting from cheap and easily accessible nucleophile precursors. The formation of undesirable byproducts is effectively eliminated, while the application of the continuous-flow method significantly decreases the reaction time, providing a rapid and efficient synthesis of hydrophobic ILs.

## Results and Discussion

**Scheme 2:**
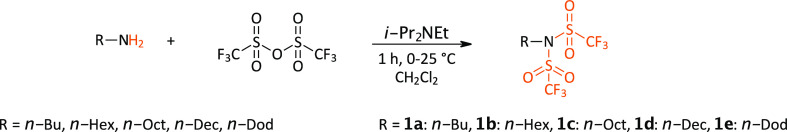
Synthesis of Alkyl Bistriflimides as Precursors for IL Synthesis

Initially, we focused on the optimization of the first step of our synthetic route toward the alkylating agents, that is, the formation of various alkyl bistriflimides in a batchwise fashion. The general strategy^22^ involves the reaction of a primary amine with trifluoromethanesulfonic anhydride (triflic anhydride, Tf_2_O) in the presence of a base. As a primary amine as the substrate, butylamine was selected for the optimization of the reaction parameters. Subsequently, the optimized conditions were applied to the synthesis of a range of alkyl bistriflimides with various alkyl chain lengths (C_4_–C_12_, Scheme 2) in order to introduce a broader diversity with regard to the quaternization step. Short-chained amines were not considered in the preparation process due to their gaseous nature. Since ILs bearing an alkyl chain with odd carbon numbers are less frequently mentioned in the literature, we included only even-numbered alkyl groups in this study. However, based on the experimental work, similar results in terms of yield and purity can be expected with odd-numbered alkyl bistriflimides.

Based on our previous experiments,^23^ we used *N*,*N*-diisopropylethylamine as an acid scavenger and dichloromethane as a solvent.^24^^a^ Increasing the amount of the reagent by 0.05 equiv (2.00–2.05 equiv) had a significant influence on the reaction rate; 97% conversion was observed after 1 h, while 3 h were required to obtain the same conversion using 2.00 equiv of triflic anhydride. A further increase in the amount of Tf_2_O did not significantly favor the reaction rate (see Figure S3, Supporting Information); therefore, a reagent excess of 2.05 equiv was chosen as optimum. It is important to realize that these components—in comparison to the final IL—can be readily purified via distillation, a key advantage compared to the conventional synthesis of bistriflimide-based ILs that only allow for extractive purification. Consequently, after vacuum distillation, the alkyl bistriflimides could be obtained in high purities (see Figures S7–S21, Supporting Information) and with excellent yields (Table 1).

**Table 1:**
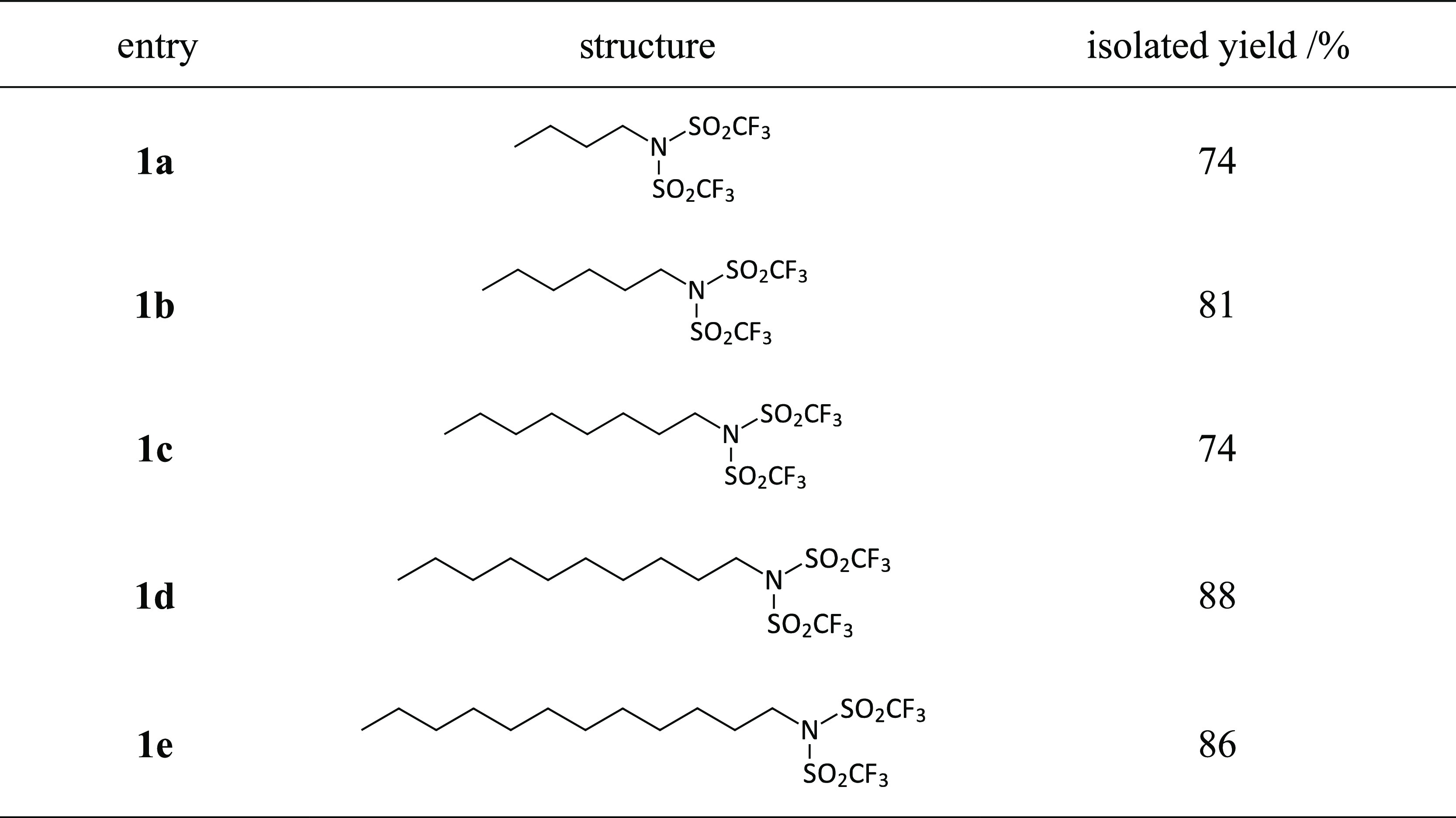
Batchwise Synthesis of Alkyl Bistriflimides as Precursors for the Halide-Free Preparation of NTf_2_^–^-Based ILs^a^

a Performed with 40.00 mmol (1.00 equiv) primary amine, 82 mmol (2.05 equiv) *i*-Pr_2_NEt, and 82.00 mmol (2.05 equiv) trifluoromethanesulfonic anhydride in 110 ml CH_2_Cl_2_. Reaction time: 1 h. After the addition of the reactant dropwise, the temperature was slowly increased from 0 °C to RT. Products were obtained as slightly yellowish liquids after vacuum distillation.

Following the parameter optimization for the synthesis of these alkyl bistriflimide IL precursors, the quaternization step was investigated in detail. All alkylations were first carried out in the batchwise mode, under solvent-free conditions, in glass screw cap vials.

**Scheme 3:**
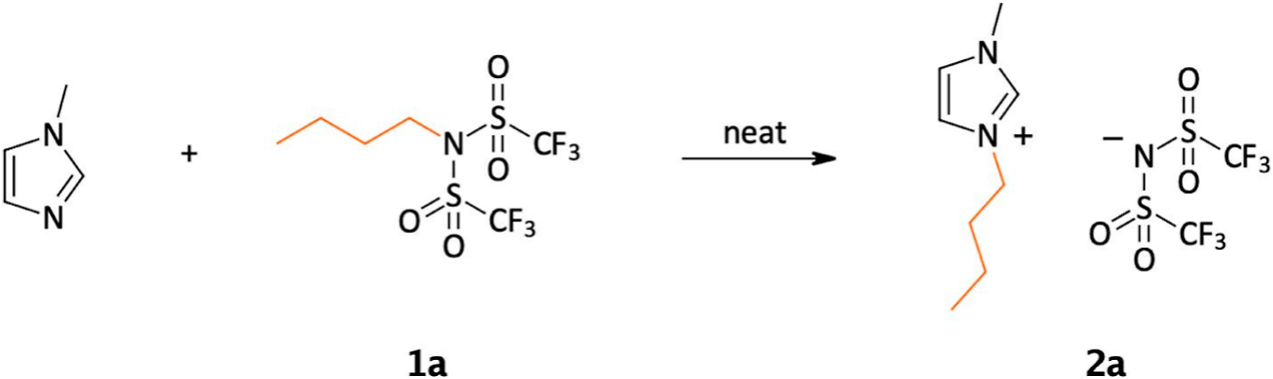
Halide-Free Synthesis of 1-Butyl-3-methyl-imidazolium Bistriflimide (**2a**)

As a model reaction, the synthesis of 1-butyl-3-methylimidazolium bis(trifluoromethylsulfonyl)imide was selected and studied in detail (Scheme 3).

**Figure 1:**
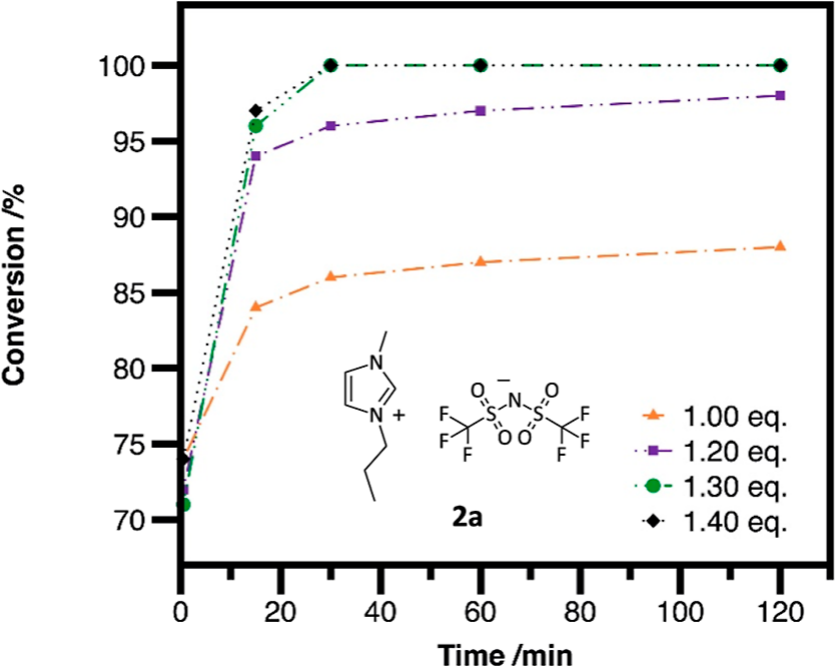
Effect of the alkylating agent equivalents on the reaction rate.

Initially, we investigated the effect of the alkyl bistriflimide’s ratio to the nucleophile on the reaction (Figure 1) at 80 °C. None of the ILs yielded full conversion after 24 h when conventional heating was applied; only approximately 90% conversion could be observed, supposedly due to the volatile nature of the alkyl bistriflimides. An increase in the amount of alkyl bistriflimide from 1.00 to 1.30 equiv exerted a pronounced positive effect on the reaction rate, but any further increase of the reactant beyond this did not significantly accelerate the reaction further. Using 1.30 equiv of the alkylating agent, full conversion could be reached within 30 min.

**Figure 2:**
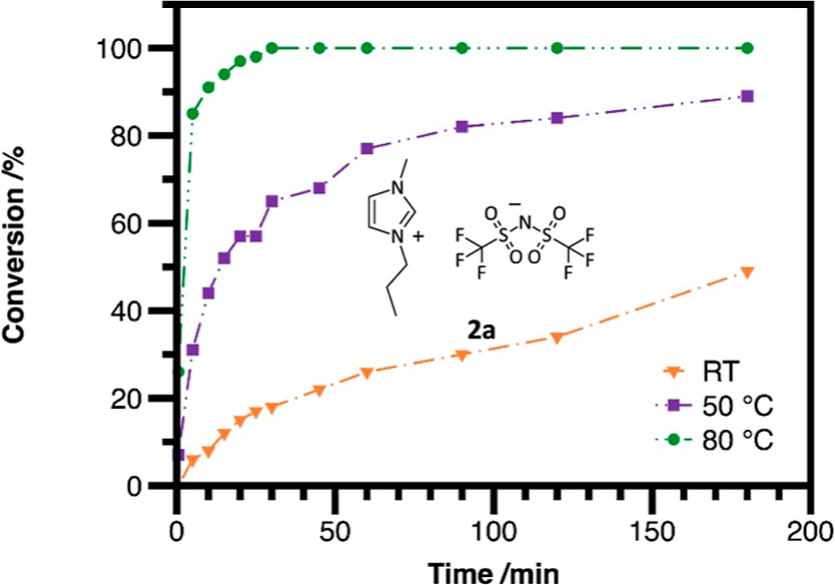
Reaction rate at different temperatures using 1.30 equiv of butyl bistriflimide for the synthesis of **2a**.

To determine the optimal temperature for the quaternization, experiments were carried out at room temperature, 50 and 80 °C. In order to see a more pronounced difference between the results, an increased butyl bistriflimide loading of 1.30 equiv was applied for this optimization stage. The conversion was monitored by ^1^H NMR spectroscopy over time; the first sample was taken 30 s after the addition of the reagent, and subsequently, samples were collected at 5 min intervals until the 30th minute and then after 45, 60, 90, 120, and 180 min.^b^ As expected, the reaction was significantly faster at 80 °C than at 50 °C; after 30 min, full conversion was observed at 80 °C, whereas at 50 °C, 3 h were not sufficient to obtain full conversion (Figure 2, green vs purple). At room temperature, only 49% conversion was observed after 3 h (Figure 2, orange).

The reactivity of the selected alkylating agents was additionally compared, using 1-methylimidazole as a nucleophile, at 80 °C. Since the reaction proceeds fast when 1.30 equiv of the alkylating agent is used, in order to uncover the difference between the reactivities, a 1:1 molar ratio was maintained. No significant difference was observed between the reactivities of butyl bistriflimide (**1a**) and dodecyl bistriflimide (**1e**): in the case of the shorter alkyl bistriflimide, 91% conversion was obtained after 24 h, while 82% conversion could be reached using dodecyl bistriflimide. Presumably, the reason for that is that in every case, a primary carbon atom is attacked by the nucleophile, which has a similar steric hindrance in every alkyl bistriflimide. On the other hand, in the case of dodecyl bistriflimide, the moderate hindering influence of the C_11_ chain connected to the primary carbon atom eventually still exerts a minor influence on the reactivity.

Having optimized the alkyl bistriflimide loading and temperature, we sought to screen different nucleophiles. We focused on ILs that are derived from N-nucleophiles, such as 1-methylimidazole, 1-vinylimidazole, and pyridine, all of which are commercially available at low costs. The vinyl derivatives are of particular interest for the formation of polymeric ILs as conductive polymers; thus, a halide-free protocol is of particular importance.^25−27^

**Scheme 4:**
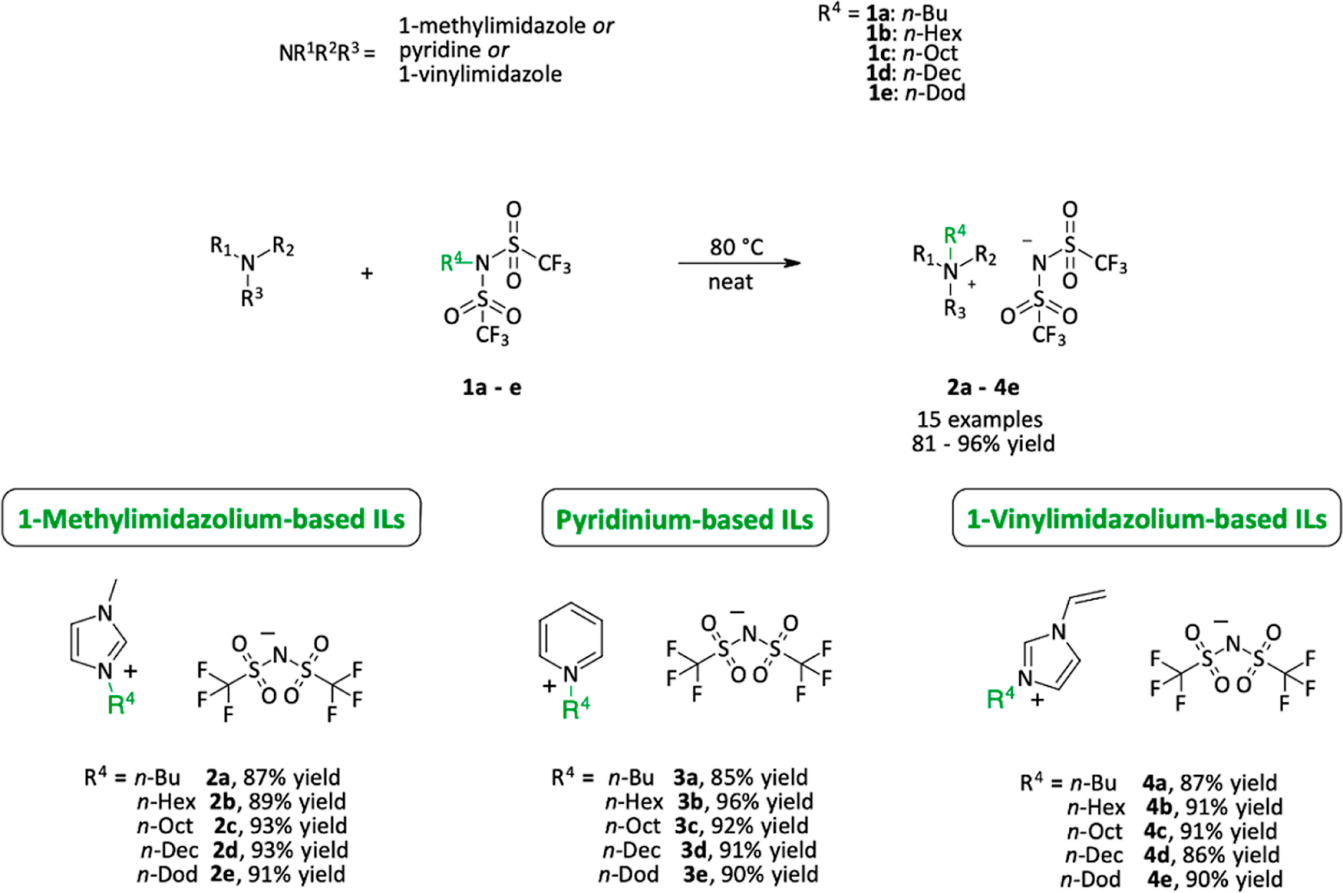
Scope for the Halide-Free Synthesis of Various NTf_2_^–^-Based ILs; Yields Reported Corresponding to Isolated Yields

Gratifyingly, the procedure was found to be quite versatile, as 15 different ILs (**2a**–**4e**) could be prepared in excellent yield and purity (Scheme 4); furthermore, no undesired polymerization was observed during the alkylation of vinylimidazole-derived species.

**Scheme 5:**
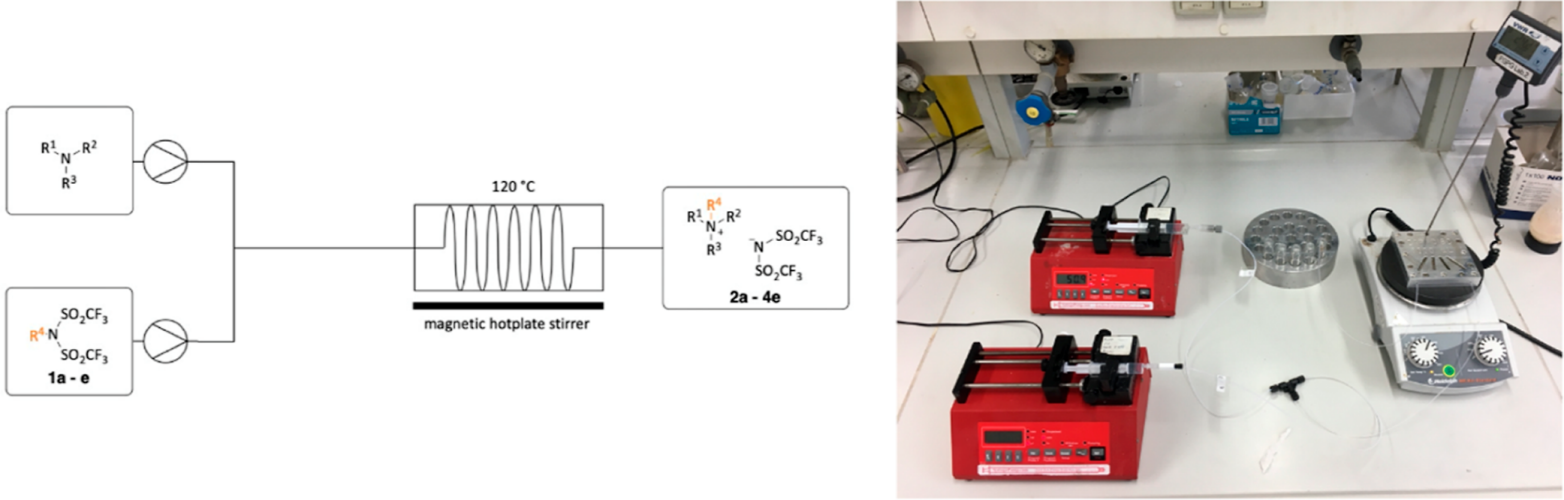
Scheme and Setup of the Continuous-Flow Experiments

We further aimed to reduce the excess of the relatively expensive alkyl bistriflimides since using only 1 equiv is favorable not just from an environmental but also from an economic point of view. Apart from losses due to volatility, the low surface-to-volume ratio of the batchwise process might also limit the reactivity.^28,29^ We therefore investigated the IL synthesis under continuous-flow conditions by using a custom-made microreactor, whose setup is presented in Scheme 5.

**Scheme 6:**
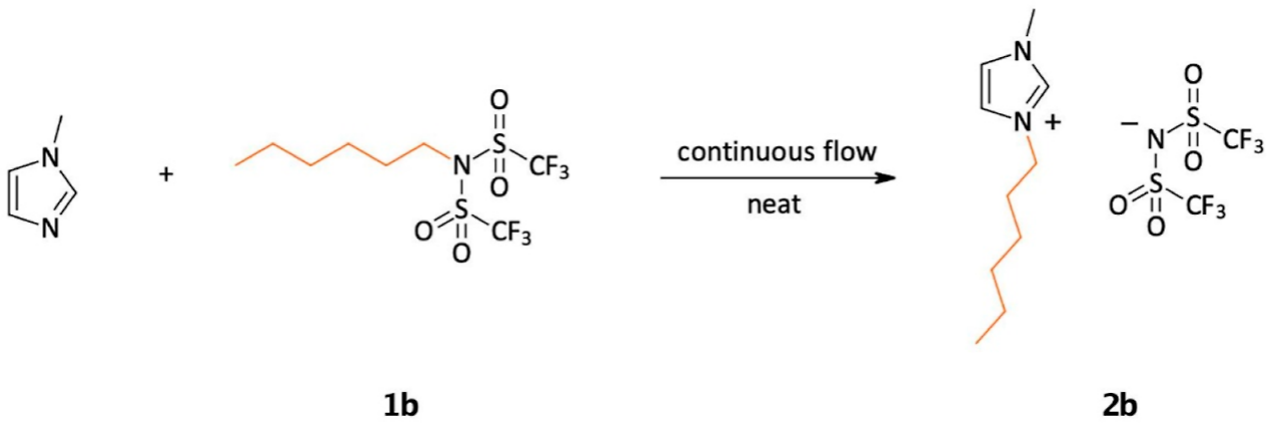
Model Reaction for the Continuous-Flow Experiments

As a model reaction, we examined the synthesis of 1-hexyl-3-methylimidazolium bis(trifluoromethylsulfonyl)imide (Scheme 6); in these experiments, an equimolar ratio of the nucleophile and the alkylating agent was maintained.

Initially, the influence of the temperature on the reaction was investigated. The flow rates were calculated to maintain a residence time of 15 min in order to provide sufficient mixing. At 80 °C, 79% conversion was observed (entry 1, Table 2), which increased to 90% at 100 °C (entry 2, Table 2), and full conversion could be earned when the temperature was set to 120 °C (entry 3, Table 2). Thereafter, the residence time was varied by increasing the flow rate of the reactants. By doubling the flow rate of the reactants, still full conversion could be observed at 120 °C (entry 6, Table 2), which means that the potentially negative impact of the decreased residence time was outweighed by the better mixing achieved at the increased flow rate. Nevertheless, by a further decrease of the residence time to 5 min, this is not the case anymore: only 92% conversion was observed (entry 7, Table 2).

**Table 2:** Parameter Optimization of the Continuous-Flow Experiments for the Synthesis of IL **2b**^a^

		flow rate/μL min^–1^			
entry	residence time/min	substrate	reagent (**1b**)	temperature/°C	conversion^b^/%
1	15	16	51	80	79
2	15	16	51	100	90
**3**	**15**	**16**	**51**	**120**	**100**
4	15	16	51	140	100
5	7.5	32	102	100	89
6	7.5	32	102	120	100
7	5	47	153	120	92

a Reactions were carried out in a 1000 μL reactor fitted with a PTFE capillary tube, and the reagents were supplied with the aid of one–one syringe pumps, respectively. Performed with 7.00 mmol 1-methylimidazole and 7.00 mmol hexyl bistriflimide under solvent-free conditions.

b The conversion was monitored by ^1^H NMR spectroscopy from the crude products. No byproduct formation was observed.

As it is shown in Figure S4 in the Supporting Information, 7.5 min at 120 °C in the continuous mode yielded full conversion by using only 1.00 equiv from the hexyl bistriflimide. Using the same conditions in the batchwise mode, full conversion was not possible to be reached even when the reaction time was extended to 24 h. This clearly indicates the beneficial effect of the enormous surface-to-volume ratio, which results not just in full conversion but also leads to high productivity. Moreover, while this methodology was developed for the synthesis of bistriflimide-based ILs, the microreactor setup and optimized conditions could be used in many other halide-free synthetic approaches, for example, in an orthoester-based or a trimethyl phosphate-based process.

After the initial optimization of the reaction parameters, the continuous-flow synthesis of all the other RTILs (**2a**–**4e**) was accomplished. As it can be seen in Table 3, all ILs were obtained with very good yields.

**Table 3:** Continuous-Flow Synthesis of ILs in a 1000 μL Microreactor^a^

			flow rate/μL min^–1^				
entry	product	residence time/min	substrate	reagent (**1a–1e**)	temperature/°C	yield/%	productivity/g day^–1^
1	**2a**	7.5	35	102	120	90	238
2	**2b**	7.5	31	102	120	94	241
3	**2c**	7.5	28	105	120	99	239
4	**2d**	7.5	25	108	120	98	222
5	**2e**	7.5	24	110	120	92	211
6	**3a**	7.5	35	98	120	90	236
7	**3b**	7.5	32	102	120	92	230
8	**3c**	7.5	28	105	120	86	205
9	**3d**	7.5	25	108	120	95	215
10	**3e**	7.5	24	109	120	90	203
11	**4a**	7.5	38	95	120	92	243
12	**4b**	7.5	35	99	120	92	232
13	**4c**	7.5	31	103	120	98	235
14	**4d**	7.5	28	106	120	93	212
15	**4e**	7.5	26	107	120	91	208

a Reactions were carried out in a 1000 μL reactor fitted with a PTFE capillary tube, and the reagents were supplied with the aid of one–one syringe pumps, respectively. The unreacted starting materials were removed under high vacuum (0.3 mbar) at 90 °C.

After the NTf_2_^–^-based ILs have been synthesized both batchwise and in the continuous mode, they were characterized by NMR spectroscopy and high-resolution mass spectrometry (HR-MS). Their water content was determined by Karl Fischer titration. All ILs have a water content of less than 400 ppm, and the pyridine-based ILs (**3a**–**3e**) have less than 300 ppm (Table S1, Supporting Information).

Concerning purity, the synthesized ILs have been analyzed by ion chromatography,^30^ and a purity of >99.1% was obtained for all ILs synthesized in a continuous flow (Table S1, Supporting Information).

Compared to the halide content of the conventionally synthesized butyl-methylimidazolium bistriflimide ILs, our alternatively developed alkyl bistriflimide-based, halide-free route shows a significant improvement in this regard. The state-of-the-art method requires multiple extraction steps and >200 mL of water to decrease the chloride content to a value below 200 ppm, which is—apart from a considerable workload—yet again unfavorable from an environmentally benign perspective (Table 4). Ultimately, the option for distillation in the case of alkyl bistriflimides can suppress extractive work-up to a minimum compared to the conventional anion metathesis of Cl^–^ against N(Tf)_2_^–^ is also favorable in terms of waste generation. Based on the *E*-factor calculations (Figures S5 and S6, Supporting Information), this method generates approx. 15 kg less waste pro kg product.

**Table 4:** Halide Content of IL **2a** Produced by Our Method and *via* Anion Metathesis

	purity/%	Cl^–^ content/ppm		
IL	bistriflimide-based method^a^	conventional method^b^	washing step	water required in total/mL
**2a**	>99.9	910	#1	40
		840	#2	80
		790	#3	120
		680	#4	140
		520	#5	180
		360	#6	220
		190	#7	240
		160	#8	280

a IL **2a** synthesized *via* the bistriflimide-based method, no removal of chloride residues via aqueous extraction required.

b IL **2a** produced by reacting the corresponding chloride IL precursor (1.00 equiv) with LiNTf_2_ (1.20 equiv) in water as a solvent. The mixture was stirred for 4 h, IL **2a** was extracted with dichloromethane (3 × 30 mL). Then, the combined organic phases were washed 8 times with 40 mL of distilled water. After each washing step, an aliquot of 2 mL was taken from the organic phase. These samples were concentrated, and their halide content was quantified by ion chromatography.

Concerning energy requirements, the first step of the conventional route involves the alkylation of the desired nucleophile with an alkyl halide at elevated temperature, which can take up to 7 days; therefore, it is unfavorable from an energy efficiency point of view. In comparison, the reaction time for alkyl bistriflimides is 1 h only, without the need of heating but under moderate cooling. Elevated temperature is applied in the second step, but the reaction is carried out in 7.5 min, so the overall process is less energy-consuming. Ultimately, in terms of the financial aspects, the halide-free approach is currently less favorable: assuming that the chemicals are bought from the same provider, and working on the same scale, our method is certainly more expensive as the price of the trifluoromethanesulfonic anhydride drastically increases the costs. However, it should be noted that this estimation refers to high-purity trifluoromethanesulfonic anhydride (>99%) purchased on a small scale and that prices are always subject to the demand. Especially in the case of trifluoromethanesulfonic anhydride, prices vary significantly, and other suppliers, scales, and purities render the cost difference significantly smaller.

## Conclusions

A new synthetic method for the halide-free synthesis of bis(trifluoromethanesulfonyl)imide-based, hydrophobic ILs has been developed. The simple, two-step procedure could give straightforward access to a wide variety of NTf_2_-based ILs with good yields and excellent purities. The quaternization step could be carried out under solvent-free conditions and provided high atom efficiency without additional waste formation. The reported method is intrinsically halide free, which offers many benefits for the development of new IL-based technologies that are sensitive to halide content. The overall process is significantly less time-consuming than most reported halide-free methods, and due to the reactions’ homogeneous nature, it could be successfully performed not just in the batchwise application but also in the continuous-flow operation mode. This provided an even more rapid, safe, and easily scalable synthesis of these ILs, allowing them to be obtained in high yields (86–99%) and productivity, with a residence time of 7.5 min only. The high purity renders this process not just only safe and efficient but eventually also makes the IL products suitable for a significantly broader application range, especially for materials science.

## Experimental Section

### Representative Protocol for the Synthesis of Alkyl Bistriflimides

After transferring 40 mmol (1.00 equiv) of the corresponding amine and *N*,*N*-diisopropylethylamine (82 mmol, 2.05 equiv) to a three-neck round-bottom flask, 90 mL of anhydrous dichloromethane were added under an argon atmosphere. The mixture was stirred and cooled down *via* a NaCl/ice bath. Then, trifluoromethanesulfonic anhydride (82 mmol, 2.05 equiv) was dissolved in 20 mL of anhydrous dichloromethane, and it was added to the mixture dropwise, while the temperature was maintained below 5 °C. Once the triflic anhydride was added, the NaCl/ice bath was removed, and the mixture was stirred at room temperature for 1 h. After that, the mixture was washed with saturated NaHCO_3_ solution, 2 N HCl solution, and distilled water. The aqueous phases were back-extracted with dichloromethane. The combined organic layers were dried over Na_2_SO_4_, filtered, and concentrated. The residuals were transferred in a round-bottom flask and distilled under vacuum using a Vigreux column. The products were obtained as colorless to slightly yellowish liquids.

### General Procedure for the Batchwise Synthesis of ILs

The corresponding nucleophile (2 mmol, 1.00 equiv) was transferred to an 8 mL vial, then it was heated up to 80 °C. Once the temperature reached 80 °C, 2 mmol (1.00 equiv) alkyl bistriflimide was added, and the mixture was stirred for 24 h. Then, the mixture was transferred into a round-bottom glass flask, and it was dried under high vacuum at elevated temperature overnight (0.3 mbar, 90 °C).

### General Procedure for the Continuous-Flow Synthesis of ILs

The continuous-flow experiments were performed in a 1000 μL reactor. The nucleophile and the reagent were supplied with the aid of one–one syringe pumps, respectively. The reactor was heated up to the desired temperature with the aid of a magnetic hotplate stirrer, and the syringes were filled with the reagents (7.00 mmol from both the nucleophiles and the alkyl bistriflimides). After starting the pumps, the dead volume of the reactor was allowed to pass. The products were then collected into tared round-bottom flasks for a calculated amount of time. The unreacted starting materials were removed under high vacuum (0.3 mbar) at 90 °C overnight.

## Supporting Information Available

Detailed characterization of the alkyl bistriflimides and ILs via ^1^H-, ^13^C-, and ^19^F NMR spectroscopies, HR-MS spectroscopy, and infrared spectroscopy; halide and water content of the ILs; and kinetic analysis of IL formation (PDF)

## Footnotes

a In the literature, most room-temperature reactions with triflic anhydride are carried out in dichloromethane, while other solvents such as diethyl ether or THF are mostly used at extremely low (−78 °C) temperatures. Importantly, it is only CH_2_Cl_2_ that is inert enough against triflic anhydride at room temperature, as even solvents such as THF react with Tf_2_O under such conditions. To avoid the necessity of cooling, we decided to use dichloromethane but kept the required amount to a minimum due to its unfavorable environmental footprint.

b To suppress the progression of the reaction after the sampling, all samples were immediately cooled to room temperature and diluted with CDCl_3_ after their collection.
