# Supplementary material for: Halide-Free Continuous Synthesis of Hydrophobic Ionic Liquids
Source: ACS Sustain Chem Eng. 2022 Aug 16;10(34):11215–22. doi: 10.1021/acssuschemeng.2c02871 (PMC9428890; doi:10.1021/acssuschemeng.2c02871)
Supplement: Supplementary file 1 — sc2c02871_si_001.pdf [file sc2c02871_si_001.pdf]

Supporting Information

Halide-free Continuous Synthesis of Hydrophobic Ionic Liquids

*Kristof Stagel,[a] Andrea Szpecht,[b] Dawid Zielinski,[b,c] Marcin Smiglak,[b] Michael Schnürch[a] and Katharina Bica-Schröder[a,]**

[a]Institute of Applied Synthetic Chemistry, TU Wien, Getreidemarkt 9/163, Vienna, 1060, Austria

[b]Poznan Science and Technology Park, Adam Mickiewicz University Foundation, st. Rubiez 46, 61-612 Poznan, Poland

[c]Faculty of Chemistry, Adam Mickiewicz University in Poznan, st. Uniwersytetu Poznanskiego 8, 61-614 Poznan, Poland

*Corresponding author: Katharina Bica-Schröder. E-mail: katharina.schroeder@tuwien.ac.at, Tel.: +43 1 58801 163601

Number of pages: 53

Number of figures: 66

Number of tables: 1

Table of Contents

1. General remarks 3
2. Representative protocol for the synthesis of alkyl bistriflimides................................4
3. General procedure for the batch-wise synthesis of ionic liquids 5
4. General procedure for the continuous-flow synthesis of ionic liquids 6
5. Analysis of alkyl bistriflimides...7
6. Analysis of bis(trifluoromethanesulfonyl)imide-based ionic liquids 9
7. Kinetic analysis of ionic liquid formation 15
8. Optimization of the alkyl bistriflimide synthesis 17
9. Batch and continuous-flow synthesis of 2b 18
10. IC measurement parameters, halide- and water content...19
11. E-factor calculations 20
12. NMR spectra of alkyl bistriflimides 22
13. NMR spectra of ionic liquids 30
14. References..53

1. General remarks

All purchased chemicals from commercial suppliers were used without further purification unless otherwise specified. Dry CH_2Cl_2 was pre-distilled and desiccated on aluminum oxide columns (PURESOLV, Innovative Technology).

^{1}H-, ^{13}C- and ^{19}F-NMR spectra were recorded from $CDCl_3$ solutions on a Bruker Avance UltraShield 200 MHz (^{1}H: 200 MHz, ^{13}C: 50 MHz) or 400 MHz (^{1}H: 400 MHz, ^{13}C: 101 MHz, ^{19}F: 376 MHz) NMR instrument. Chemical shifts are reported in parts per million (ppm) and were calibrated to the residual solvent signal ($CDCl_3$, ^{1}H: 7.26 ppm). Coupling constants are reported in hertz. The assignments are based on the comparison with reported spectra.

GC analysis was performed on a Thermo Scientific Focus, on a BGB5 column, using an FID detector. A linear temperature program was used, starting with a temperature of 50 °C, followed by a ramp rate of 15°C/min up to a final temperature of 240 °C. For the optimization of the bistriflimide synthesis, the conversion has been determined by using *n*-dodecane as an internal standard.

HR-MS analysis was performed using a TC PAL system autosampler, an Agilent 1100/1200 HPLC, and an Agilent 6230 AJS ESI-TOF mass spectrometer. The spectra were recorded from methanolic solutions ($c = 3 \times 10^{-5}$ $moldm^{-3}$). The cations ($[C]^+$) of the ILs ($[C]^+[A]^-$) were detected in positive-ion mode.

Infrared spectra were recorded on a Perkin Elmer Spectrum 65 FTIR spectrometer equipped with a Specac MK II Golden Gate Single Reflection ATR unit.

Water content analysis has been performed using a semi-automatic, coulometric Mitsubishi CA-21 Moisture Meter titrator.

Continuous-flow experiments were performed in a 1000 µl CAP DISC reactor. The nucleophile and the reagent were supplied with the aid of one-one New Era NE-1000 syringe pumps, respectively.

2. Representative protocol for the synthesis of alkyl bistriflimides

After 40 mmol (1.00 eq.) of the corresponding amine and *N*,*N*-diisopropylethylamine (82 mmol, 2.05 eq.) were transferred to a 3-neck round-bottom flask, 90 ml anhydrous dichloromethane were added under argon atmosphere. The mixture was stirred and cooled down *via* NaCl/ice bath. Then, trifluoromethanesulfonic anhydride (82 mmol, 2.05 eq.) was dissolved in 20 ml anhydrous dichloromethane and it was added to the mixture dropwise, while the temperature was maintained below 5 °C. Once the triflic anhydride was added, the NaCl/ice bath was removed and the mixture was stirred at room temperature for 1 hour. After that, the mixture was washed once with saturated $NaHCO_3$ solution, once with 2 N HCl solution, and once with distilled water. The aqueous phases were back-extracted with dichloromethane. The combined organic layers were dried over Na_2SO_4, filtered, and concentrated. The residuals were transferred in a round-bottom flask and distilled under vacuum using a Vigreux-column. The products were obtained as colorless to slightly yellowish liquids.

3. General procedure for the batch-wise synthesis of ionic liquids

2 mmol (1.00 eq.) of the corresponding nucleophile was transferred to an 8-ml vial, then it was heated up to 80 °C. Once the temperature reached 80 °C, 2 mmol (1.00 eq.) alkyl bistriflimide was added, and the mixture was stirred for 24 hours. Then, the mixture was transferred into a glass round-bottom flask and it was dried under high vacuum at elevated temperature overnight (0.3 bar, 90 °C).

4. General procedure for the continuous-flow synthesis of ionic liquids

The continuous-flow experiments were performed in a 1000 µl reactor. The nucleophile and the reagent were supplied with the aid of one-one syringe pumps, respectively. The reactor was heated up to the desired temperature with the aid of a magnetic hotplate stirrer and the syringes were filled with the reagents (7.00 mmol from both the nucleophiles and the alkyl bistriflimides). After starting the pumps, the dead volume of the reactor was allowed to pass. The products were then collected into tared round-bottom flasks for a calculated amount of time. The unreacted starting materials were removed under high vacuum (0.3 mbar) at 90 °C overnight.

5. Analysis of alkyl bistriflimides

5.1 *N*-butyl-1,1,1-trifluoro-*N*-((trifluoromethyl)sulfonyl)methanesulfonamide (1a)

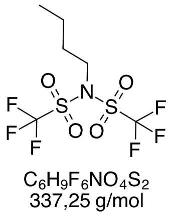

According to the general procedure from chapter 2, product **1a** was obtained as colorless liquid (9.94 g, 74% yield). Boiling point at 24 mbar: 83-85 °C, density:[1] 1.500 gcm^{-1}. **^{1}H NMR** (200 MHz, $CDCl_3$) δ 4.10 – 3.73 (m, 2H, $NC\mathit{H}_2(CH_2)_2CH_3$), 1.80 (ddd, *J* = 15.7, 8.8, 6.4 Hz, 2H, $NCH_2C\mathit{H}_2CH_2CH_3$), 1.48 – 1.17 (m, 2H, $N(CH_2)_2C\mathit{H}_2CH_3$), 0.96 (t, *J* = 7.3 Hz, 3H, $N(CH_2)_3C\mathit{H}_3$). **^{13}C NMR** (101 MHz, $CDCl_3$) δ 123.94, 120.71, 117.48, 114.26, 54.12, 31.73, 19.57, 13.42. **^{19}F NMR** (376 MHz, $CDCl_3$) δ -72.10. **IR** (cm^{-1}): 2970, 2883, 1429, 1214, 119, 855, 605, 504.

5.2 1,1,1-trifluoro-*N*-hexyl-*N*-((trifluoromethyl)sulfonyl)methanesulfonamide (1b)

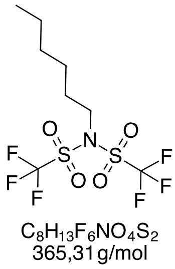

According to the general procedure from chapter 2, product **1b** was obtained as colorless liquid (11.79 g, 81% yield). Boiling point at 18 mbar: 104-107 °C, density:[1] 1.413 gcm^{-1}. **^{1}H NMR** (400 MHz, $CDCl_3$) δ 3.92 (dd, *J* = 9.4, 6.9 Hz, 2H, $NC\mathit{H}_2(CH_2)_4CH_3$), 1.81 (p, *J* = 7.2 Hz, 2H, $NCH_2C\mathit{H}_2(CH_2)_3CH_3$), 1.42 – 1.23 (m, 6H, $NCH_2CH_2(C\mathit{H}_2)_3CH_3$), 0.96 – 0.82 (m, 3H, $N(CH_2)_5C\mathit{H}_3$). **^{13}C NMR** (101 MHz, $CDCl_3$) δ 123.94, 120.71, 117.48, 114.26, 54.35, 31.05, 29.74, 25.92, 22.50, 13.99. **^{19}F NMR** (376 MHz, $CDCl_3$) δ -72.09. **IR** (cm^{-1}): 2935, 2864, 1429, 1214, 1120, 844, 605, 503.

5.3 1,1,1-trifluoro-*N*-octyl-*N*-((trifluoromethyl)sulfonyl)methanesulfonamide (1c)

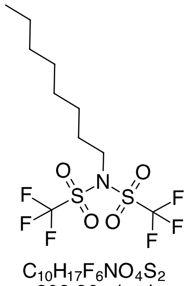

According to the general procedure from chapter 2, product **1c** was obtained as colorless liquid (11.63 g, 74% yield). Boiling point at 0.30 mbar: 80-82 °C, density:[2] 1.310 gcm^{-1}. **^{1}H NMR** (200 MHz, $CDCl_3$) δ 4.04 – 3.76 (m, 2H, $NC\mathit{H}_2CH_2(CH_2)_5CH_3$), 1.81 (p, *J* = 7.0 Hz, 2H, $NCH_2C\mathit{H}_2(CH_2)_5CH_3$), 1.30 (h, *J* = 4.6, 4.0 Hz, 10H, $NCH_2CH_2(C\mathit{H}_2)_5CH_3$), 1.05 – 0.66 (m, 3H, $N(CH_2)_7C\mathit{H}_3$). **^{13}C NMR** (101 MHz, $CDCl_3$) δ 123.94, 120.71, 117.48, 114.26, 54.35, 31.77, 29.78, 29.11, 28.88, 26.25, 22.71, 14.17. **^{19}F NMR** (376 MHz, $CDCl_3$) δ -72.05. **IR** (cm^{-1}): 2930, 2861, 1429, 1216, 1120, 855, 606, 504.

5.4 *N*-decyl-1,1,1-trifluoro-*N*-((trifluoromethyl)sulfonyl)methanesulfonamide (1d)

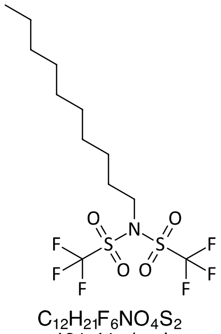

According to the general procedure from chapter 2, product **1d** was obtained as colorless liquid (14.95 g, 89% yield). Boiling point at 0.31 mbar: 92-95 °C, density:[2] 1.22 gcm^{-1}. **^{1}H NMR** (400 MHz, $CDCl_3$) δ 3.97 – 3.83 (m, 2H, $NCH_2CH_2(CH_2)_7CH_3$), 1.88 – 1.73 (m, 2H, $NCH_2CH_2(CH_2)_7CH_3$), 1.39 – 1.14 (m, 14H, $NCH_2CH_2(CH_2)_7CH_3$), 0.95 – 0.81 (m, 3H, $N(CH_2)_9CH_3$). **^{13}C NMR** (101 MHz, $CDCl_3$) δ 123.93, 120.71, 117.48, 114.25, 54.35, 31.98, 29.78, 29.53, 29.44, 29.36, 28.92, 26.25, 22.80, 14.23. **^{19}F NMR** (376 MHz, $CDCl_3$) δ -72.08. **IR** (cm^{-1}): 2928, 2858, 1429, 1216, 1120, 855, 606, 504.

5.5 *N*-dodecyl-1,1,1-trifluoro-*N*-((trifluoromethyl)sulfonyl)methanesulfonamide (1e)

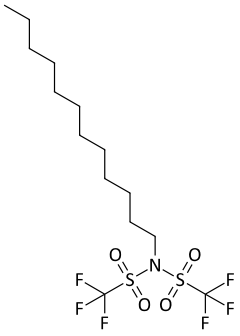

According to the general procedure from chapter 2, product **1e** was obtained as slightly yellowish liquid (15.40 g, 86% yield). Boiling point at 0.35 mbar: 110-115 °C, density:[2] 1.22 gcm^{-1}. **^{1}H NMR** (400 MHz, $CDCl_3$) δ 4.01 – 3.76 (m, 2H, $NCH_2CH_2(CH_2)_9CH_3$), 1.81 (t, *J* = 8.1 Hz, 2H, $NCH_2CH_2(CH_2)_9CH_3$), 1.29 (d, *J* = 18.4 Hz, 18H, $NCH_2CH_2(CH_2)_9CH_3$), 0.99 – 0.77 (m, 3H, $N(CH_2)_{11}CH_3$). **^{13}C NMR** (101 MHz, $CDCl_3$) δ 123.93, 120.70, 117.48, 114.25, 54.35, 32.05, 29.78, 29.72, 29.70, 29.57, 29.46, 29.44, 28.92, 26.25, 22.83, 14.25. **^{19}F NMR** (376 MHz, $CDCl_3$) δ -72.07. **IR** (cm^{-1}): 2926, 2857, 1429, 1217, 1121, 855, 606, 506.

6. Analysis of bis(trifluoromethanesulfonyl)imide-based ionic liquids

6.1 1-Butyl-3-methylimidazolium bis(trifluoromethylsulfonyl)imide (2a)

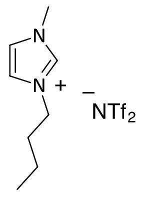

$C_{10}H_{15}F_6N_3O_4S_2$
419,36 g/mol

According to the general procedure from chapter 3, product **2a** was obtained as yellowish liquid (743 mg, 89% yield). **^{1}H NMR** (400 MHz, $CDCl_3$) δ 8.74 (q, *J* = 2.0 Hz, 1H, NC*H*N), 7.30 (dt, *J* = 4.8, 1.9 Hz, 2H, NC*H*C*H*N), 4.16 (t, *J* = 7.5 Hz, 2H, NC*H2*$(CH_2)_2CH_3$), 3.93 (d, *J* = 1.2 Hz, 3H, NC*H3*), 1.84 (tt, *J* = 8.3, 6.9 Hz, 2H, NCH_2C*H2*CH_2CH_3), 1.35 (dt, *J* = 14.7, 7.5 Hz, 2H, NCH_2CH_2C*H2*CH_3), 0.95 (t, *J* = 7.3 Hz, 3H, $NCH_2CH_2CH_2C$*H3*). **^{13}C NMR** (101 MHz, $CDCl_3$) δ 136.24, 123.79, 122.36, 121.52, 118.33, 50.09, 36.45, 32.04, 19.45, 13.32. **^{19}F NMR** (376 MHz, $CDCl_3$) δ -79.09. **HRMS** [C^+] calc. 139.1230, found 139.1235.

6.2 1-Hexyl-3-methylimidazolium bis(trifluoromethylsulfonyl)imide (2b)

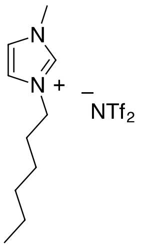

$C_{12}H_{19}F_6N_3O_4S_2$
447,41 g/mol

According to the general procedure from chapter 3, product **2b** was obtained as yellowish liquid (811 mg, 91% yield). **^{1}H NMR** (400 MHz, $CDCl_3$) δ 8.77 (d, *J* = 7.0 Hz, 1H, NC*H*N), 7.30 (dq, *J* = 9.1, 1.8 Hz, 2H, NC*H*C*H*N), 4.31 – 4.13 (m, 2H, NC*H2*$(CH_2)_4CH_3$), 3.94 (d, *J* = 1.8 Hz, 3H, NC*H3*), 1.95 – 1.79 (m, 2H, NCH_2C*H2*$(CH_2)_3CH_3$), 1.31 (q, *J* = 4.1, 2.9 Hz, 6H, NCH_2CH_2(C*H2*)$_3CH_3$), 0.98 – 0.77 (m, 3H, $N(CH_2)_5C$*H3*). **^{13}C NMR** (101 MHz, $CDCl_3$) δ 136.26, 123.80, 122.32, 121.52, 118.33, 50.36, 36.47, 31.05, 30.12, 25.86, 22.40, 13.93. **^{19}F NMR** (376 MHz, $CDCl_3$) δ -79.05. **HRMS** [C^+] calc. 167.1543, found 167.1544.

6.3 1-Octyl-3-methylimidazolium bis(trifluoromethylsulfonyl)imide (2c)

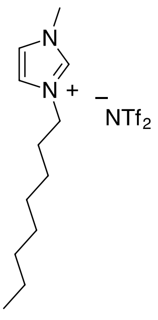

$C_{14}H_{23}F_6N_3O_4S_2$
475,47 g/mol

According to the general procedure from chapter 3, product **2c** was obtained as slightly yellowish liquid (843 mg, 89% yield). **^{1}H NMR** (400 MHz, $CDCl_3$) δ 8.76 (s, 1H, NC*H*N), 7.29 (dt, *J* = 11.1, 1.7 Hz, 2H, NC*H*C*H*N), 4.29 – 4.07 (m, 2H, NC*H2*$(CH_2)_6CH_3$), 3.94 (d, *J* = 1.6 Hz, 3H, NC*H3*), 1.94 – 1.78 (m, 2H, NCH_2C*H2*$(CH_2)_5CH_3$), 1.43 – 1.12 (m, 10H, NCH_2CH_2(C*H2*)$_5CH_3$), 0.92 – 0.78 (m, 3H, $N(CH_2)_7C$*H3*). **^{13}C NMR** (101 MHz, $CDCl_3$) δ 124.72, 123.80, 122.29, 121.53, 118.34, 50.41, 36.52, 31.74, 30.19, 29.05, 28.92, 26.24, 22.67, 14.14. **^{19}F NMR** (376 MHz, $CDCl_3$) δ -79.04. **HRMS** [C^+] calc. 195.1856, found 195.1860.

6.4 1-Decyl-3-methylimidazolium bis(trifluoromethylsulfonyl)imide (2d)

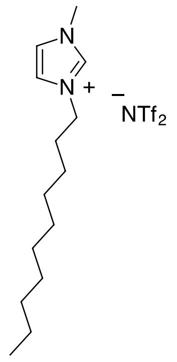

$C_{16}H_{27}F_6N_3O_4S_2$
503,52 g/mol

According to the general procedure from chapter 3, product **2d** was obtained as yellowish liquid (883 mg, 88% yield). **^{1}H NMR** (400 MHz, $CDCl_3$) δ 8.78 (s, 1H, NC*H*N), 7.32 – 7.26 (m, 2H, NC*H*C*H*N), 4.16 (td, *J* = 7.6, 2.4 Hz, 2H, $NC\mathit{H}_2(CH_2)_8CH_3$), 3.95 (d, *J* = 2.9 Hz, 3H, $NC\mathit{H}_3$), 1.86 (h, *J* = 6.4, 5.9 Hz, 2H, $NCH_2C\mathit{H}_2(CH_2)_7CH_3$), 1.28 (d, *J* = 26.6 Hz, 14H, $NCH_2CH_2(C\mathit{H}_2)_7CH_3$), 0.87 (t, *J* = 6.8 Hz, 3H, $N(CH_2)_9C\mathit{H}_3$). **^{13}C NMR** (101 MHz, $CDCl_3$) δ 136.37, 123.76, 122.25, 121.53, 118.34, 50.43, 36.55, 31.95, 30.20, 29.52, 29.41, 29.34, 28.98, 26.26, 22.78, 14.21. **^{19}F NMR** (376 MHz, $CDCl_3$) δ -79.01. **HRMS** [C^+] calc. 223.2169, found 223.2175.

6.5 1-Dodecyl-3-methylimidazolium bis(trifluoromethylsulfonyl)imide (2e)

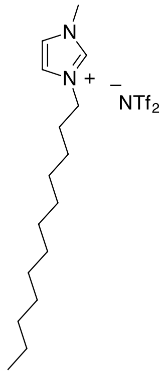

$C_{18}H_{31}F_6N_3O_4S_2$
531,57 g/mol

According to the general procedure from chapter 3, product **2e** was obtained as yellowish liquid (879 mg, 83% yield). **^{1}H NMR** (400 MHz, $CDCl_3$) δ 8.77 (d, *J* = 1.9 Hz, 1H, NC*H*N), 7.29 (dt, *J* = 12.5, 1.9 Hz, 2H, NC*H*C*H*N), 4.16 (dd, *J* = 8.2, 6.8 Hz, 2H, $NC\mathit{H}_2(CH_2)_{10}CH_3$), 3.94 (s, 3H, $NC\mathit{H}_3$), 1.86 (p, *J* = 7.4 Hz, 2H, $NCH_2C\mathit{H}_2(CH_2)_9CH_3$), 1.36 – 1.11 (m, 18H, $NCH_2CH_2(C\mathit{H}_2)_9CH_3$), 0.94 – 0.80 (m, 3H, $N(CH_2)_{11}C\mathit{H}_3$). **^{13}C NMR** (101 MHz, $CDCl_3$) δ 136.34, 123.77, 122.26, 121.52, 118.33, 50.41, 36.52, 32.03, 30.20, 29.71, 29.70, 29.58, 29.45, 29.42, 28.99, 26.26, 22.81, 14.23. **^{19}F NMR** (376 MHz, $CDCl_3$) δ -79.02. **HRMS** [C^+] calc. 251.2482, found 251.2485.

6.6 1-Butylpyridinium bis(trifluoromethylsulfonyl)imide (3a)

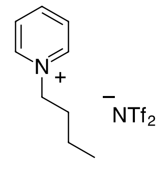

$C_{11}H_{14}F_6N_2O_4S_2$
416,36 g/mol

According to the general procedure from chapter 3, product **3a** was obtained as slightly yellowish liquid (764 mg, 92% yield). **^{1}H NMR** (400 MHz, $CDCl_3$) δ 9.02 – 8.69 (m, 2H, 2 x CH(CH)(C*H*)N, *H*-arom), 8.47 (tt, *J* = 7.8, 1.4 Hz, 1H, C*H*(CH)(CH)N, *H*-arom), 8.06 (t, *J* = 7.0 Hz, 2H, 2 x CH(C*H*)(CH)N, *H*-arom), 4.74 – 4.41 (m, 2H, $NC\mathit{H}_2(CH_2)_2CH_3$), 1.99 (tt, *J* = 9.4, 6.8 Hz, 2H, $NCH_2C\mathit{H}_2CH_2CH_3$), 1.40 (dq, *J* = 14.9, 7.4 Hz, 2H, $NCH_2CH_2C\mathit{H}_2CH_3$), 0.98 (t, *J* = 7.4 Hz, 3H, $N(CH_2)_2C\mathit{H}_3$). **^{13}C NMR** (101 MHz, $CDCl_3$) δ 145.53, 144.54, 128.80, 121.52, 118.33, 62.62, 33.53, 19.40, 13.34. **^{19}F NMR** (376 MHz, $CDCl_3$) δ -78.92. **HRMS** [C^+] calc. 136.1121, found 136.1127.

6.7 1-Hexylpyridinium bis(trifluoromethylsulfonyl)imide (3b)

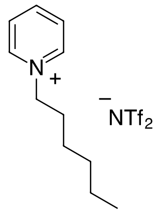

$C_{13}H_{18}F_6N_2O_4S_2$
444,41 g/mol

According to the general procedure from chapter 3, product **3b** was obtained as slightly yellowish liquid (814 mg, 92% yield). **^{1}H NMR** (400 MHz, $CDCl_3$) δ 8.90 – 8.72 (m, 2H, 2 x CH(CH)(C*H*)N, *H*-arom), 8.47 (tt, *J* = 7.8, 1.4 Hz, 1H, C*H*(CH)(CH)N, *H*-arom), 8.18 – 7.93 (m, 2H, 2 x CH(C*H*)(CH)N, *H*-arom), 4.60 (t, *J* = 7.6 Hz, 2H, $NCH_2(CH_2)_4CH_3$), 2.09 – 1.88 (m, 2H, $NCH_2CH_2(CH_2)_3CH_3$), 1.44 – 1.18 (m, 6H, $NCH_2CH_2(CH_2)_3CH_3$), 0.93 – 0.76 (m, 3H, $N(CH_2)_5CH_3$). **^{13}C NMR** (101 MHz, $CDCl_3$) δ 145.56, 144.52, 124.72, 121.53, 118.33, 62.76, 31.63, 31.01, 25.69, 22.35, 13.89. **^{19}F NMR** (376 MHz, $CDCl_3$) δ -78.93. **HRMS** [C^+] calc. 164.1434, found 164.1437.

6.8 1-Octylpyridinium bis(trifluoromethylsulfonyl)imide (3c)

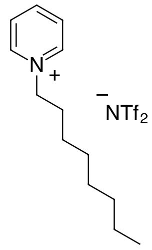

$C_{15}H_{22}F_6N_2O_4S_2$
472,46 g/mol

According to the general procedure from chapter 3, product **3c** was obtained as colorless liquid (817 mg, 87% yield). **^{1}H NMR** (400 MHz, $CDCl_3$) δ 8.89 – 8.74 (m, 2H, 2x CH(CH)(C*H*)N, *H*-arom), 8.47 (tt, *J* = 7.8, 1.4 Hz, 1H, C*H*(CH)(CH)N, *H*-arom), 8.15 – 7.95 (m, 2H, 2 x CH(C*H*)(CH)N, *H*-arom), 4.59 (td, *J* = 7.5, 1.3 Hz, 2H, $NCH_2(CH_2)_6CH_3$), 1.99 (q, *J* = 7.5 Hz, 2H, $NCH_2CH_2(CH_2)_5CH_3$), 1.46 – 1.09 (m, 10H, $NCH_2CH_2(CH_2)_5CH_3$), 0.91 – 0.79 (m, 3H, $N(CH_2)_7CH_3$). **^{13}C NMR** (101 MHz, $CDCl_3$) δ 145.54, 144.52, 128.80, 121.53, 118.33, 62.84, 31.69, 28.99, 28.90, 26.07, 22.65, 14.12. **^{19}F NMR** (376 MHz, $CDCl_3$) δ -78.93. **HRMS** [C^+] calc. 192.1747, found 192.1751.

6.9 1-Decylpyridinium bis(trifluoromethylsulfonyl)imide (3d)

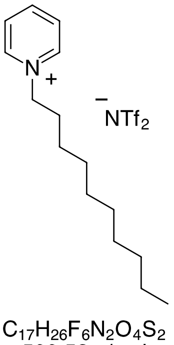

$C_{17}H_{26}F_6N_2O_4S_2$
500,52 g/mol

According to the general procedure from chapter 3, product **3d** was obtained as slightly brownish, viscous liquid (910 mg, 91% yield). **^{1}H NMR** (400 MHz, $CDCl_3$) δ 8.89 – 8.69 (m, 2H, 2 x CH(CH)(C*H*)N, *H*-arom), 8.48 (d, *J* = 7.9 Hz, 1H, C*H*(CH)(CH)N, *H*-arom), 8.06 (t, *J* = 7.0 Hz, 2H, 2 x CH(C*H*)(CH)N, *H*-arom), 4.69 – 4.49 (m, 2H, $NCH_2(CH_2)_8CH_3$), 2.00 (t, *J* = 7.4 Hz, 2H, $NCH_2CH_2(CH_2)_7CH_3$), 1.29 (d, *J* = 37.9 Hz, 14H, $NCH_2CH_2(CH_2)_7CH_3$), 0.87 (t, *J* = 6.8 Hz, 3H, $N(CH_2)_9CH_3$). **^{13}C NMR** (101 MHz, $CDCl_3$) δ 145.54, 144.52, 128.81, 121.53, 118.34, 62.87, 31.94, 31.71, 29.49, 29.36, 29.32, 28.96, 26.10, 22.77, 14.21. **^{19}F NMR** (376 MHz, $CDCl_3$) δ -78.91. **HRMS** [C^+] calc. 220.2060, found 220.2064.

6.10 1-Dodecylpyridinium bis(trifluoromethylsulfonyl)imide (3e)

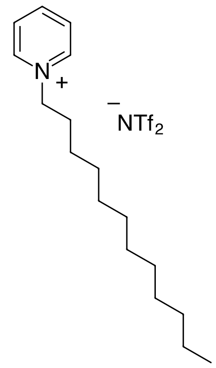

$C_{19}H_{30}F_6N_2O_4S_2$
528,57 g/mol

According to the general procedure from chapter 3, product **3e** was obtained as slightly yellowish, viscous liquid (887 mg, 84% yield). **^{1}H NMR** (400 MHz, $CDCl_3$) δ 8.87 – 8.74 (m, 2H, 2 x CH(CH)(C*H*)N, *H*-arom), 8.47 (tt, *J* = 7.8, 1.4 Hz, 1H, C*H*(CH)(CH)N, *H*-arom), 8.11 – 8.00 (m, 2H, 2 x CH(C*H*)(CH)N, *H*-arom), 4.59 (t, *J* = 7.6 Hz, 2H, $NC\mathit{H_2}(CH_2)_{10}CH_3$), 1.99 (m, 2H, $NCH_2C\mathit{H_2}(CH_2)_9CH_3$), 1.38 – 1.18 (m, 18H, $NCH_2CH_2(C\mathit{H_2})_9CH_3$), 0.93 – 0.77 (m, 3H, $N(CH_2)_{11}C\mathit{H_3}$). **^{13}C NMR** (101 MHz, $CDCl_3$) δ 145.54, 144.51, 128.80, 121.52, 118.32, 62.86, 32.02, 31.70, 29.69, 29.67, 29.54, 29.44, 29.37, 28.97, 26.10, 22.80, 14.23. **^{19}F NMR** (376 MHz, $CDCl_3$) δ -78.92. **HRMS** [C^+] calc. 248.2373, found 248.2370.

6.11 1-Butyl-3-vinylimidazolium bis(trifluoromethylsulfonyl)imide (5a)

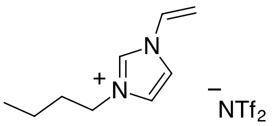

$C_{11}H_{15}F_6N_3O_4S_2$
431,37 g/mol

According to the general procedure from chapter 3, product **5a** was obtained as yellowish liquid (767 mg, 89% yield). **^{1}H NMR** (400 MHz, $CDCl_3$) δ 9.03 (d, *J* = 1.9 Hz, 1H, NC*H*N), 7.62 (d, *J* = 1.9 Hz, 1H, NC*H*CHN), 7.44 (d, *J* = 1.9 Hz, 1H, NCHC*H*), 7.13 (dd, *J* = 15.6, 8.6 Hz, 1H, $NC\mathit{H}CH_2$), 5.78 (dd, *J* = 15.6, 3.1 Hz, 1H, $NCHC\mathit{H_2}$), 5.43 (dd, *J* = 8.7, 3.1 Hz, 1H, $NCHC\mathit{H_2}$), 4.23 (t, *J* = 7.5 Hz, 2H, $NC\mathit{H_2}(CH_2)_2CH_3$), 1.87 (tt, *J* = 9.3, 6.9 Hz, 2H, $NCH_2C\mathit{H_2}CH_2CH_3$), 1.37 (dt, *J* = 14.8, 7.4 Hz, 2H, $NCH_2CH_2C\mathit{H_2}CH_3$), 0.96 (t, *J* = 7.4 Hz, 3H, $N(CH_2)_3C\mathit{H_3}$). **^{13}C NMR** (101 MHz, $CDCl_3$) δ 134.60, 128.10, 123.22, 121.51, 119.39, 118.32, 110.42, 50.47, 31.99, 19.44, 13.31. **^{19}F NMR** (376 MHz, $CDCl_3$) δ -79.02. **HRMS** [C^+] calc. 151.1230, found 151.1235.

6.12 1-Hexyl-3-vinylimidazolium bis(trifluoromethylsulfonyl)imide (5b)

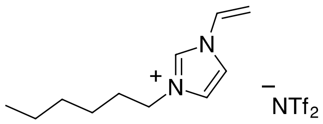

$C_{13}H_{19}F_6N_3O_4S_2$
459,42 g/mol

According to the general procedure from chapter 3, product **5b** was obtained as yellow liquid (889 mg, 97% yield). **^{1}H NMR** (400 MHz, $CDCl_3$) δ 9.09 (q, *J* = 1.6 Hz, 1H, NC*H*N), 7.60 (t, *J* = 2.0 Hz, 1H, NC*H*CHN), 7.41 (d, *J* = 2.0 Hz, 1H, NCHC*H*N), 7.15 (dd, *J* = 15.6, 8.6 Hz, 1H, $NC\mathit{H}CH_2$), 5.78 (dd, *J* = 15.6, 3.1 Hz, 1H, $NCHC\mathit{H_2}$), 5.45 (dd, *J* = 8.6, 3.1 Hz, 1H, $NCHC\mathit{H_2}$), 4.30 – 4.15 (m, 2H, $NC\mathit{H_2}(CH_2)_4CH_3$), 1.97 – 1.80 (m, 2H, $NCH_2C\mathit{H_2}(CH_2)_3CH_3$), 1.33 (tdt, *J* = 8.5, 5.6, 3.5 Hz, 6H, $NCH_2CH_2(C\mathit{H_2})_3CH_3$), 0.97 – 0.78 (m, 3H, $N(CH_2)_5C\mathit{H_3}$). **^{13}C NMR** (101 MHz, $CDCl_3$) δ 134.73, 128.12, 123.13, 121.52, 119.32, 118.33, 110.43, 50.75, 31.05, 30.09, 25.84, 22.40, 13.93. **^{19}F NMR** (376 MHz, $CDCl_3$) δ -78.96. **HRMS** [C^+] calc. 179.1543, found 179.1547.

6.13 1-Octyl-3-vinylimidazolium bis(trifluoromethylsulfonyl)imide (5c)

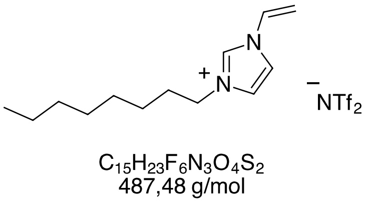

According to the general procedure from chapter 3, product **5c** was obtained as yellowish liquid (906 mg, 93% yield). **^{1}H NMR** (400 MHz, $CDCl_3$) δ 9.06 (s, 1H, NC*H*N), 7.65 – 7.56 (m, 1H, NC*H*CHN), 7.45 – 7.35 (m, 1H, NCHC*H*N), 7.20 – 7.08 (m, 1H, NC*H*CH_2), 5.78 (dt, *J* = 15.5, 2.5 Hz, 1H, NCHCH_2), 5.49 – 5.30 (m, 1H, NCHCH_2), 4.24 (dt, *J* = 11.5, 5.9 Hz, 2H, NC$H_2$$(CH_2)_6CH_3$), 1.87 (s, 2H, $NCH_2CH_2(CH_2)_5CH_3$), 1.50 – 1.13 (m, 10H, $NCH_2CH_2(CH_2)_5CH_3$), 0.94 – 0.77 (m, 3H, $N(CH_2)_7CH_3$). **^{13}C NMR** (101 MHz, $CDCl_3$) δ 128.14, 124.72, 123.11, 121.53, 119.33, 118.33, 110.47, 50.79, 31.73, 30.16, 29.04, 28.92, 26.21, 22.67, 14.14. **^{19}F NMR** (376 MHz, $CDCl_3$) δ -78.97. **HRMS** [C^+] calc. 207.1856, found 207.1862.

6.14 1-Decyl-3-vinylimidazolium bis(trifluoromethylsulfonyl)imide (5d)

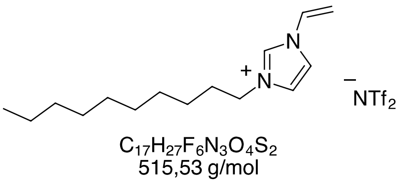

According to the general procedure from chapter 3, product **5d** was obtained as yellowish liquid (883 mg, 93% yield). **^{1}H NMR** (400 MHz, $CDCl_3$) δ 9.10 (s, 1H, NC*H*N), 7.64 – 7.55 (m, 1H, NC*H*CHN), 7.39 (d, *J* = 1.9 Hz, 1H, NCHC*H*N), 7.16 (dd, *J* = 15.6, 8.7 Hz, 1H, NC*H*CH_2), 5.77 (dd, *J* = 15.6, 3.1 Hz, 1H, NCHCH_2), 5.45 (dd, *J* = 8.6, 3.1 Hz, 1H, NCHCH_2), 4.29 – 4.13 (m, 2H, $NCH_2(CH_2)_8CH_3$), 1.90 (q, *J* = 6.7, 6.1 Hz, 2H, $NCH_2CH_2(CH_2)_7CH_3$), 1.41 – 1.10 (m, 14H, $NCH_2CH_2(CH_2)_7CH_3$), 0.93 – 0.77 (m, 3H, $N(CH_2)_9CH_3$). **^{13}C NMR** (101 MHz, $CDCl_3$) δ 134.99, 128.15, 122.96, 121.53, 119.16, 118.34, 110.50, 50.82, 31.95, 30.17, 29.52, 29.40, 29.34, 28.98, 26.23, 22.78, 14.21. **^{19}F NMR** (376 MHz, $CDCl_3$) δ -78.93. **HRMS** [C^+] calc. 235.2169, found 235.2174.

6.15 1-Dodecyl-3-vinylimidazolium bis(trifluoromethylsulfonyl)imide (5e)

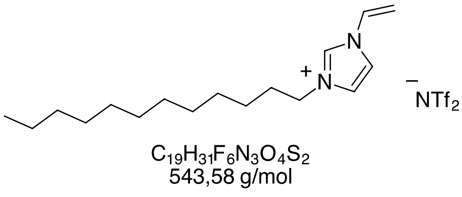

According to the general procedure from chapter 3, product **5e** was obtained as brownish, viscous liquid (984 mg, 91% yield). **^{1}H NMR** (400 MHz, $CDCl_3$) δ 9.11 – 9.00 (m, 1H, NC*H*N), 7.61 (d, *J* = 1.9 Hz, 1H, NC*H*CHN), 7.40 (t, *J* = 1.9 Hz, 1H, NCHC*H*N), 7.15 (dd, *J* = 15.6, 8.7 Hz, 1H, NC*H*CH_2), 5.78 (dd, *J* = 15.6, 3.1 Hz, 1H, NCHCH_2), 5.44 (dd, *J* = 8.6, 3.1 Hz, 1H, NCHCH_2), 4.23 (t, *J* = 7.5 Hz, 2H, $NCH_2(CH_2)_{10}CH_3$), 1.89 (p, *J* = 7.8 Hz, 2H, $NCH_2CH_2(CH_2)_9CH_3$), 1.42 – 1.15 (m, 18H, $NCH_2CH_2(CH_2)_9CH_3$), 0.93 – 0.79 (m, 3H, $N(CH_2)_9CH_3$). **^{13}C NMR** (101 MHz, $CDCl_3$) δ 134.80, 128.14, 123.05, 121.51, 119.27, 118.32, 110.47, 50.79, 32.03, 30.17, 29.71,

29.69, 29.57, 29.45, 29.41, 28.99, 26.23, 22.81, 14.23. **^{19}F NMR** (376 MHz, $CDCl_3$) δ -78.96. **HRMS** [C^+] calc. 263.2482, found 263.2497.

7. Kinetic analysis of ionic liquid formation

For the kinetic analysis of the ionic liquid formation, 2 mmol substrate (in the given example: 1-methylimidazole, 164 mg, 159 µl) were heated up to 80 °C in a glass vial, then 1.30 equivalent (2.60 mmol, 878 mg) butyl-bistriflimide were added. Subsequently, samples (20µl) were taken at the following intervals: 0.5 min, 5 min, 10 min, 15 min, 20 min, 25 min, 30 min, 45 min, 60 min, 90 min, 120 min, 180 min, and 1440 min. The collected samples were immediately cooled down to room temperature and diluted with 0.6 ml $CDCl_3$. The conversion was calculated using the following equation:

$$\frac{\int product}{(\int product + \int educt)} \times 100 \ = conversion\ [\%]$$

An example of the synthesis of 1-butyl-3-methylimidazolium bistriflimide can be seen in **Figure S1** and **Figure S2**.

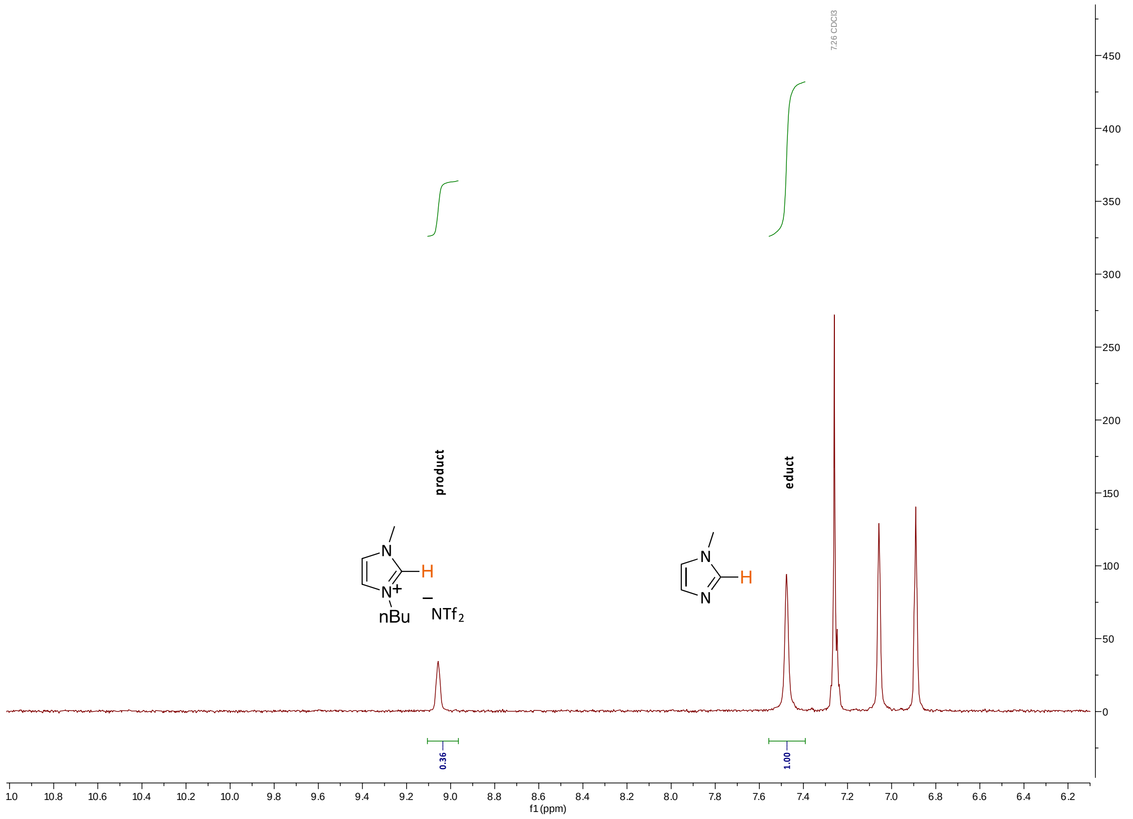

Figure S1. Calculation of conversion for the batch-wise synthesis of IL **2a** (sample taken after 0.5 minutes)

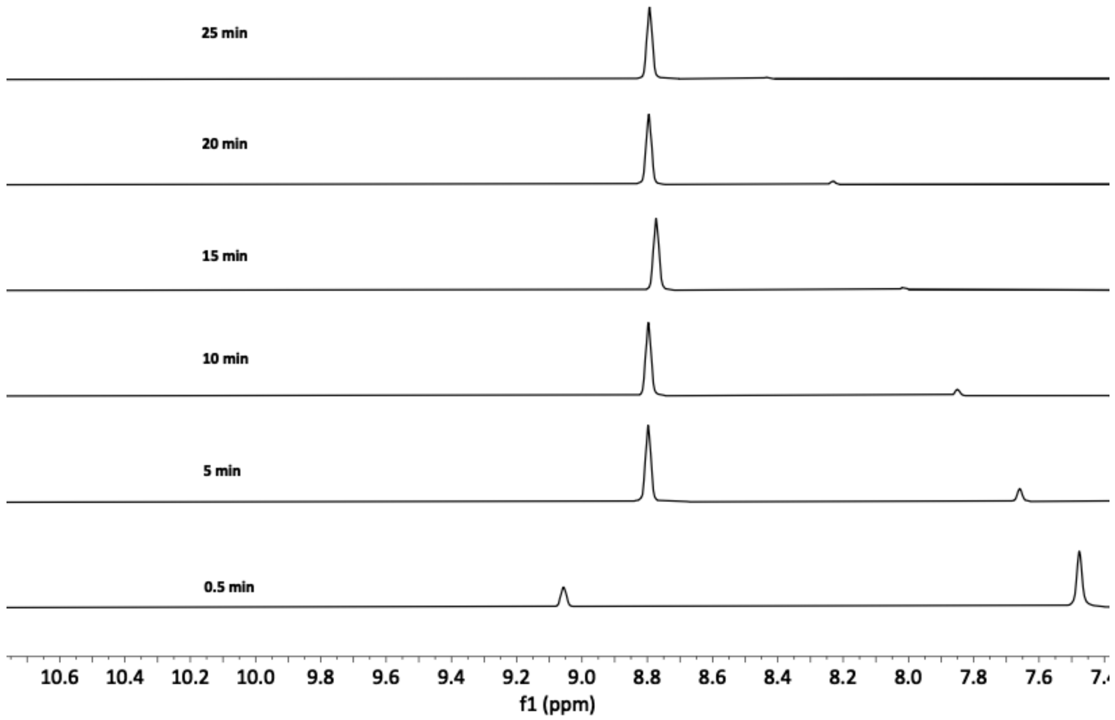

Figure S2. IL formation monitored by ^{1}H-NMR spectroscopy

8. Optimization of the alkyl bistriflimide synthesis

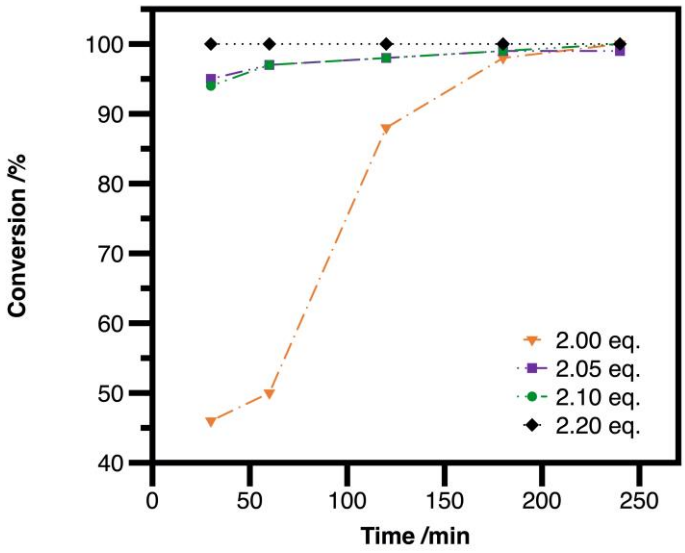

Figure S3. Optimization of the ratio of triflic anhydride by using *n*-butylamine as substrate

9. Batch and continuous-flow synthesis of 2b

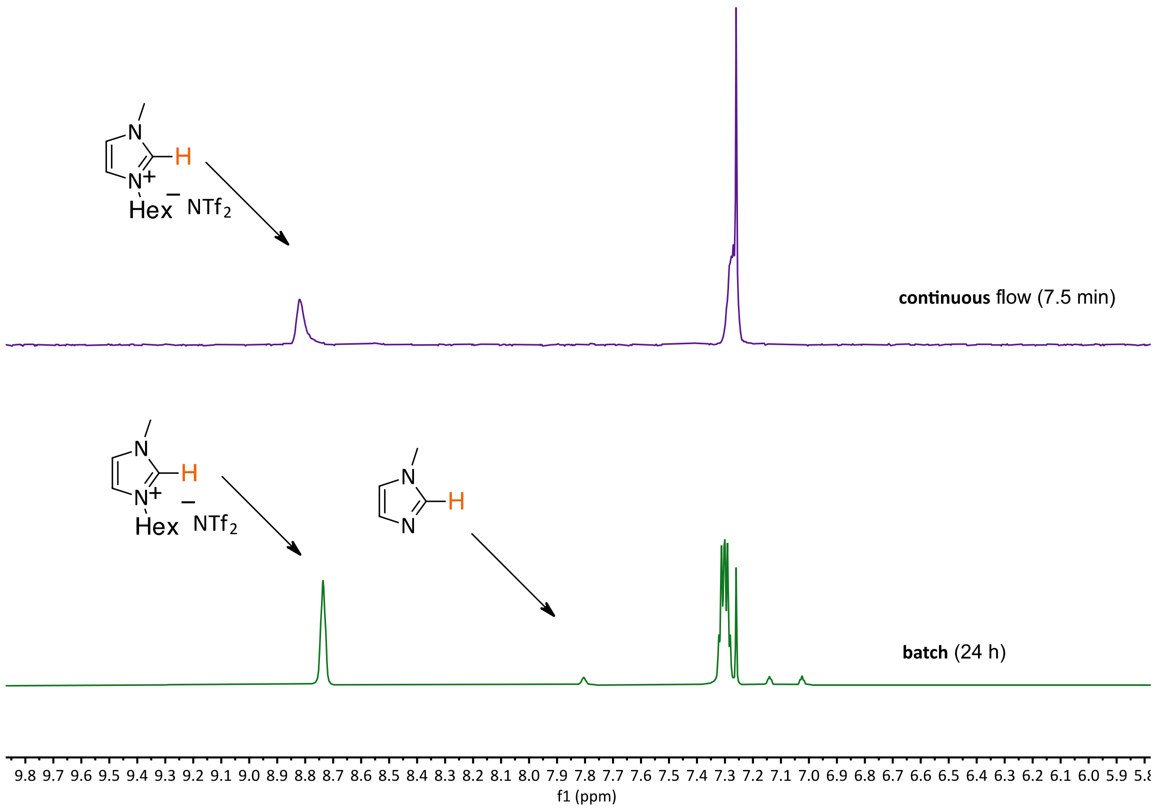

Figure S4. Conversion earned under batch-wise and continuous-flow conditions at 120 °C

Both the batch-wise and the continuous-flow experiments were carried out at 120 °C. As it can be seen, IL **2b** has not yielded full conversion after 24 hours under batch-wise conditions (green), whereas full conversion could be reached in 7.5 minutes when the continuous-flow reactor (purple) was employed.

10. IC measurement parameters, halide- and water content

Ion chromatography experiments were carried out using a Metrohm Eco IC system (Metrohm, Herisau, Switzerland) equipped with 863 Compact IC Autosampler, 10.0 µL injection loop, and conductometric detector (maintained at room temperature). Self-regenerating Suppressor Module (MSM) (Metrohm, Herisau, Switzerland), regenerated with distilled water and sulfuric acid, was used to separate and determine anions in ionic liquids. All data were recorded by Metrohm software. Anion ion chromatograph apparatus was equipped with a Metrosep A Supp. 5 ion exchange column (150 × 4.0 mm) coupled with Metrosep A Supp. Guard. A flow rate of 0.9 mL min^{-1} was used in both systems. Anion separation was performed with eluent composed of a 30:70 ratio of acetonitrile and an aqueous solution containing 3.2mM of sodium bicarbonate and 1.0 mM of sodium carbonate (Merck). All aqueous solutions were prepared carefully using distilled water (σ = 0.05 µS cm^{-1}). During anion separation, average pressure in the analytical system was maintained at the level of 13.5 MPa. Samples for anion analysis were first dissolved in 1 mL of acetone (HPLC grade, Merck), then the sample was taken for analysis (200 µL) and then complemented in a vial with 5 mL of the eluent.

Table S1. Halide- and water content of the produced ionic liquids

Entry	[Cl^-] content /ppm[a]	Water content /ppm[b]
**2a**	150	356
**2b**	140	359
**2c**	140	386
**2d**	490	312
**2e**	120	376
**3a**	450	132
**3b**	100	154
**3c**	220	267
**3d**	260	112
**3e**	340	121
**4a**	N/A	268
**4b**	160	198
**4c**	310	182
**4d**	490	239
**4e**	445	288

[a] Determined by IC analysis. [b] Determined by Karl Fischer-titration, the given value is the average of 3 consecutive measurements, samples were

measured after they were dried on high vacuum (0.3 mbar) at 90 °C for overnight.

11. E-factor calculations

11.1 State-of-the-art method

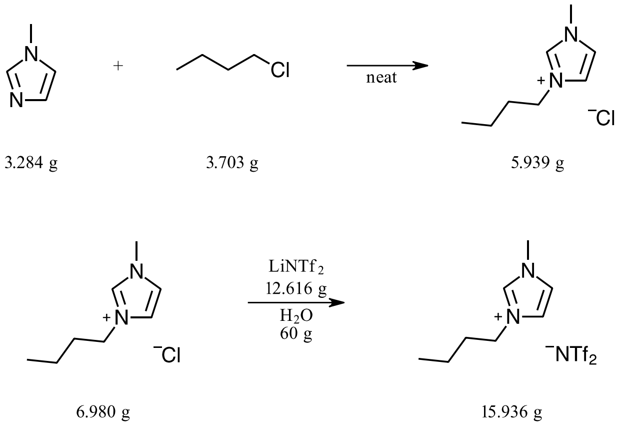

Figure S5. State-of-the-art synthesis of NTf_2^--based ionic liquids

Working on a 40 mmolar scale, assuming the use of 3 x 10 g ethyl acetate for washing the formed IL, 30 g ethyl acetate: acetonitrile (1:1) mixture for the recrystallization of the chloride IL and 85% yield, the calculated E-factor for the first step:

$$E - factor = \frac{3.284 + 3.703 + 3\,x\,10 + 30 - 5.939}{5.939} = 10.28$$

Working on a 40 mmolar scale, assuming the use of 3 x 30 g dichloromethane for extraction of the formed IL, 10 x 40 g distilled water for the washing of the IL, 5 g of Na_2SO_4 for drying and 95% yield, the calculated E-factor for the second step:

$$E - factor = \frac{6.980 + 12.616 + 60 + 3\,x\,30 + 10\,x\,40 + 5 - 15.936}{15.936} = 35.06$$

The summarized E-factor over 2 steps:

$$\sum E - factor = 45.34$$

11.2 Halide-free alkyl bistriflimide-based method

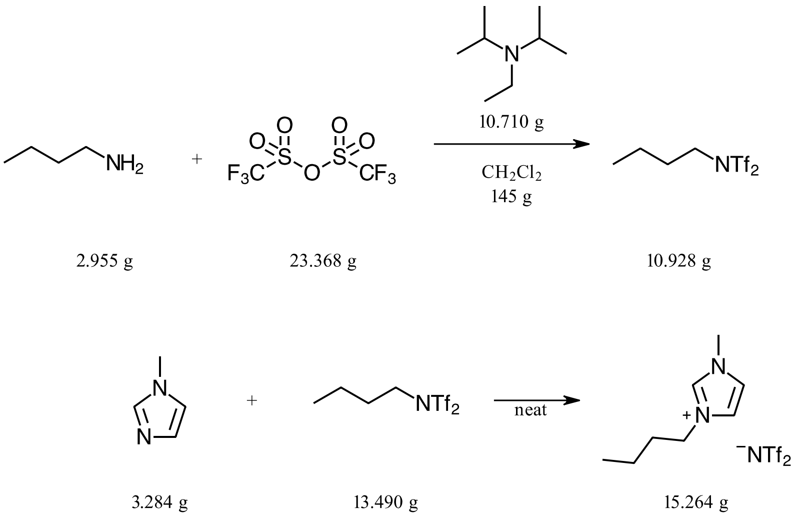

Figure S6. Halide-free synthesis of NTf_2^--based ionic liquids

Working on a 40 mmolar scale, assuming the use of 40 g sat. $NaHCO_3$ solution, 40 g 1M HCl solution and 40 g water for the washing of the alkyl bistriflimide containing crude mixture, 3 x 10 g dichloromethane for the back-extraction of the aqueous phases, 5 g Na_2SO_4 for drying and 81% yield, the calculated E-factor for the first step: (plus by-product: 31.78)

$$E - factor = \frac{2.955 + 23.368 + 10.710 + 145 + 3\,x\,40 + 3\,x\,10 + 5 - 10.928}{10.928} = 29.84$$

Working on a 40 mmolar scale, assuming no use of any further chemicals and 91% yield, the calculated E-factor for the second step:

$$E - factor = \frac{3.284 + 13.490 - 15.264}{15.264} = 0.01$$

The summarized E-factor over 2 steps:

$$\sum E - factor = 29.85$$

Based on the comparison of the calculated E-factor values, our bistriflimide-based method generates ca. 15 kg less waste pro kg product.

12. NMR spectra of alkyl bistriflimides

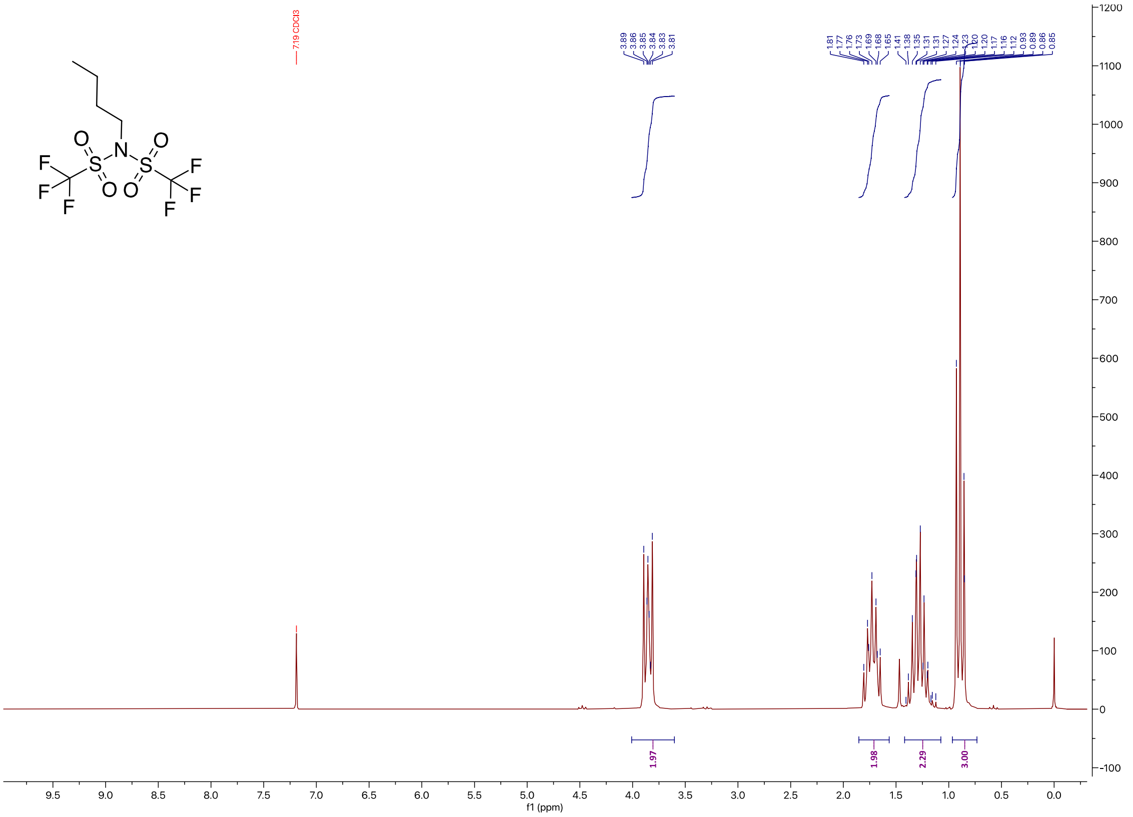

Figure S7. ^{1}H-NMR spectrum of **1a**

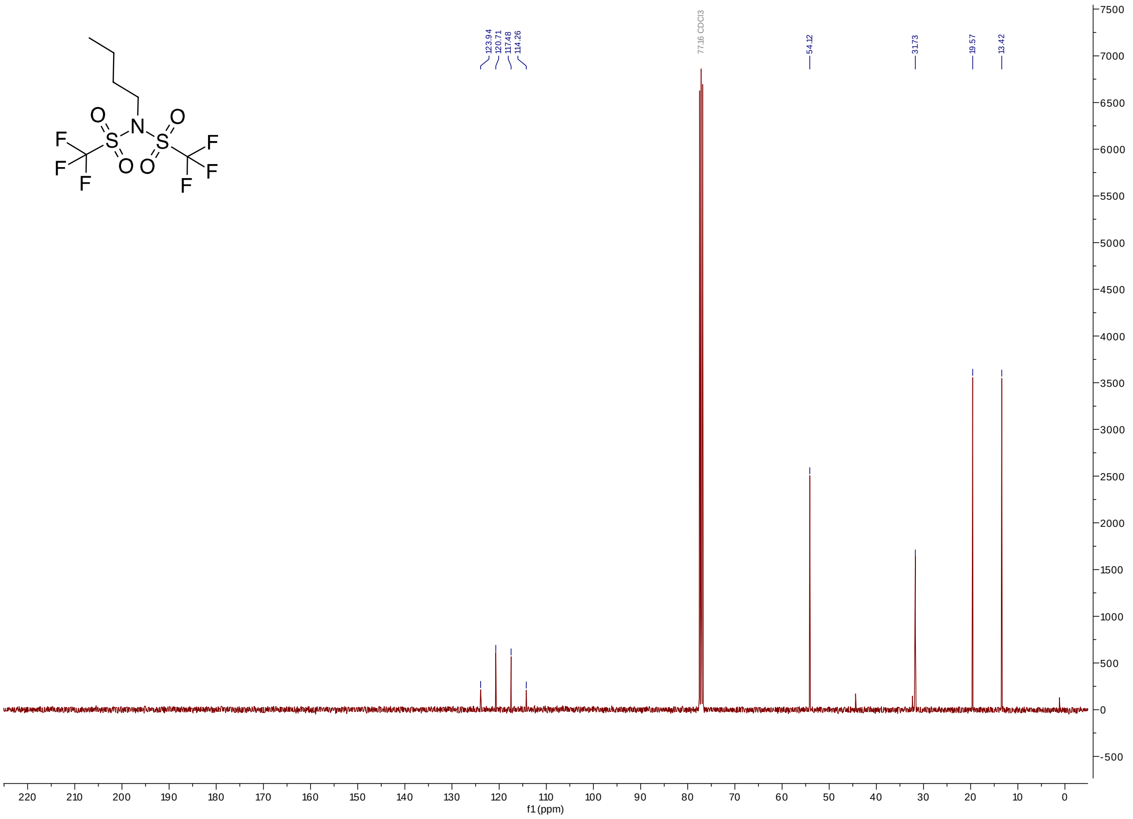

Figure S8. ^{13}C-NMR spectrum of **1a**

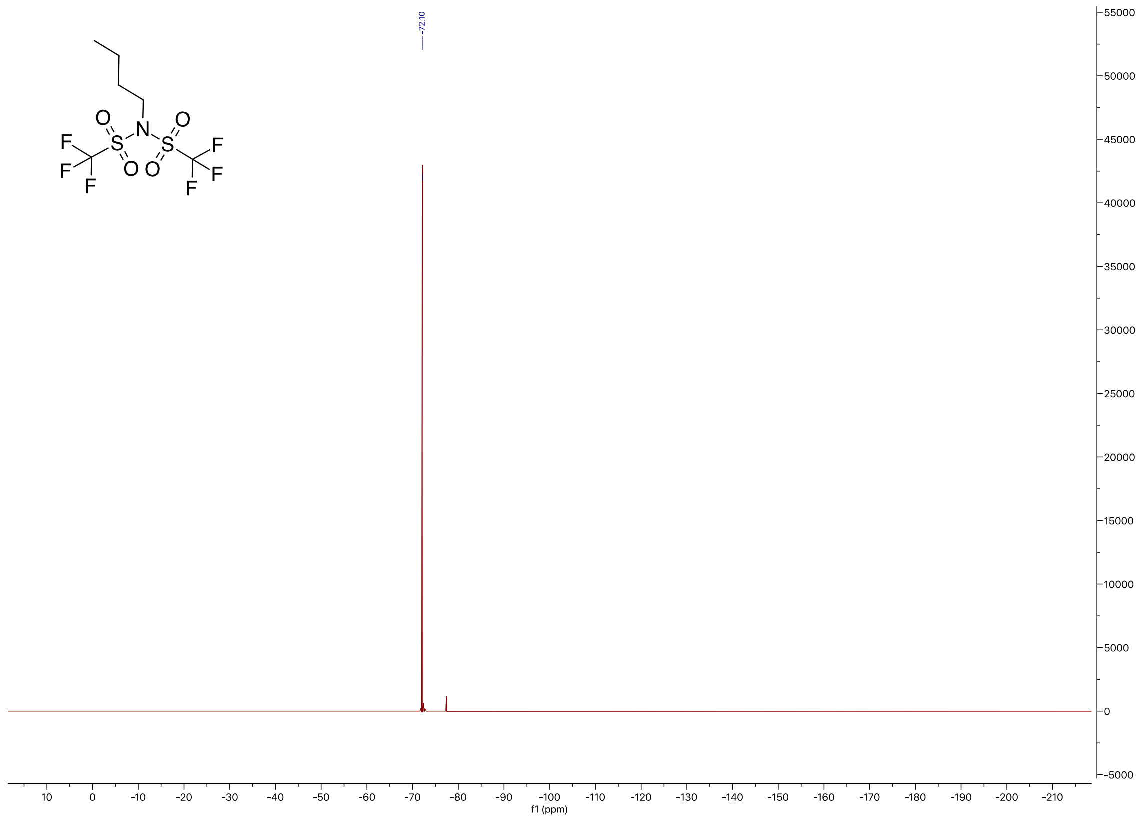

Figure S9. ^{19}F-NMR spectrum of **1a**

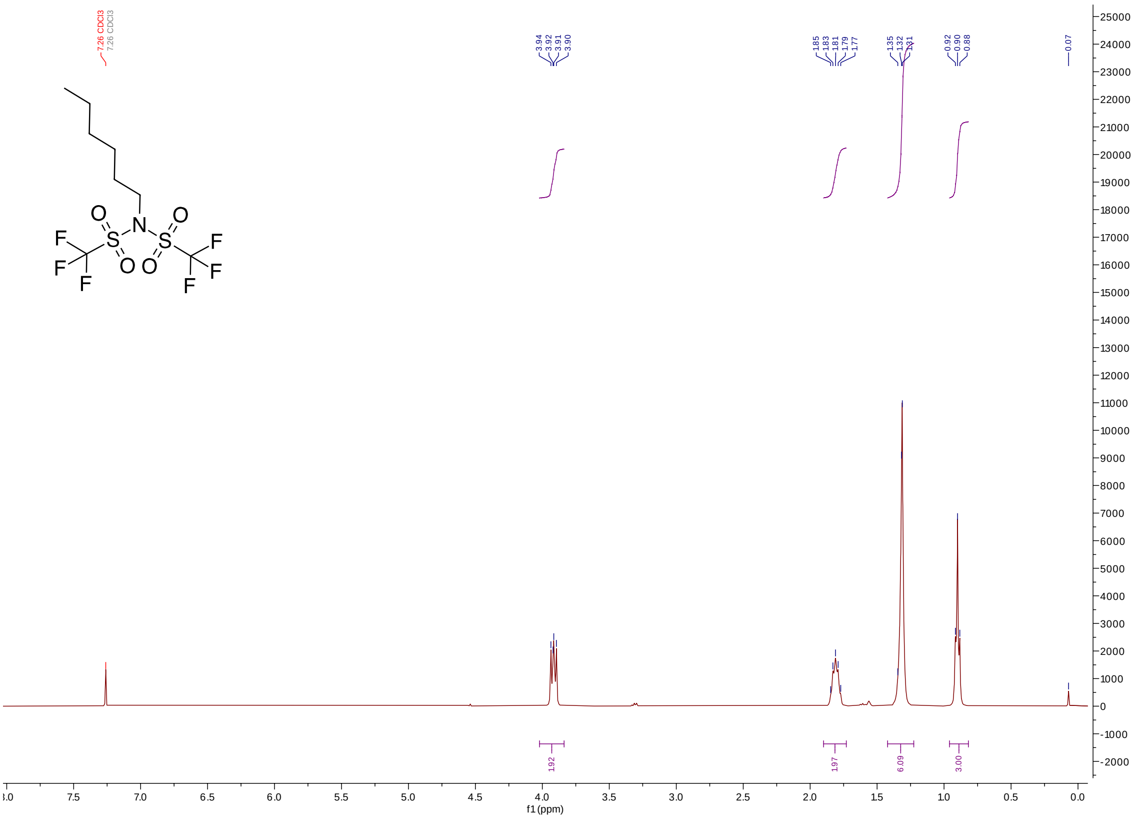

Figure S10. ^{1}H-NMR spectrum of **1b**

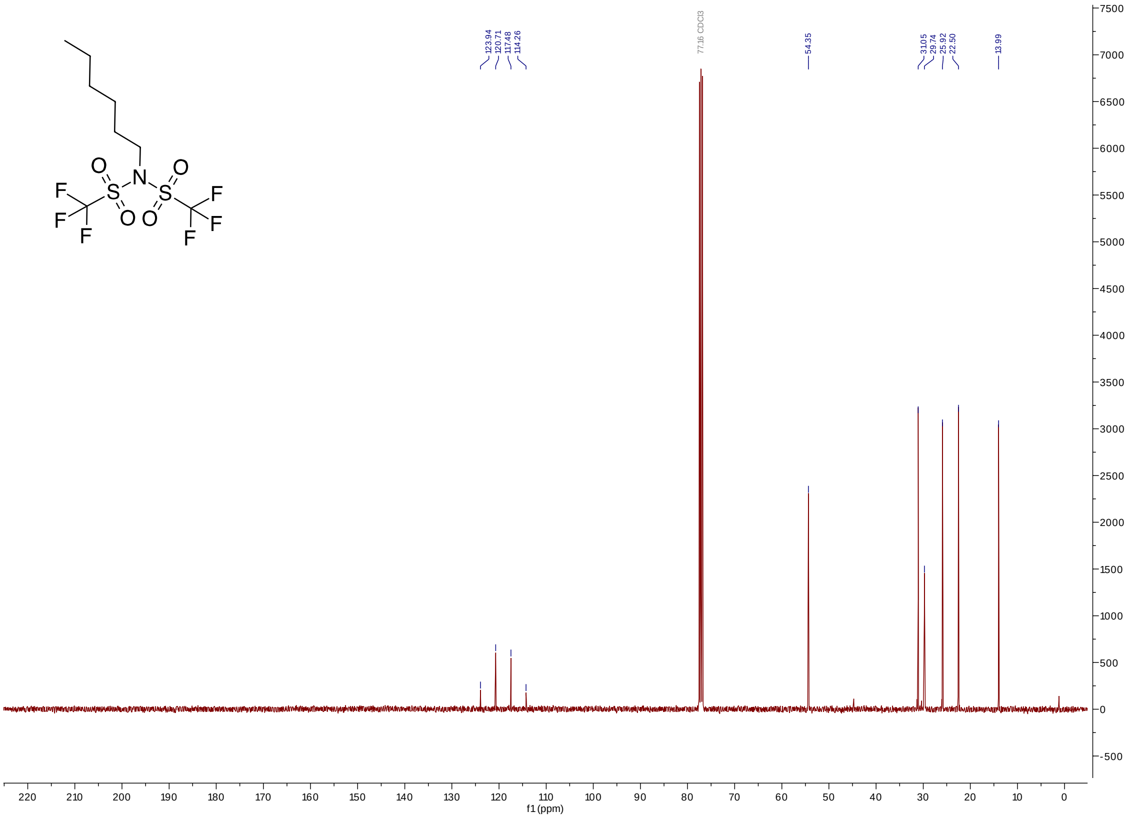

Figure S11. ^{13}C-NMR spectrum of **1b**

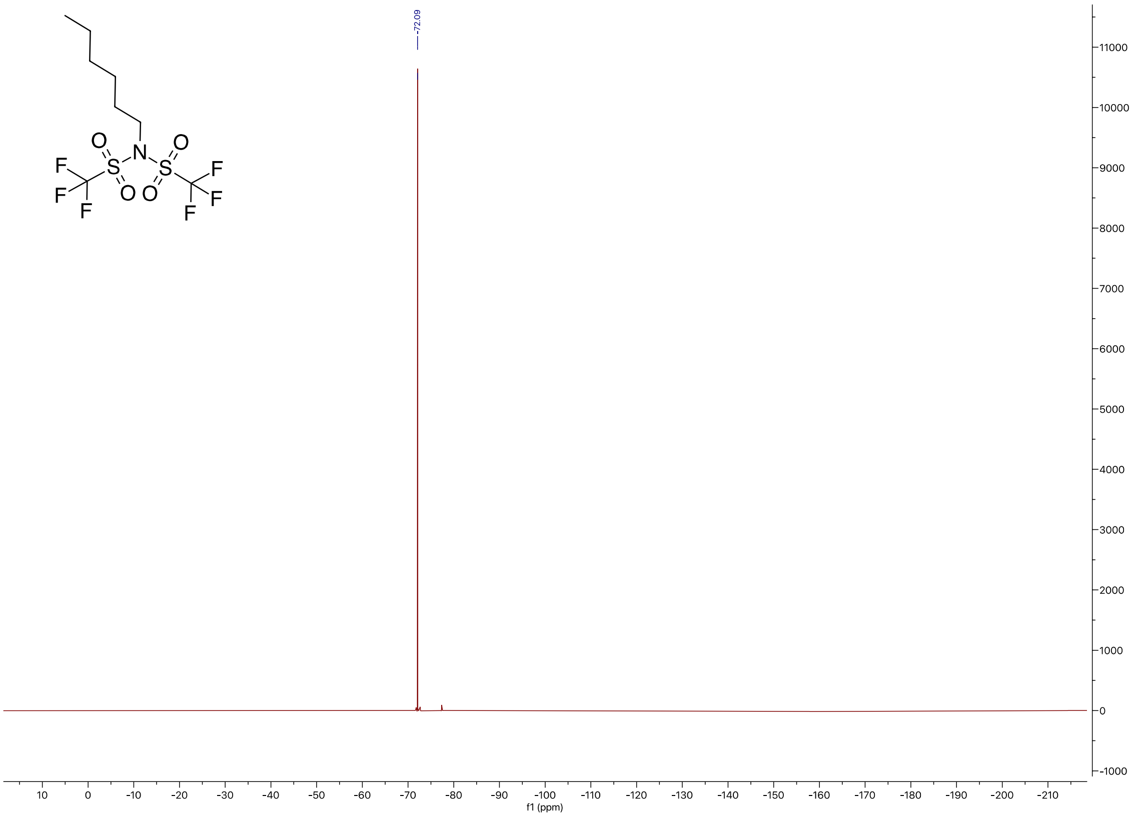

Figure S12. ^{19}F-NMR spectrum of **1b**

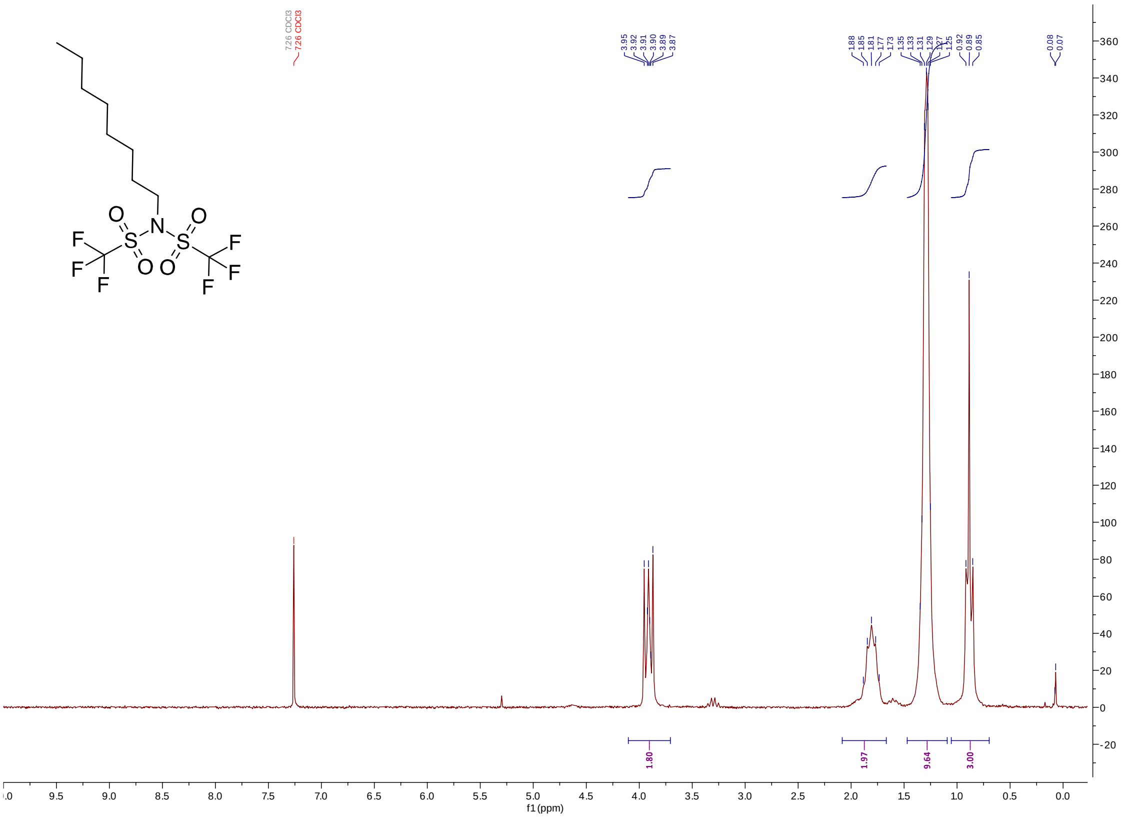

Figure S13. ^{1}H-NMR spectrum of **1c**

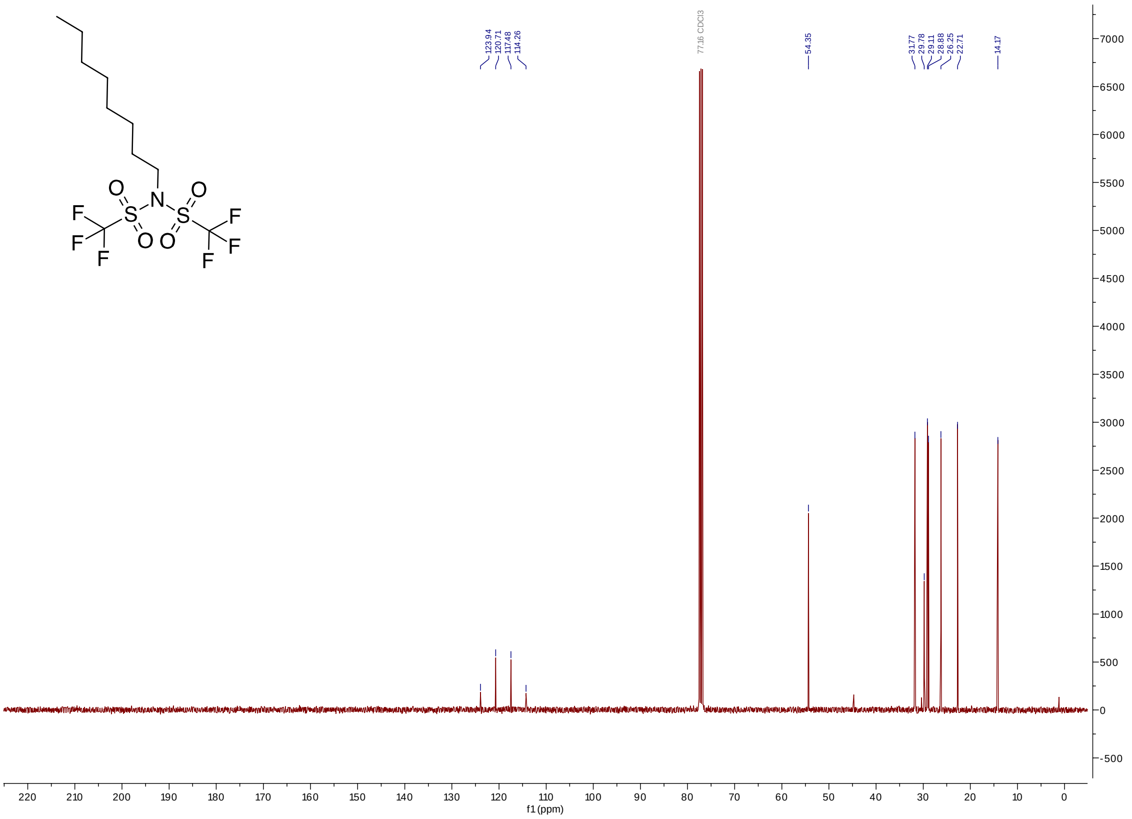

Figure S14. ^{13}C-NMR spectrum of **1c**

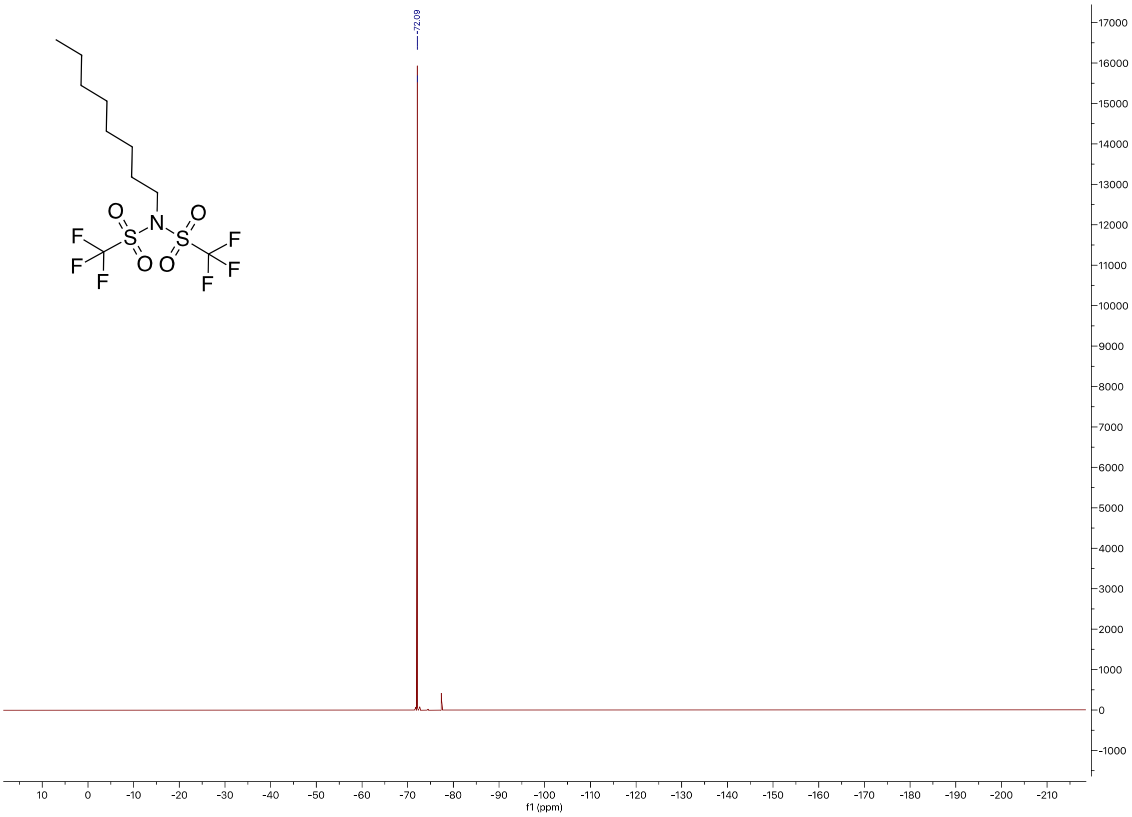

Figure S15. ^{19}F-NMR spectrum of **1c**

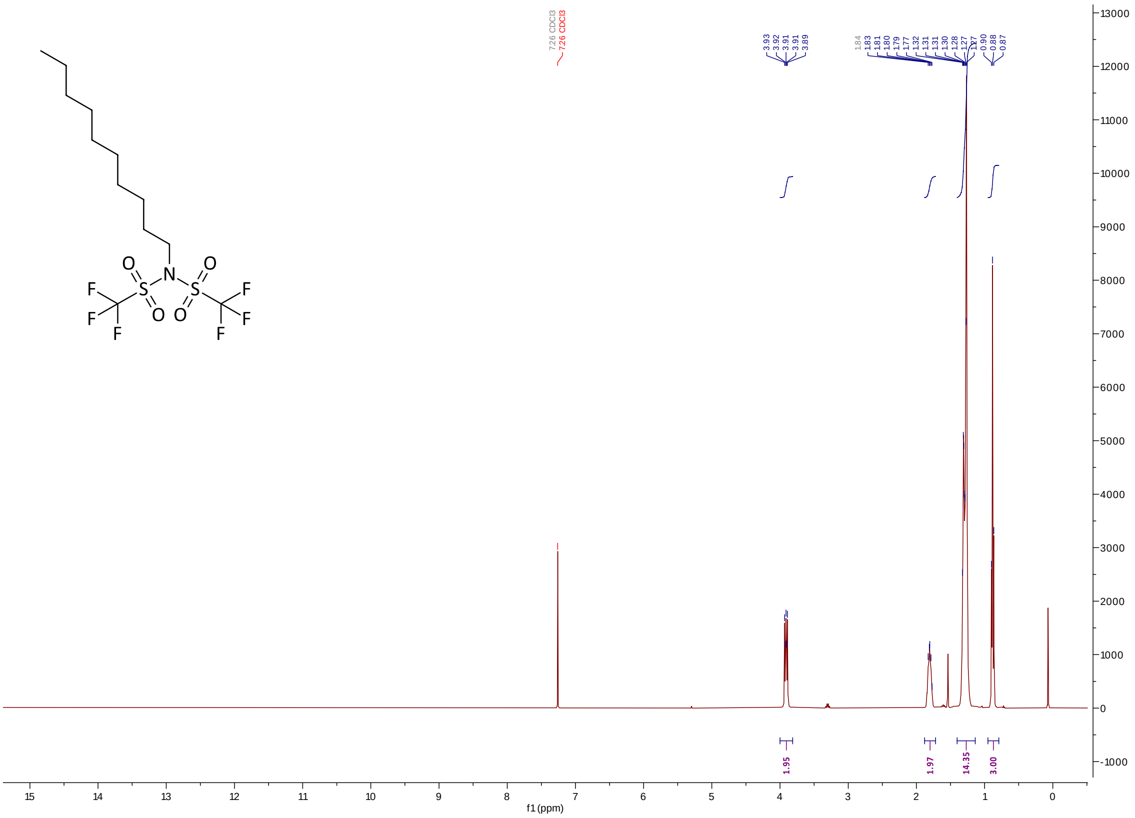

Figure S16. ^{1}H-NMR spectrum of **1d**

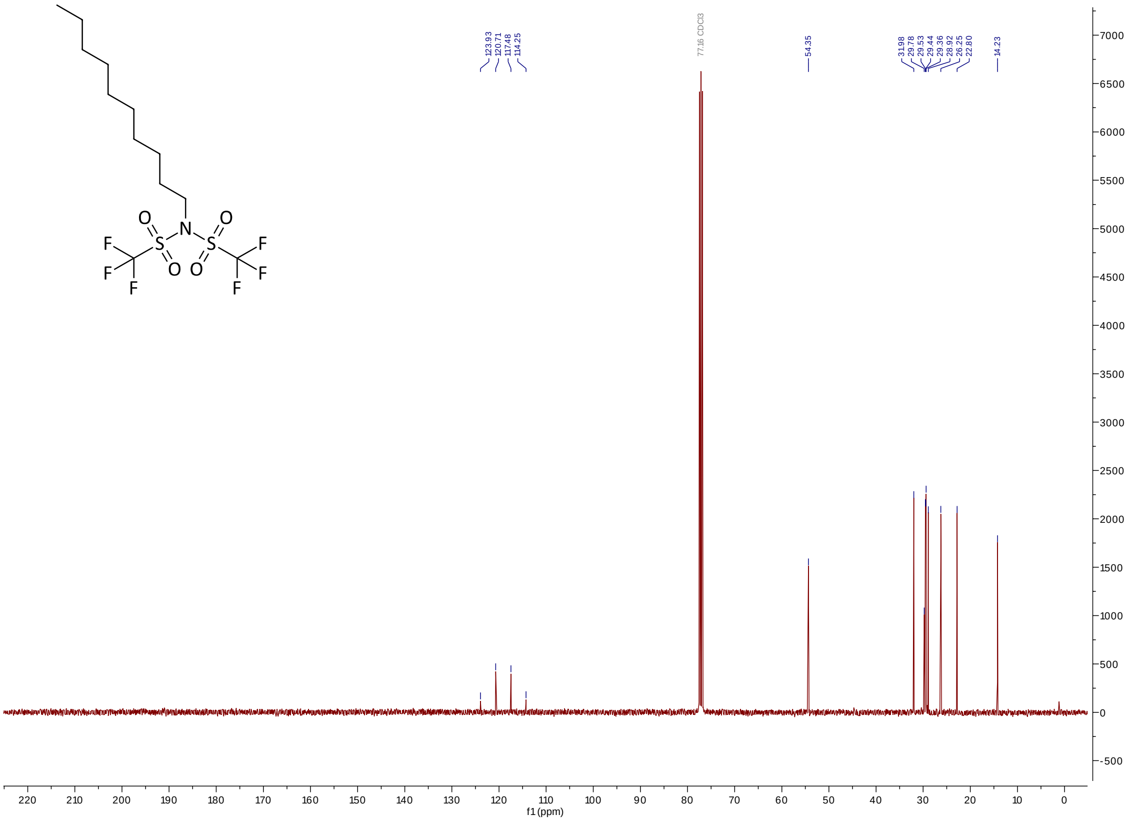

Figure S17. ^{13}C-NMR spectrum of **1d**

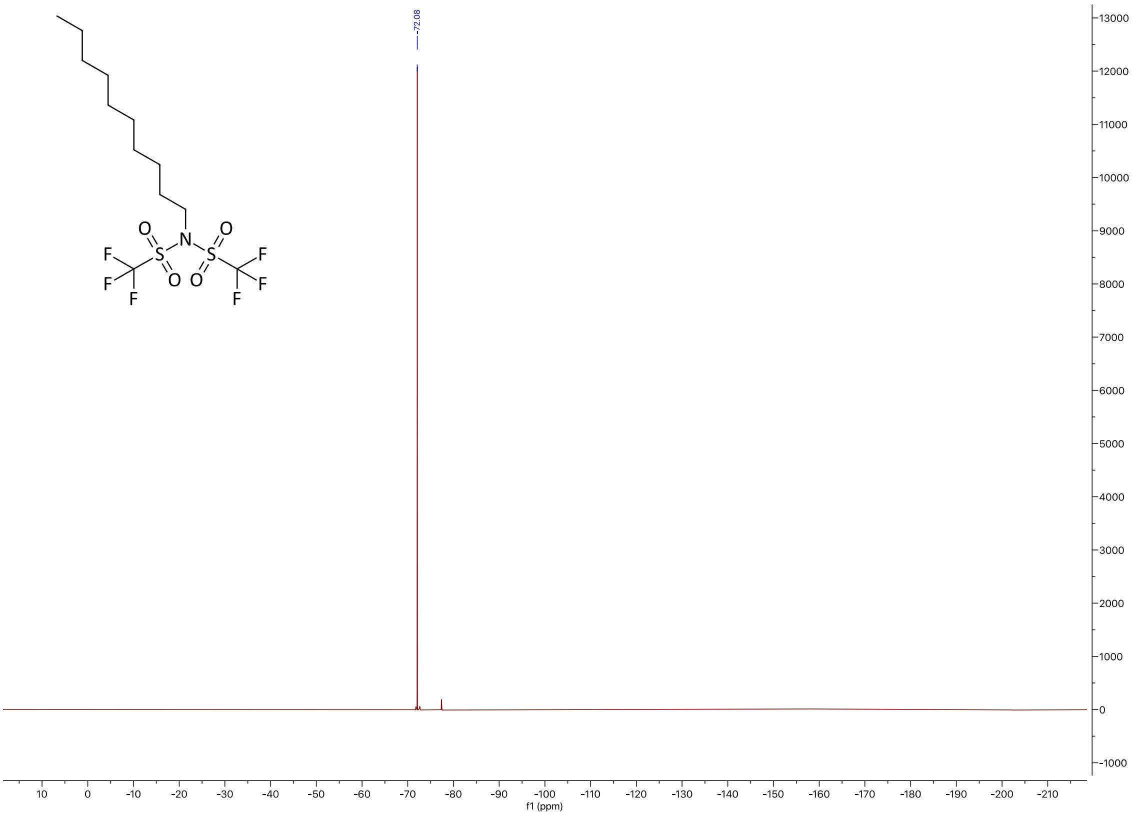

Figure S18. ^{19}F-NMR spectrum of **1d**

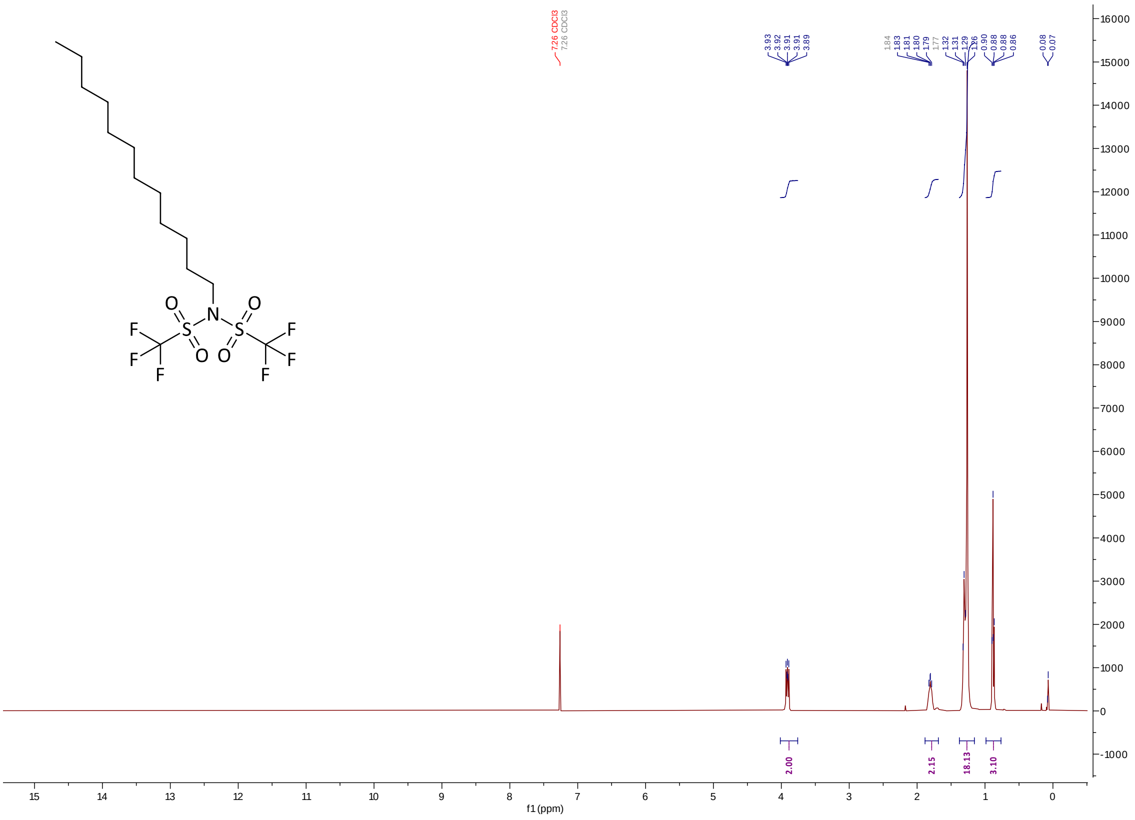

Figure S19. ^{1}H-NMR spectrum of **1e**

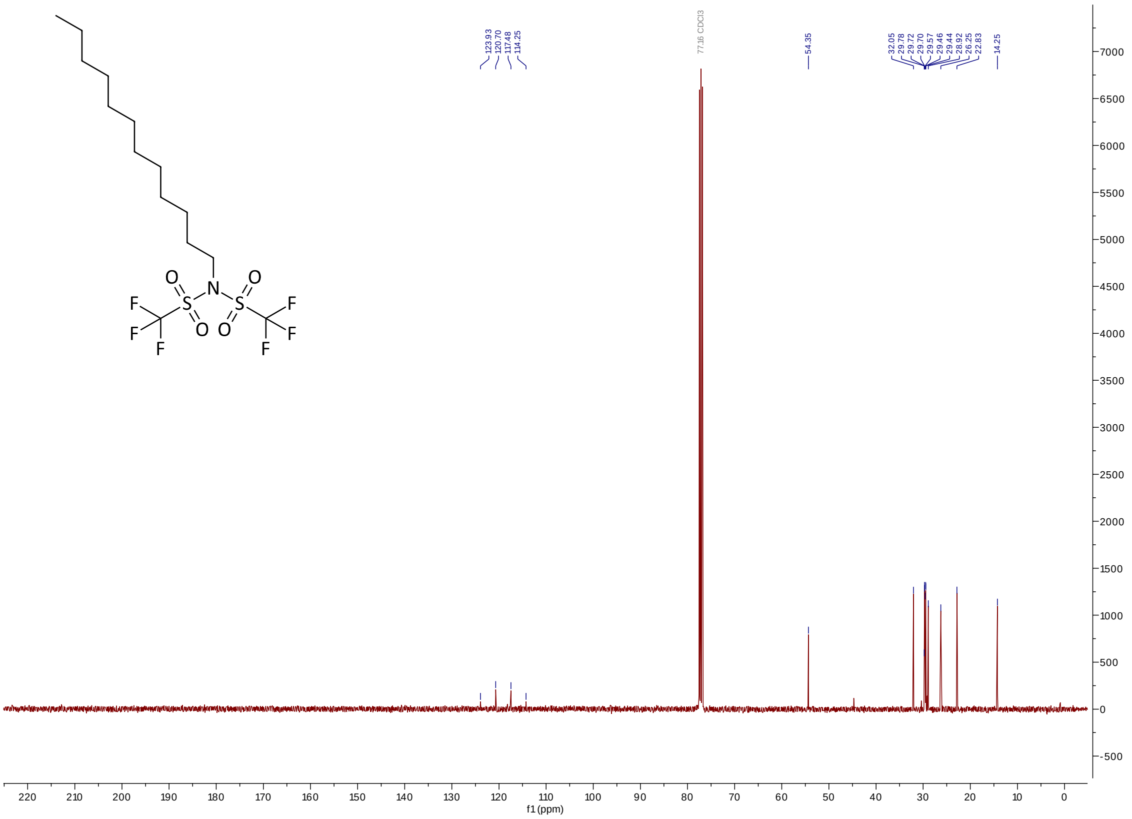

Figure S20. ^{13}C-NMR spectrum of **1e**

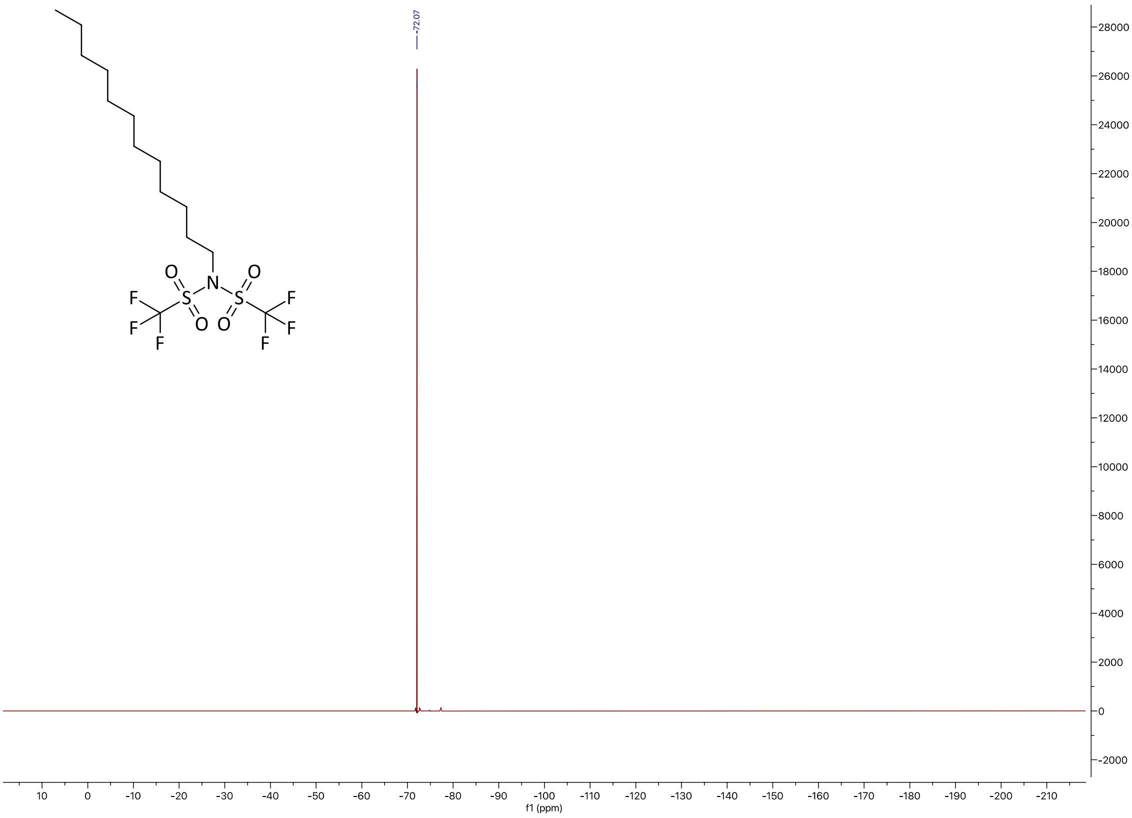

Figure S21. ^{19}F-NMR spectrum of **1e**

13. NMR spectra of ionic liquids

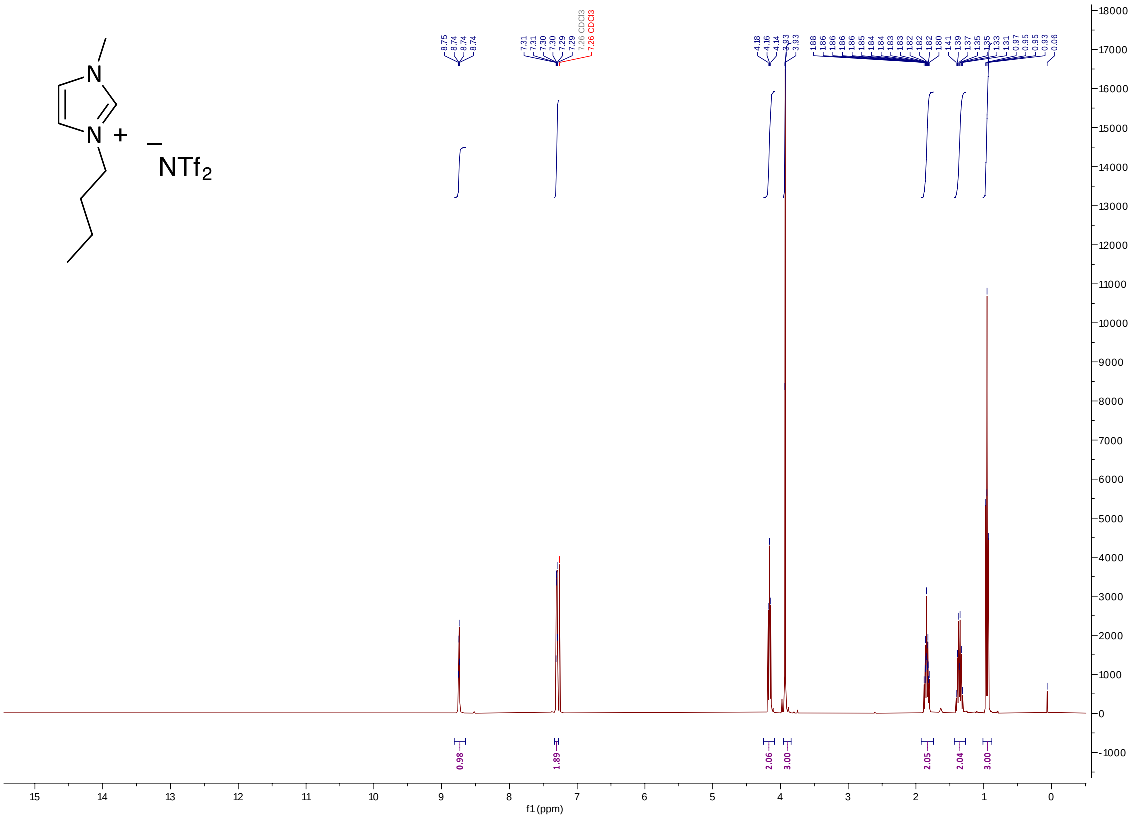

Figure S22. ^{1}H-NMR spectrum of **2a**

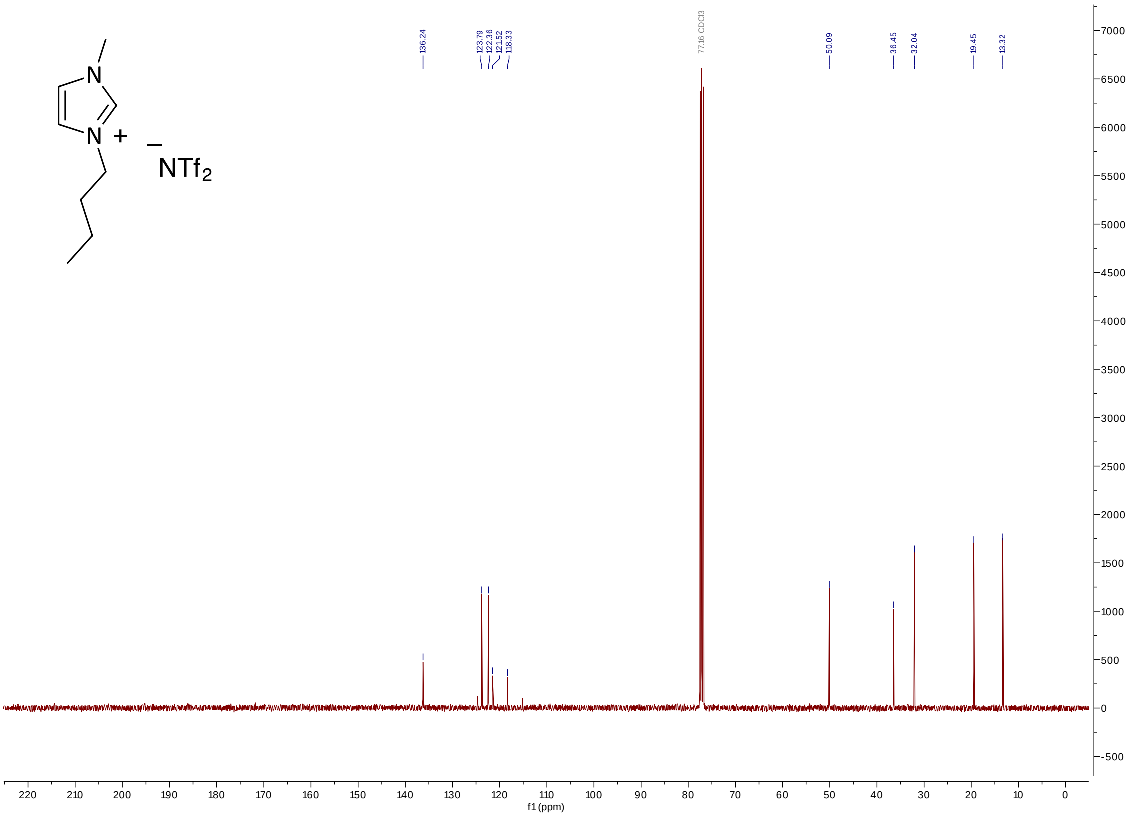

Figure S23. ^{13}C-NMR spectrum of **2a**

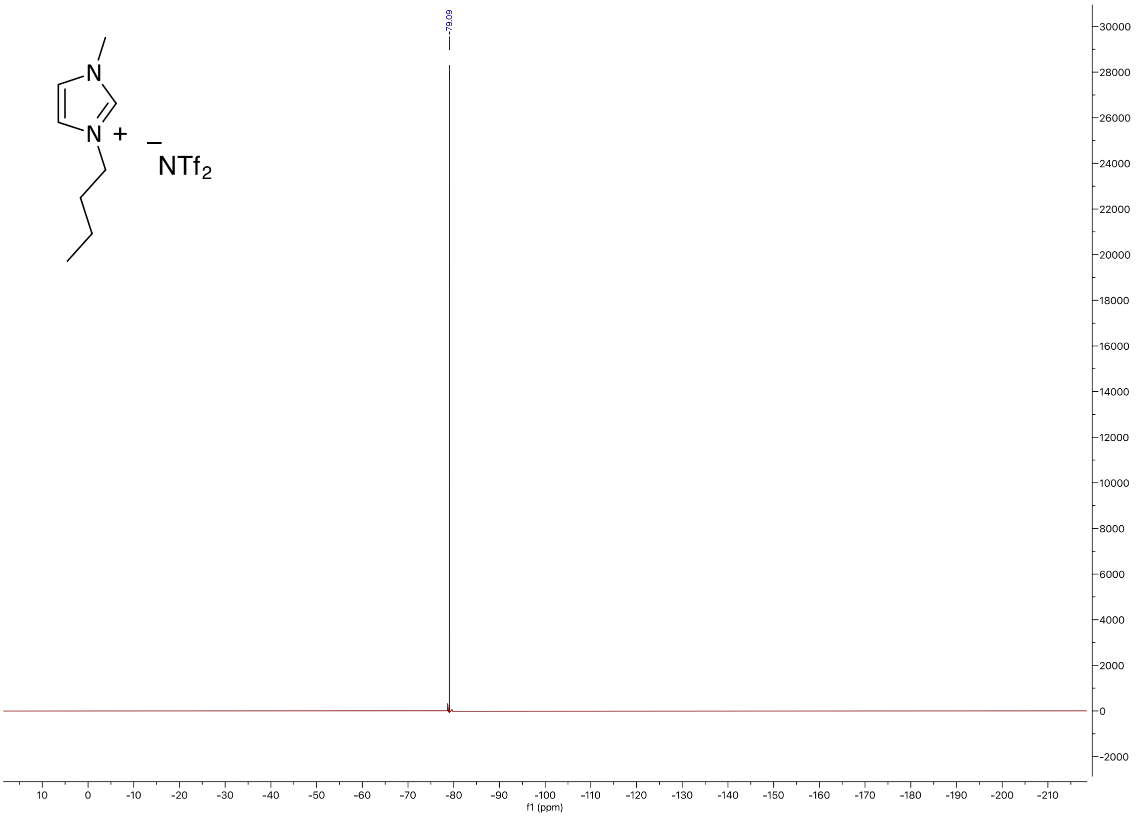

Figure S24. ^{19}F-NMR spectrum of **2a**

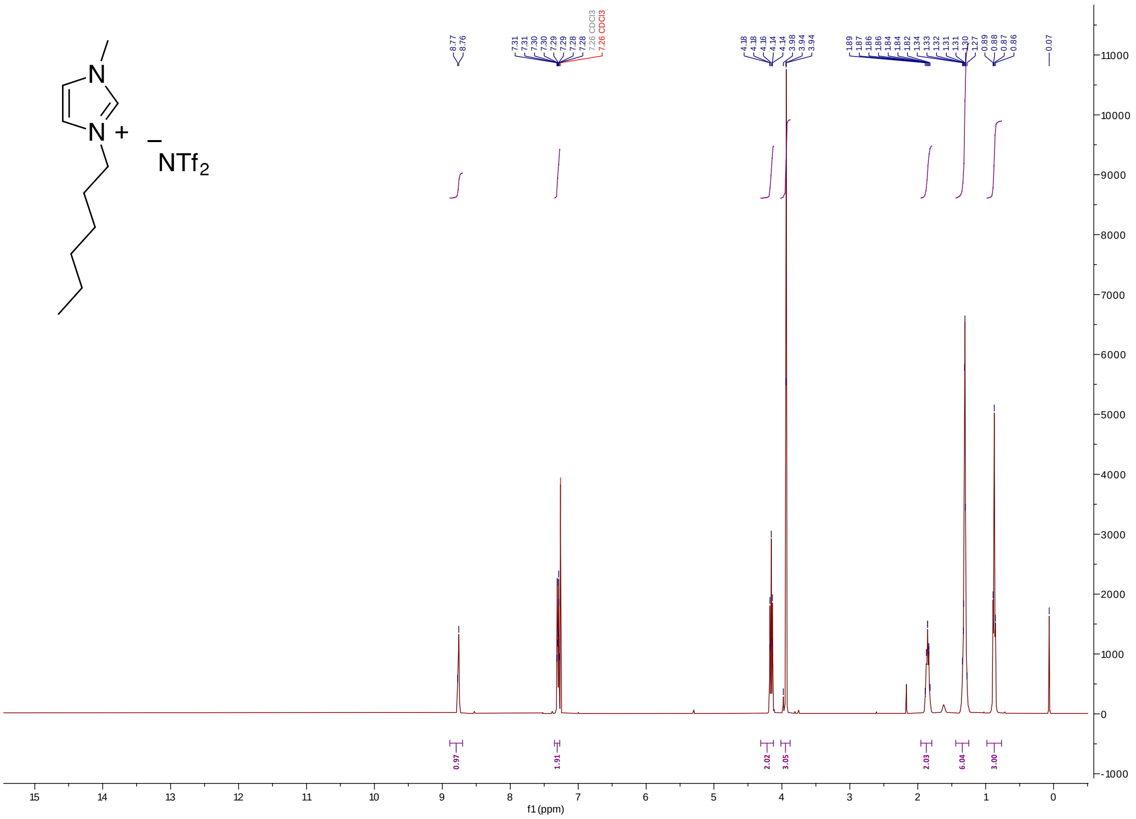

Figure S25. ^{1}H-NMR spectrum of **2b**

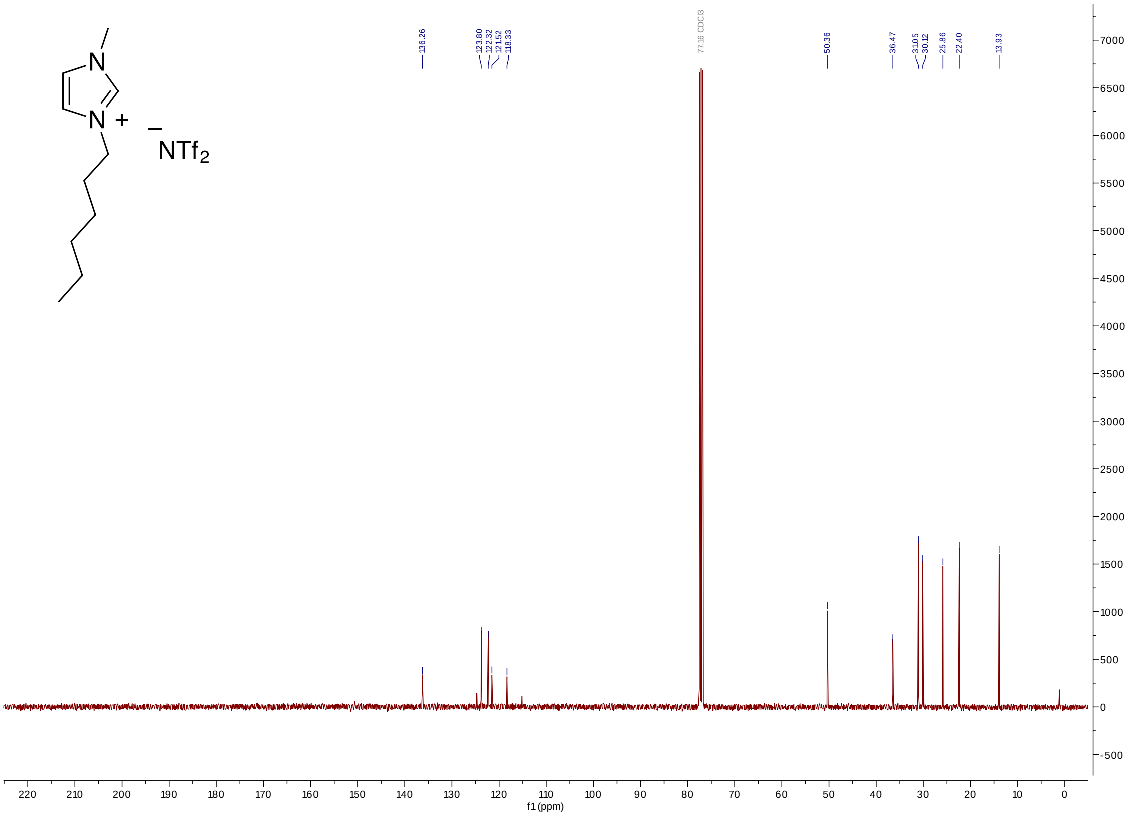

Figure S26. ^{13}C-NMR spectrum of **2b**

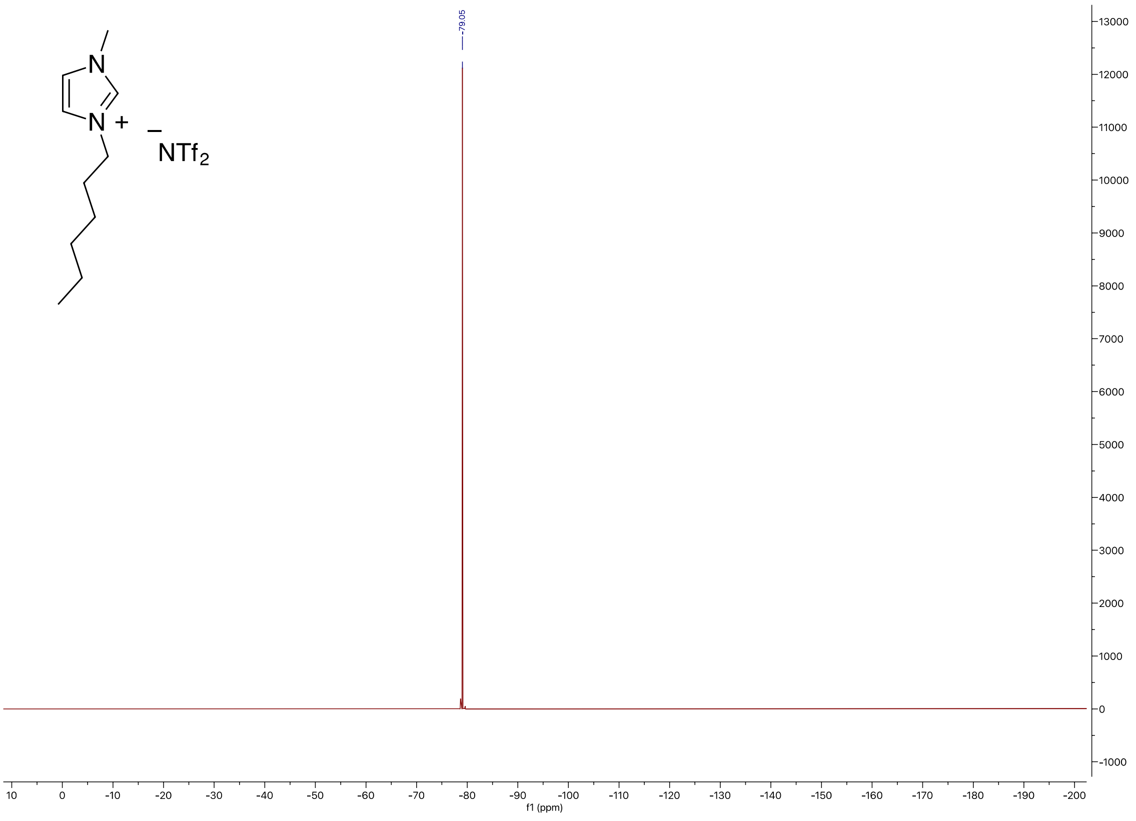

Figure S27. ^{19}F-NMR spectrum of **2b**

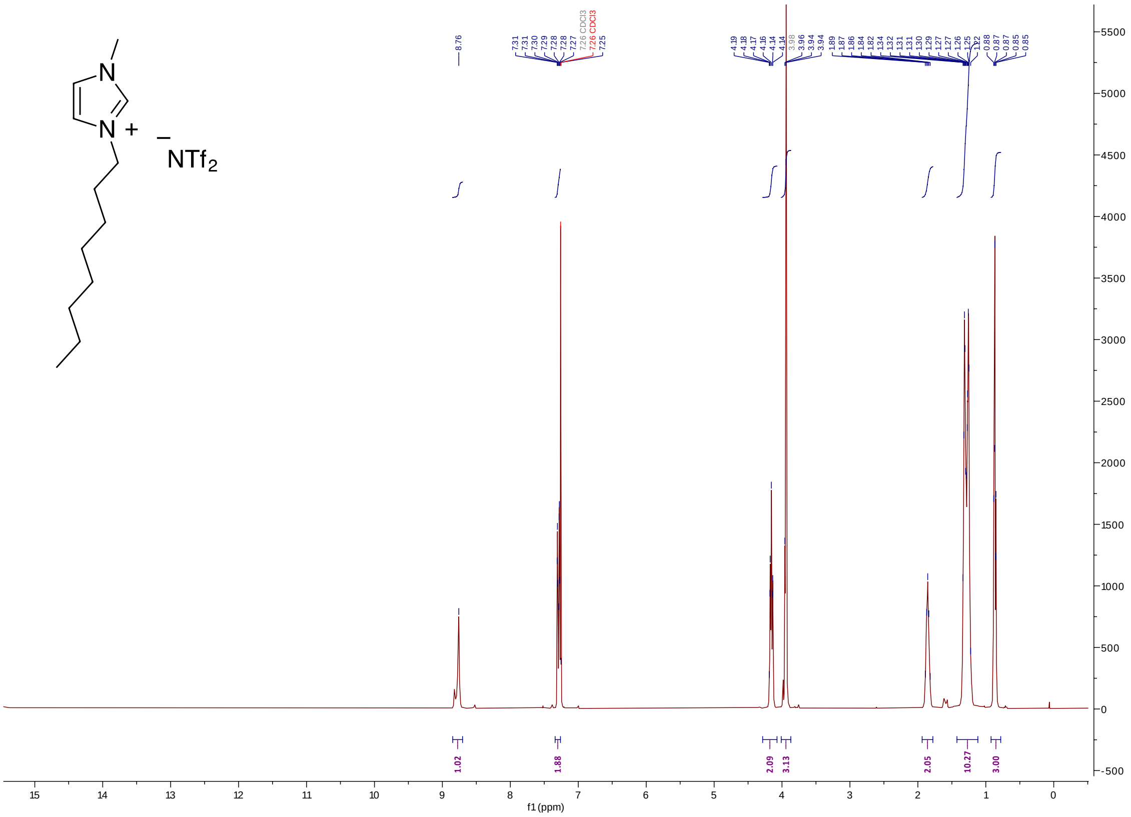

Figure S28. ^{1}H-NMR spectrum of **2c**

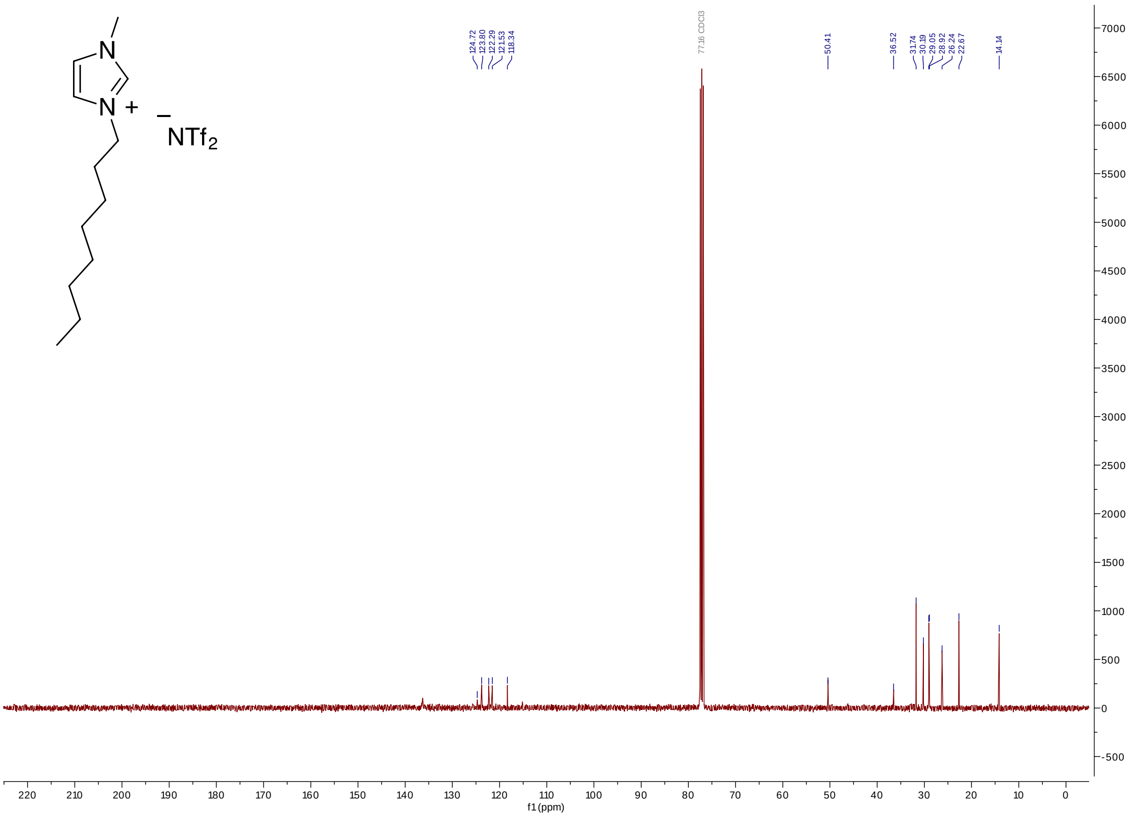

Figure S29. ^{13}C-NMR spectrum of **2c**

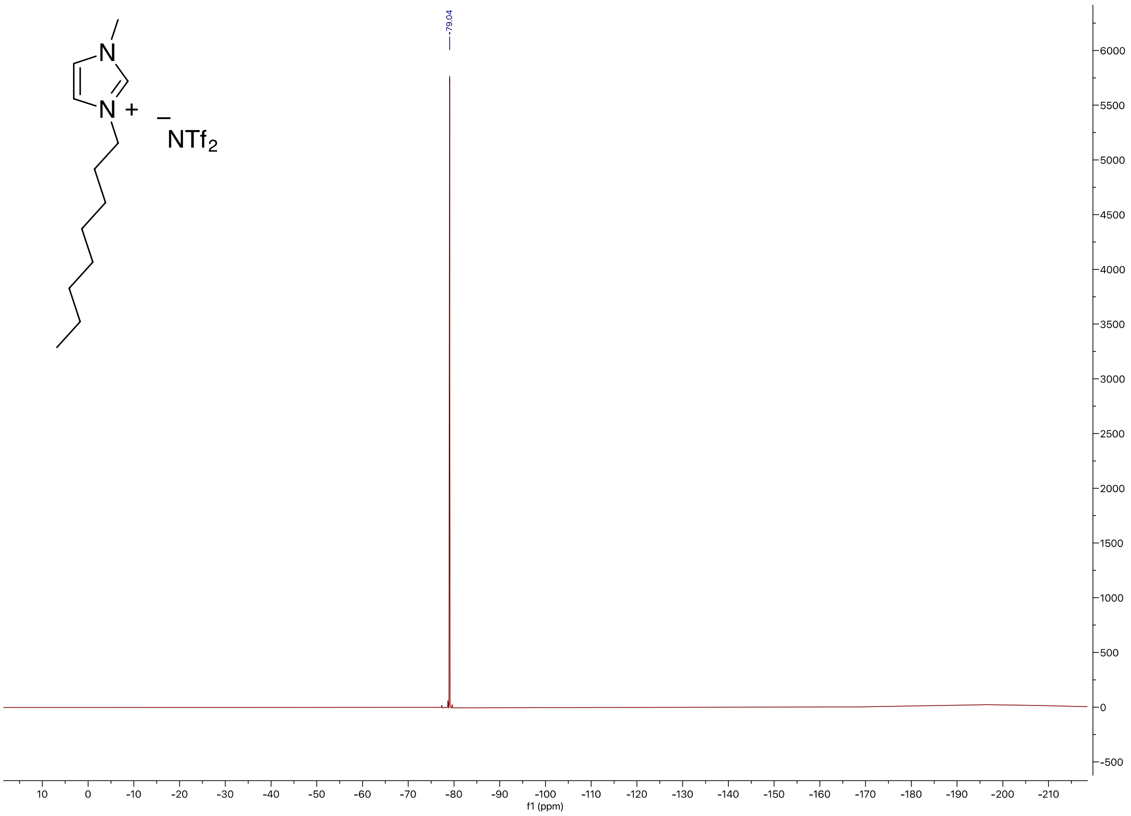

Figure S30. ^{19}F-NMR spectrum of **2c**

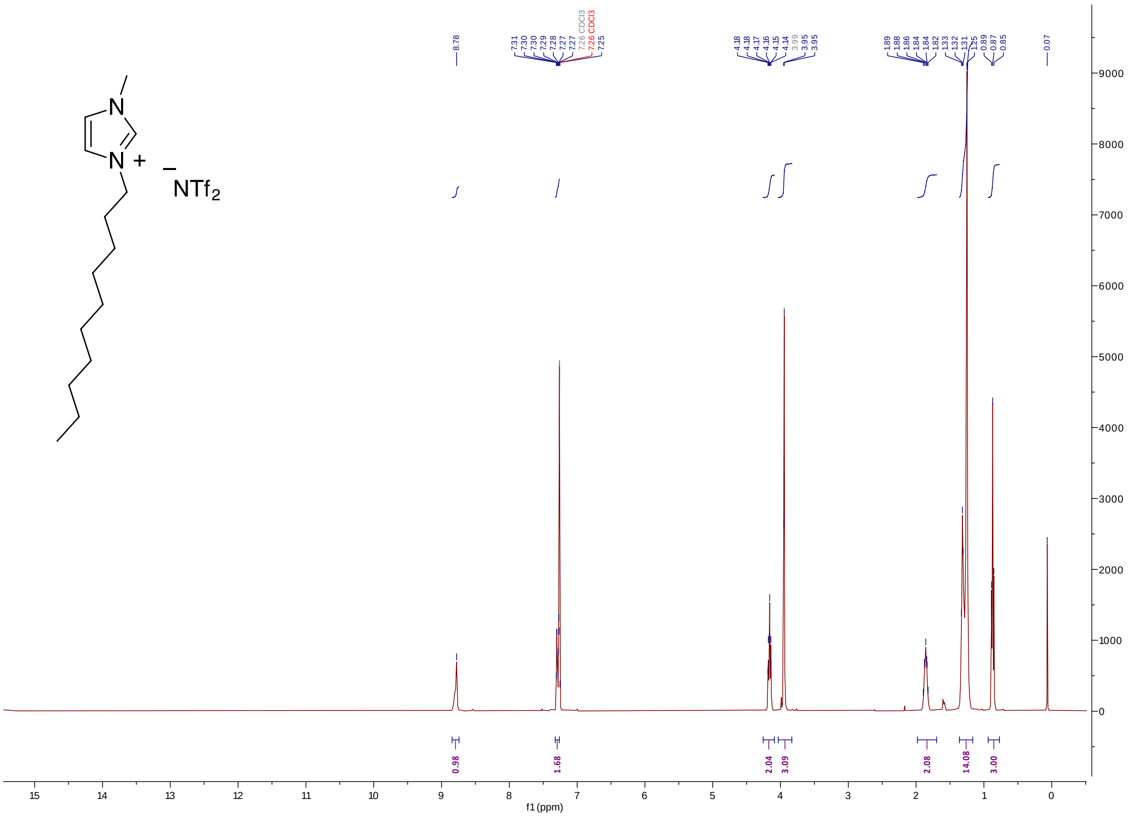

Figure S31. ^{1}H-NMR spectrum of **2d**

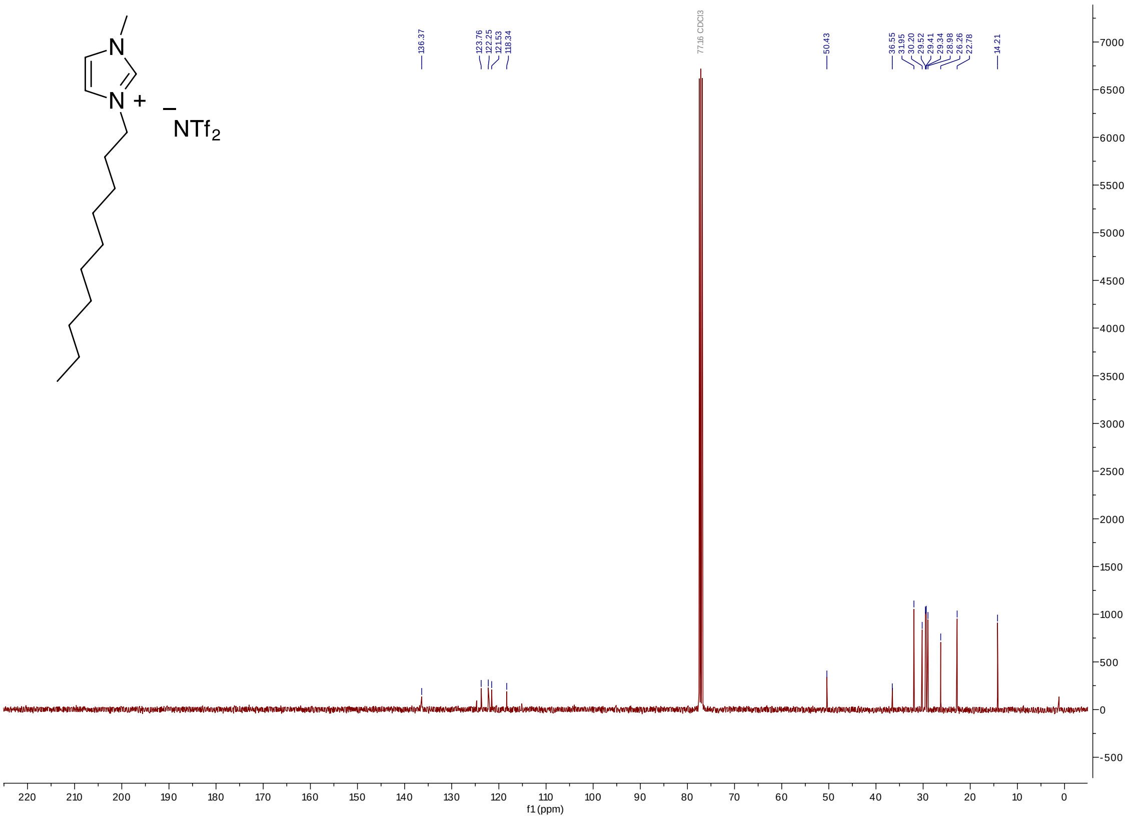

Figure S32. ^{13}C-NMR spectrum of **2d**

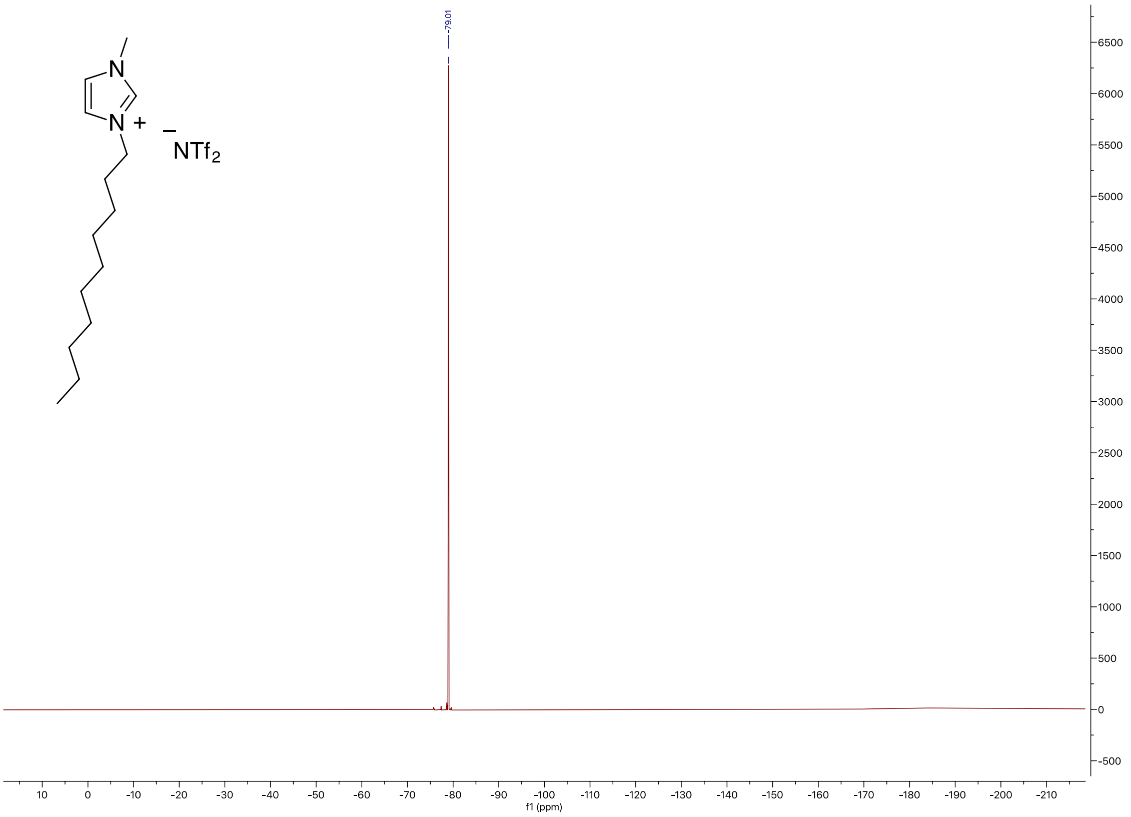

Figure S33. ^{19}F-NMR spectrum of **2d**

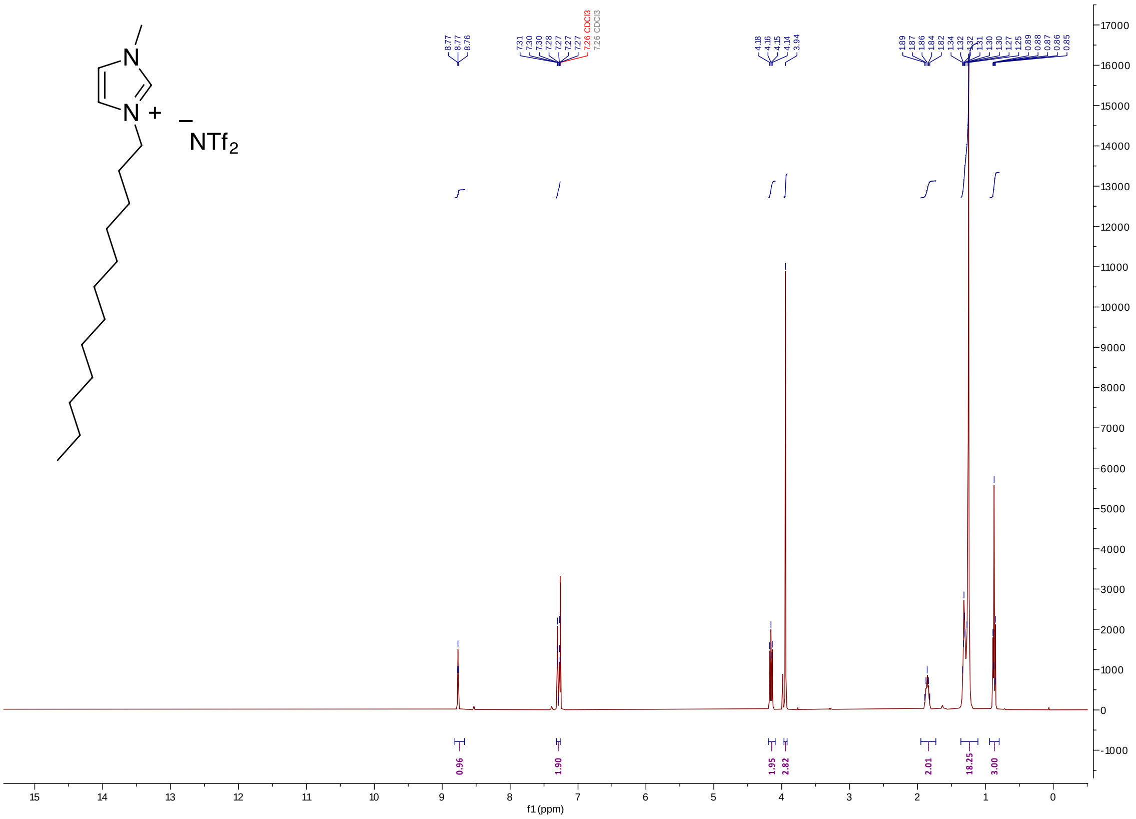

Figure S34. ^{1}H-NMR spectrum of **2e**

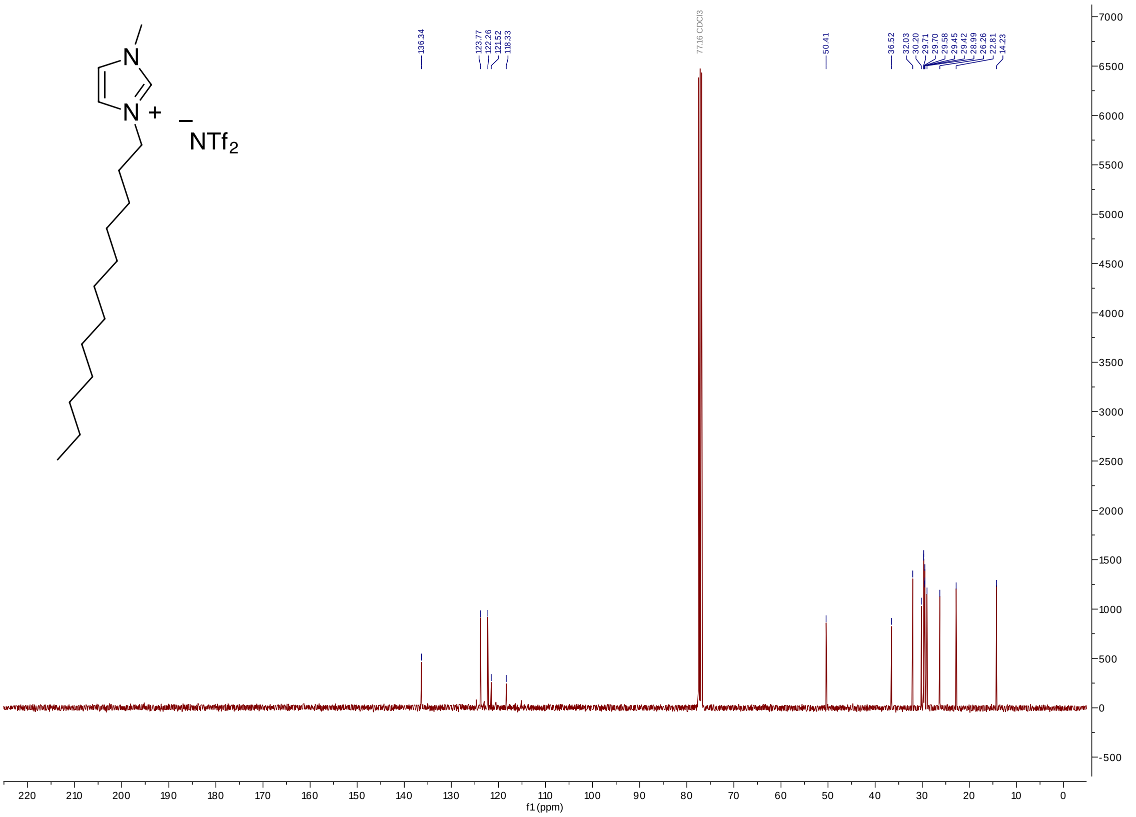

Figure S35. ^{13}C-NMR spectrum of **2e**

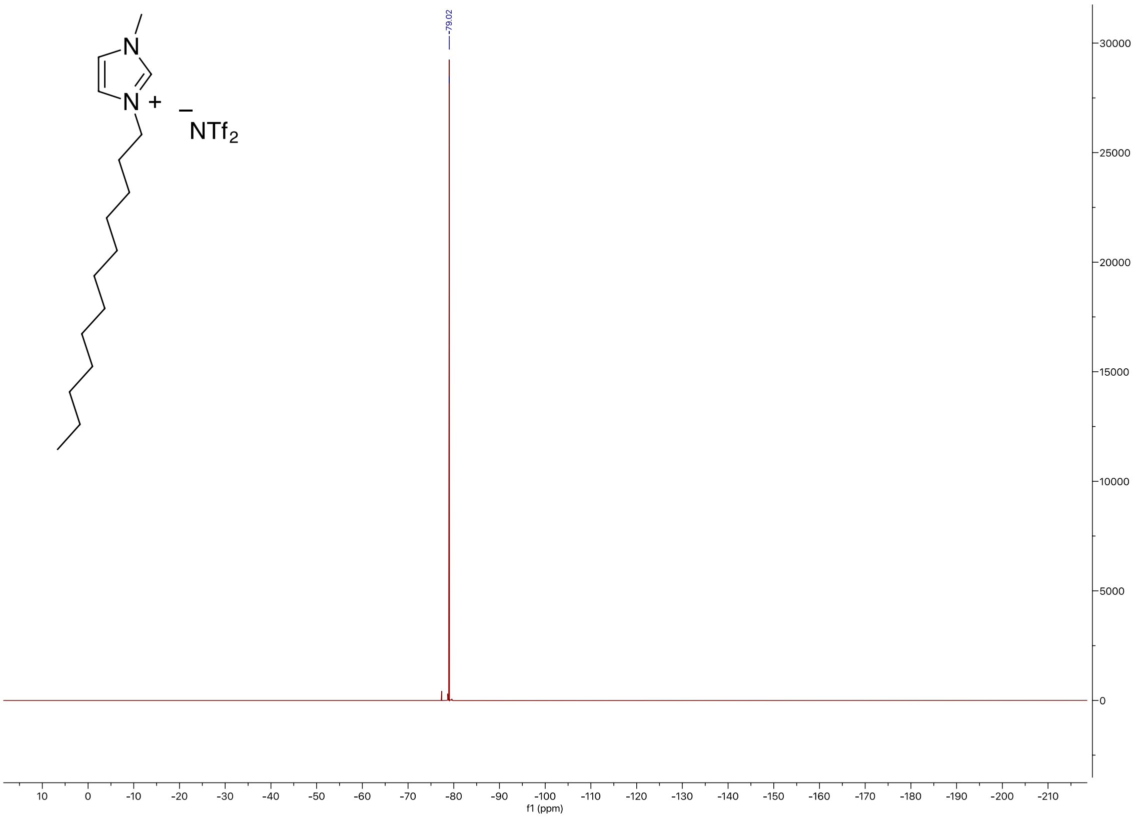

Figure S36. ^{19}F-NMR spectrum of **2e**

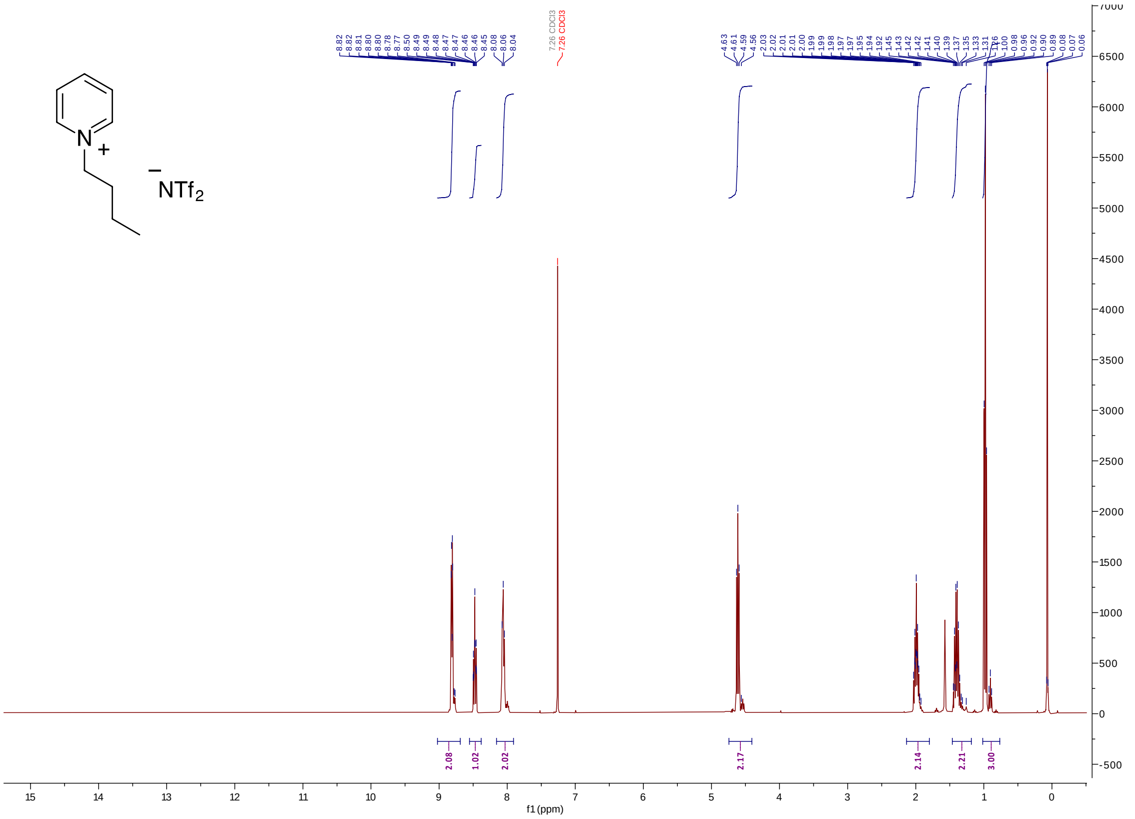

Figure S37. ^{1}H-NMR spectrum of **3a**

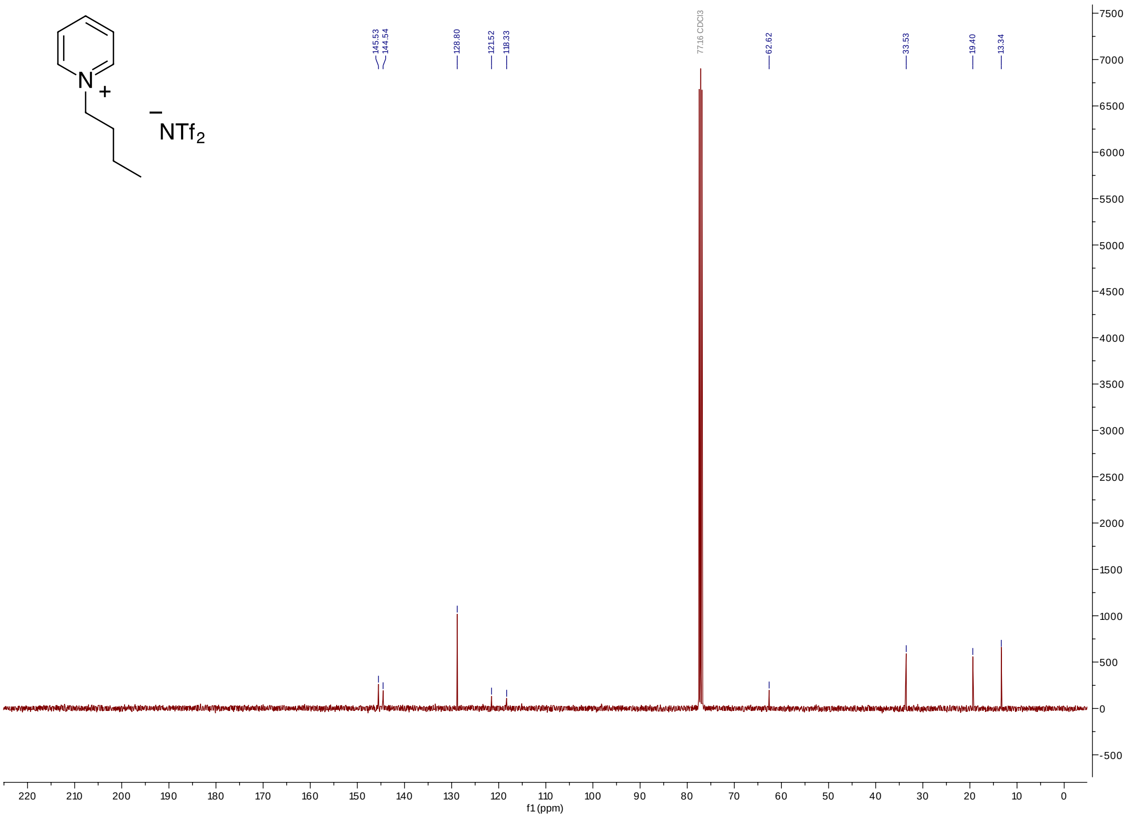

Figure S38. ^{13}C-NMR spectrum of **3a**

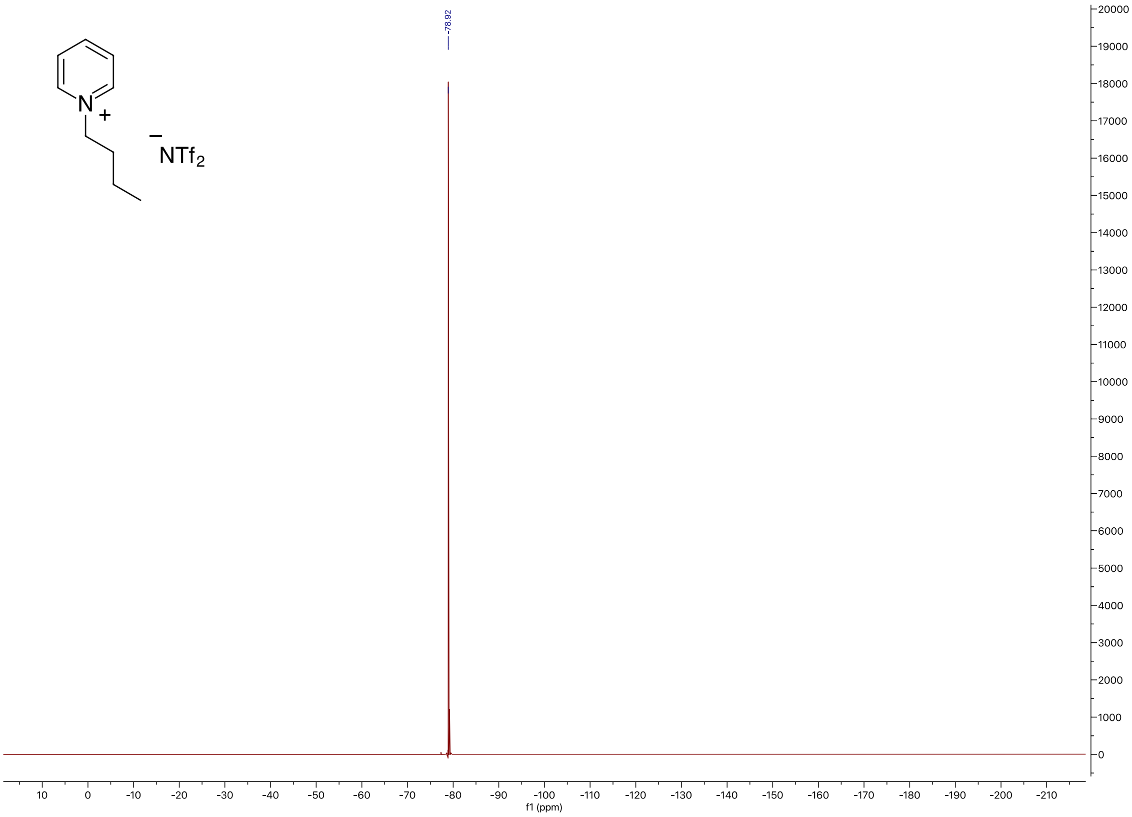

Figure S39. ^{19}F-NMR spectrum of **3a**

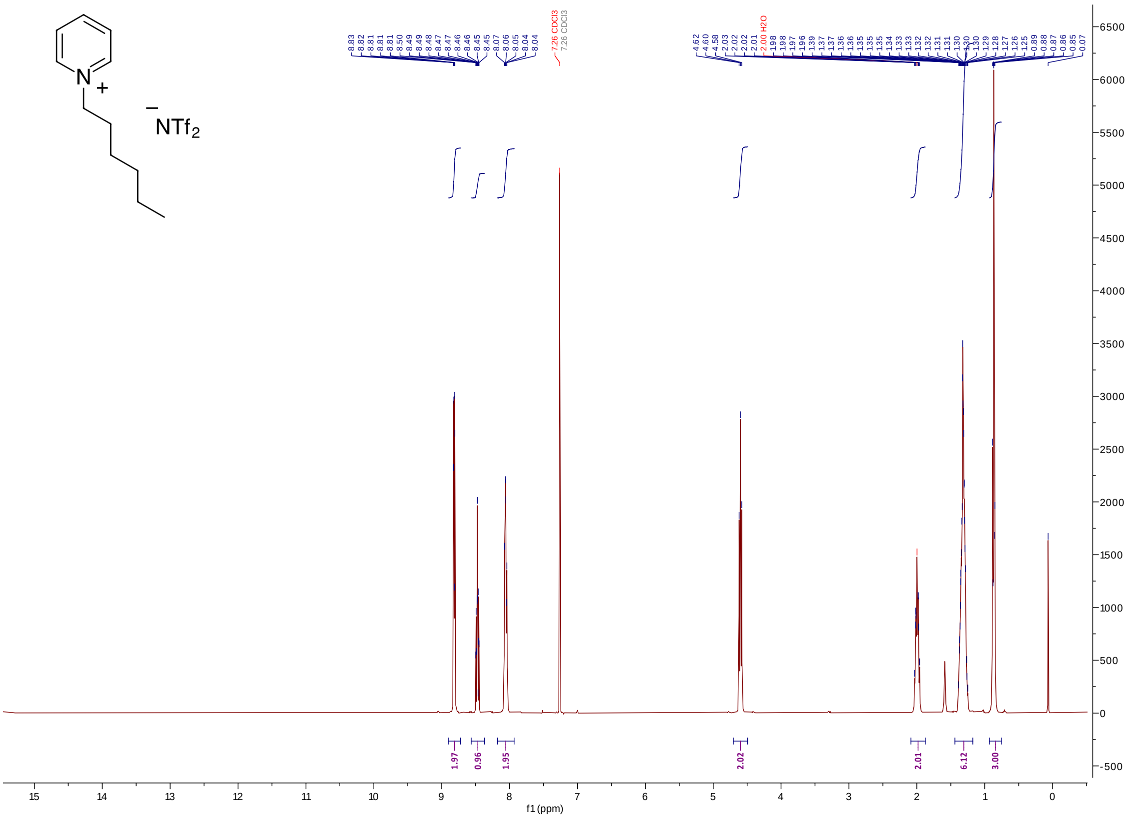

Figure S40. ^{1}H-NMR spectrum of **3b**

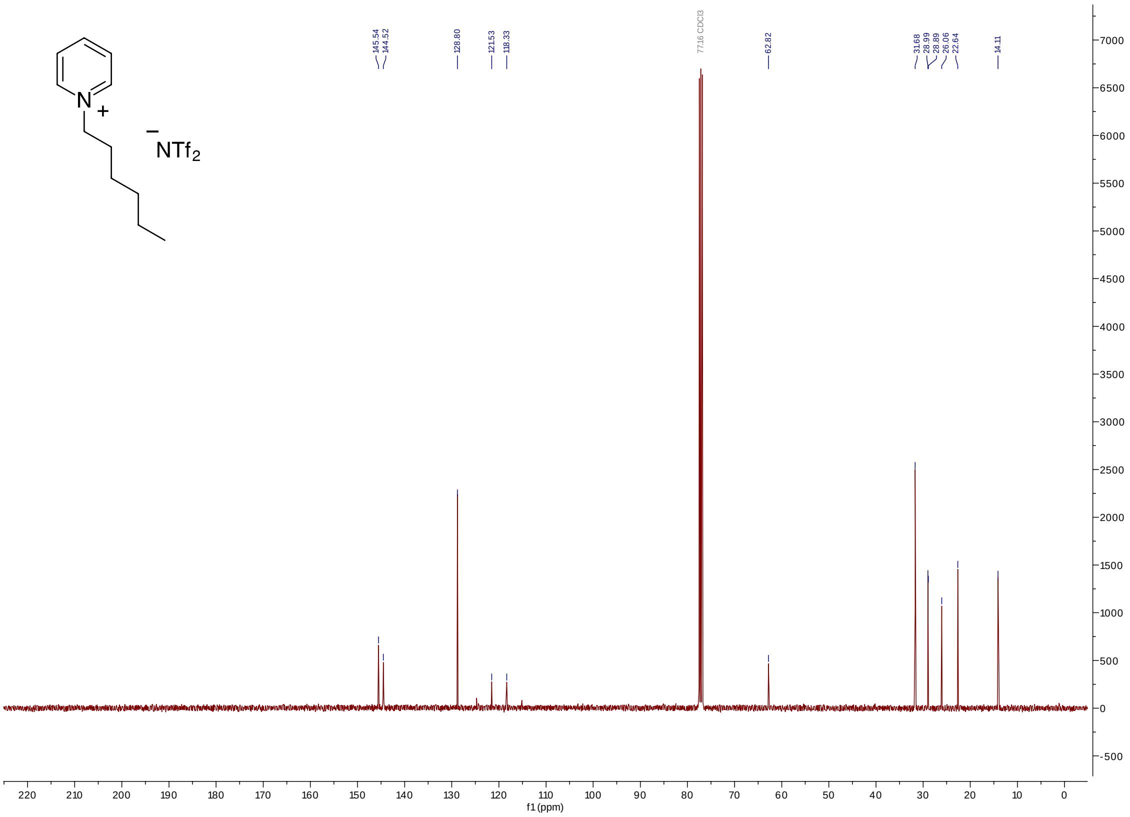

Figure S41. ^{13}C-NMR spectrum of **3b**

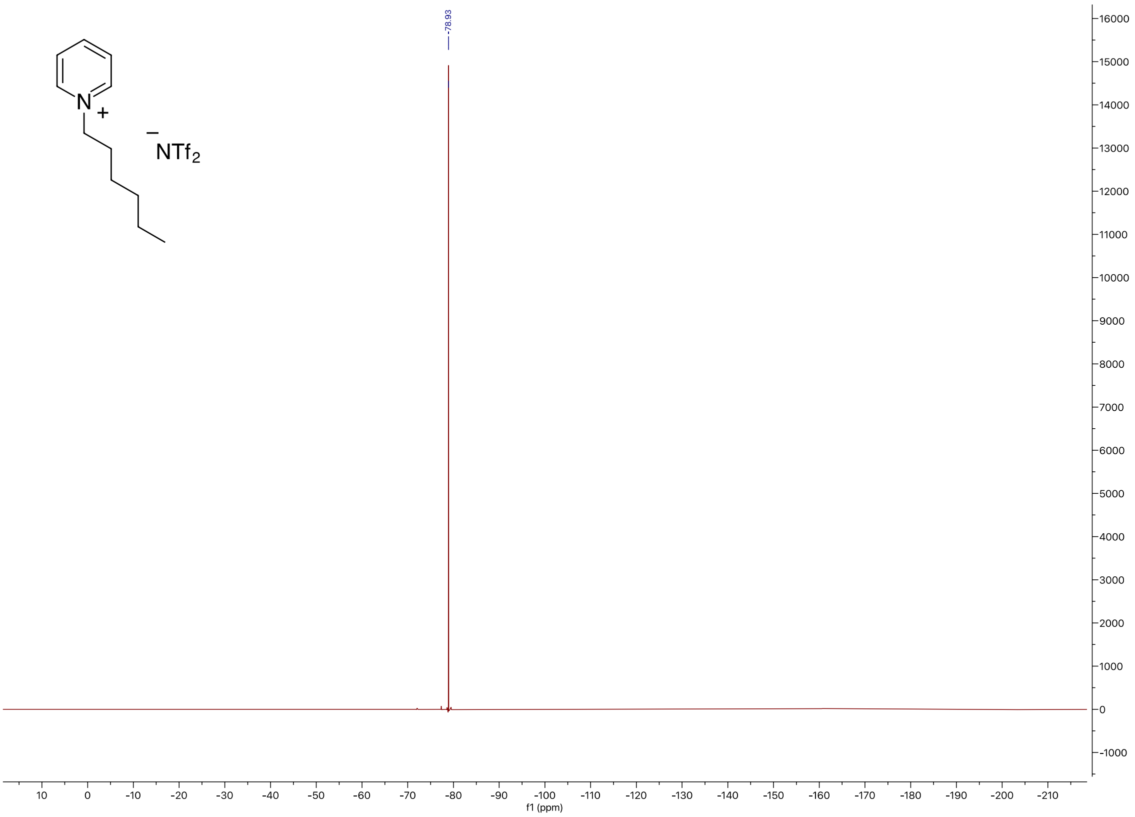

Figure S42. ^{19}F-NMR spectrum of **3b**

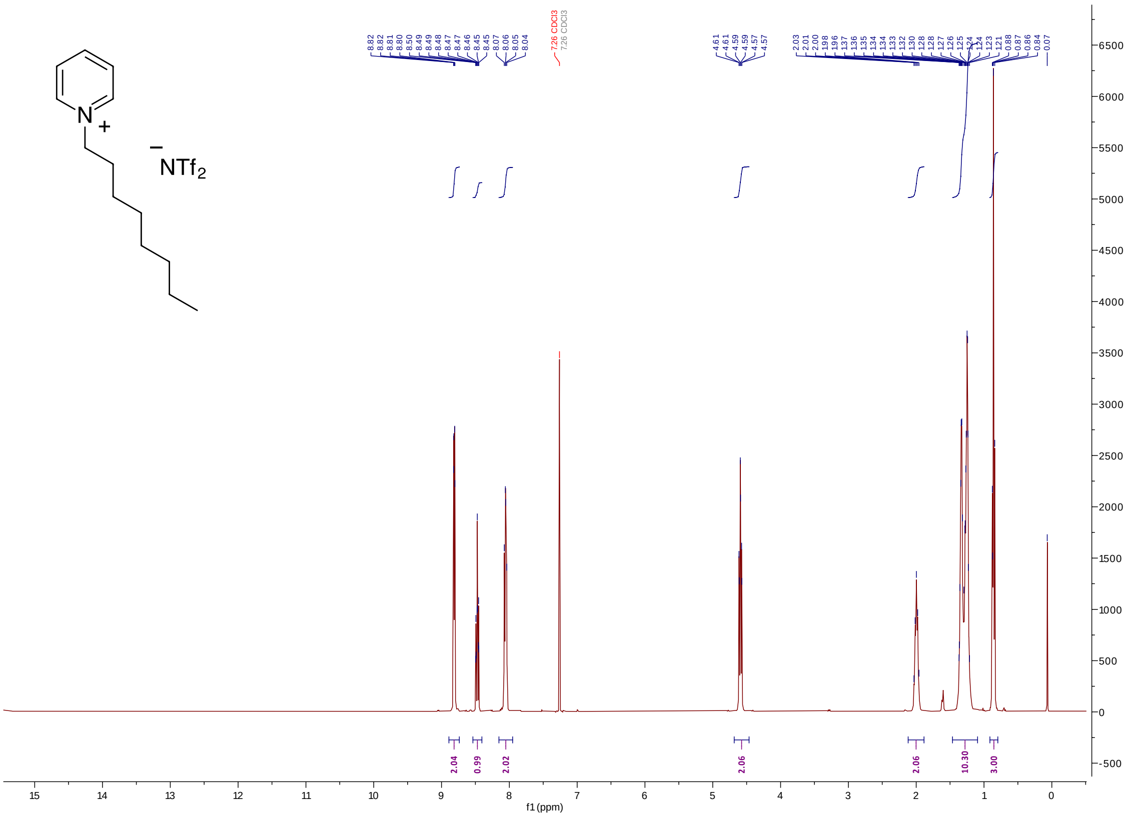

Figure S43. ^{1}H-NMR spectrum of **3c**

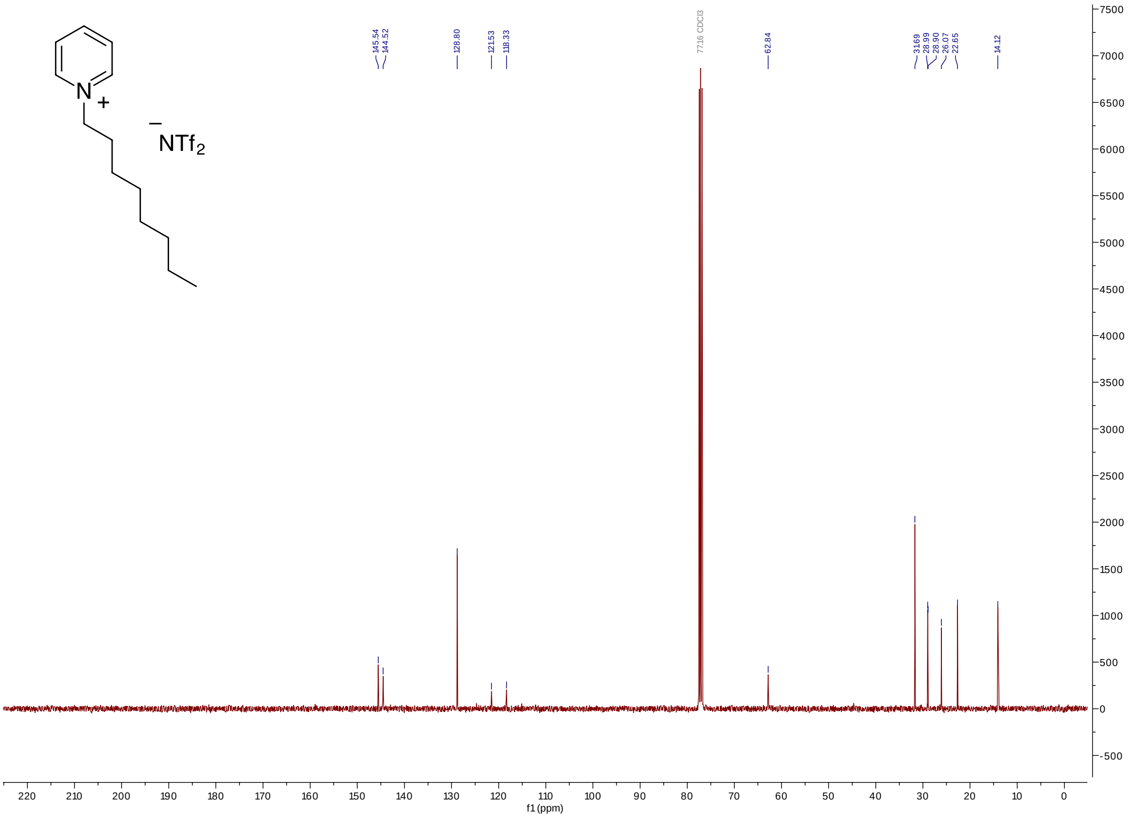

Figure S44. ^{13}C-NMR spectrum of **3c**

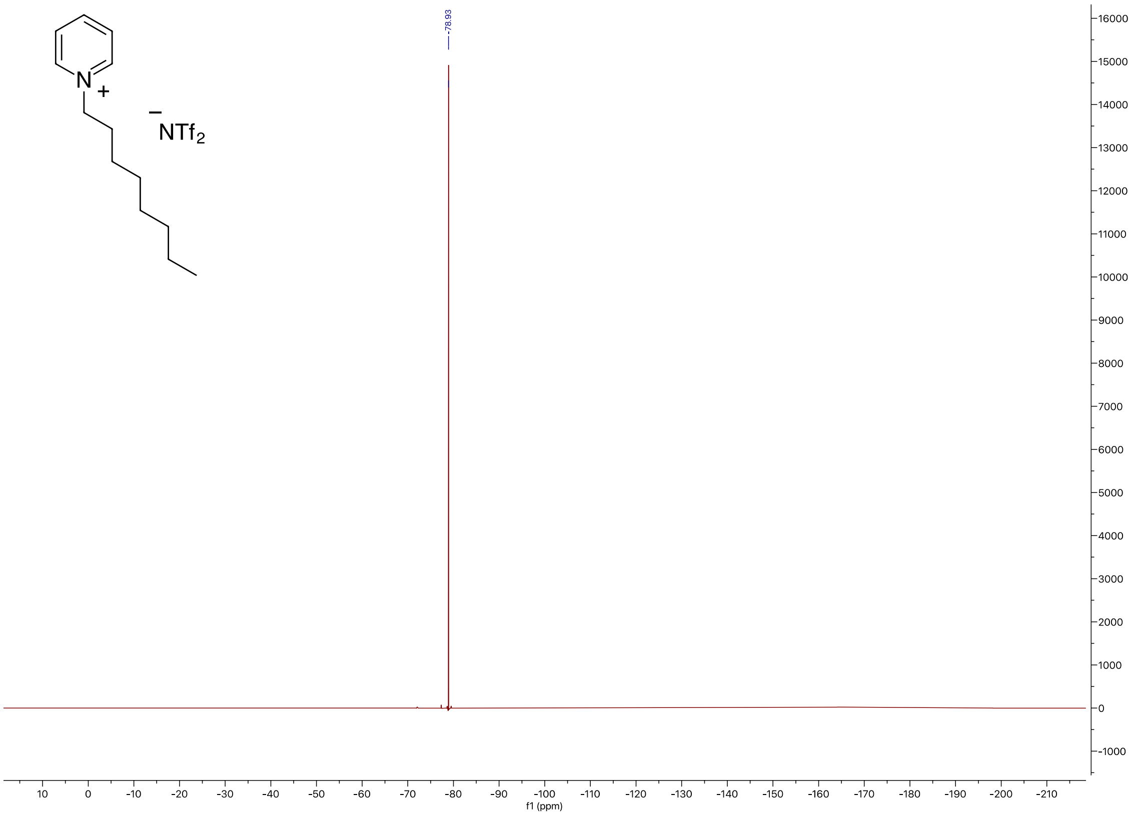

Figure S45. ^{19}F-NMR spectrum of **3c**

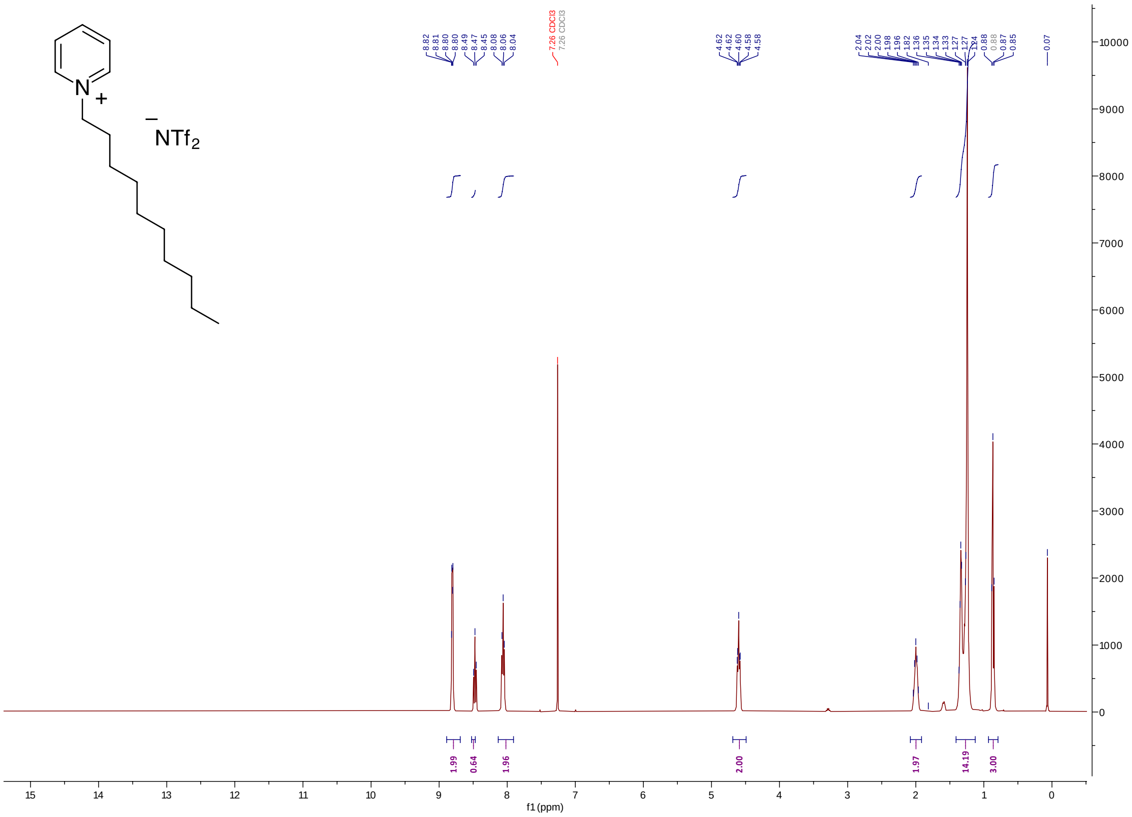

Figure S46. ^{1}H-NMR spectrum of **3d**

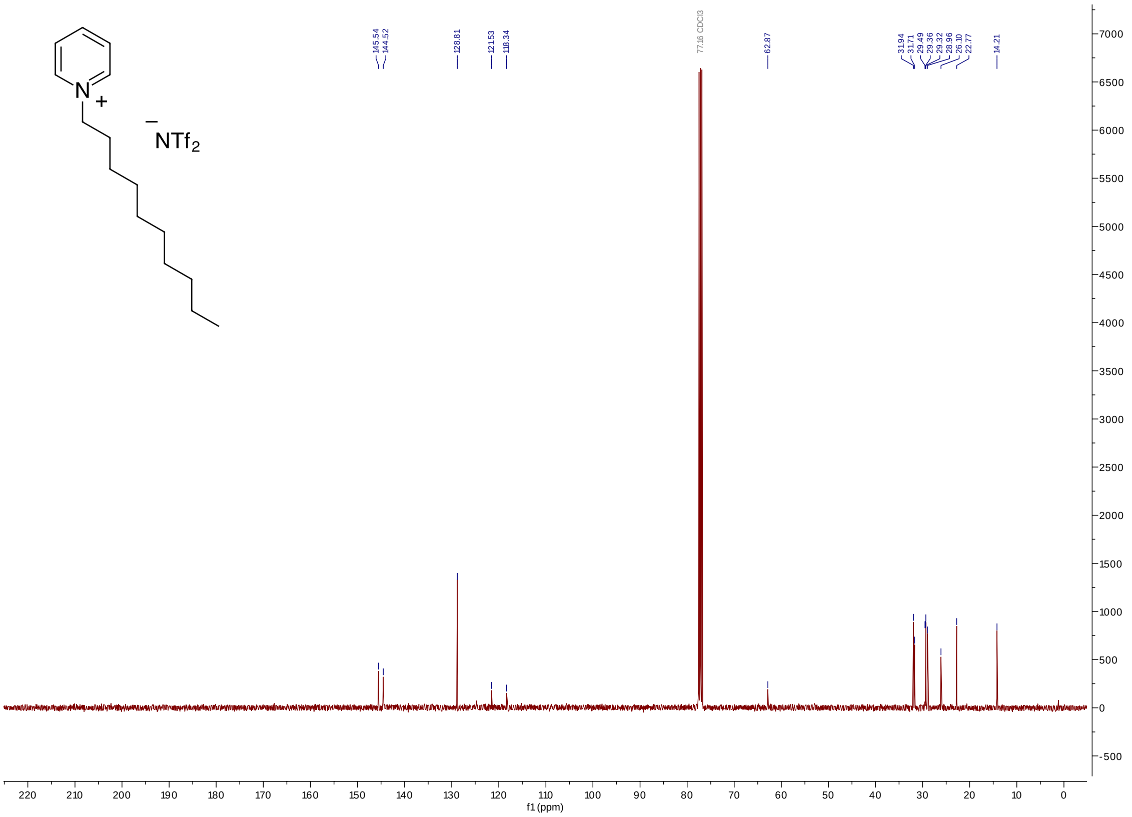

Figure S47. ^{13}C-NMR spectrum of **3d**

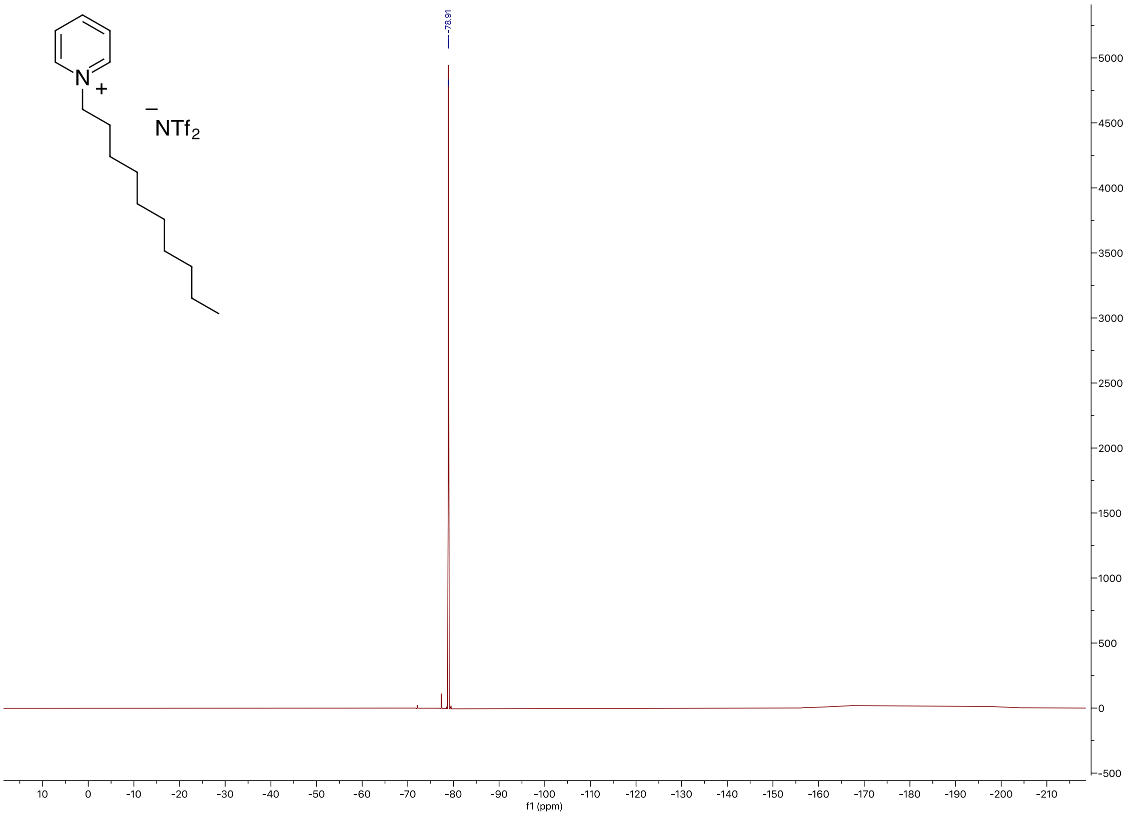

Figure S48. ^{19}F-NMR spectrum of **3d**

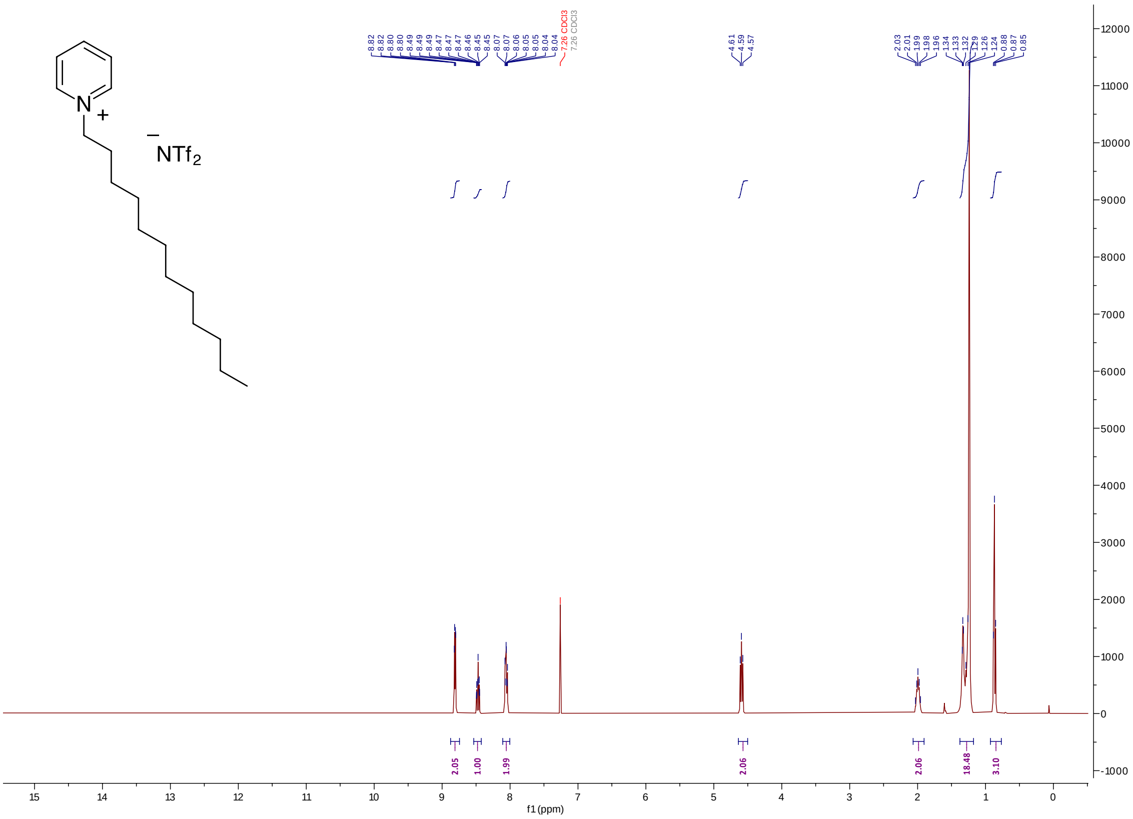

Figure S49. ^{1}H-NMR spectrum of **3e**

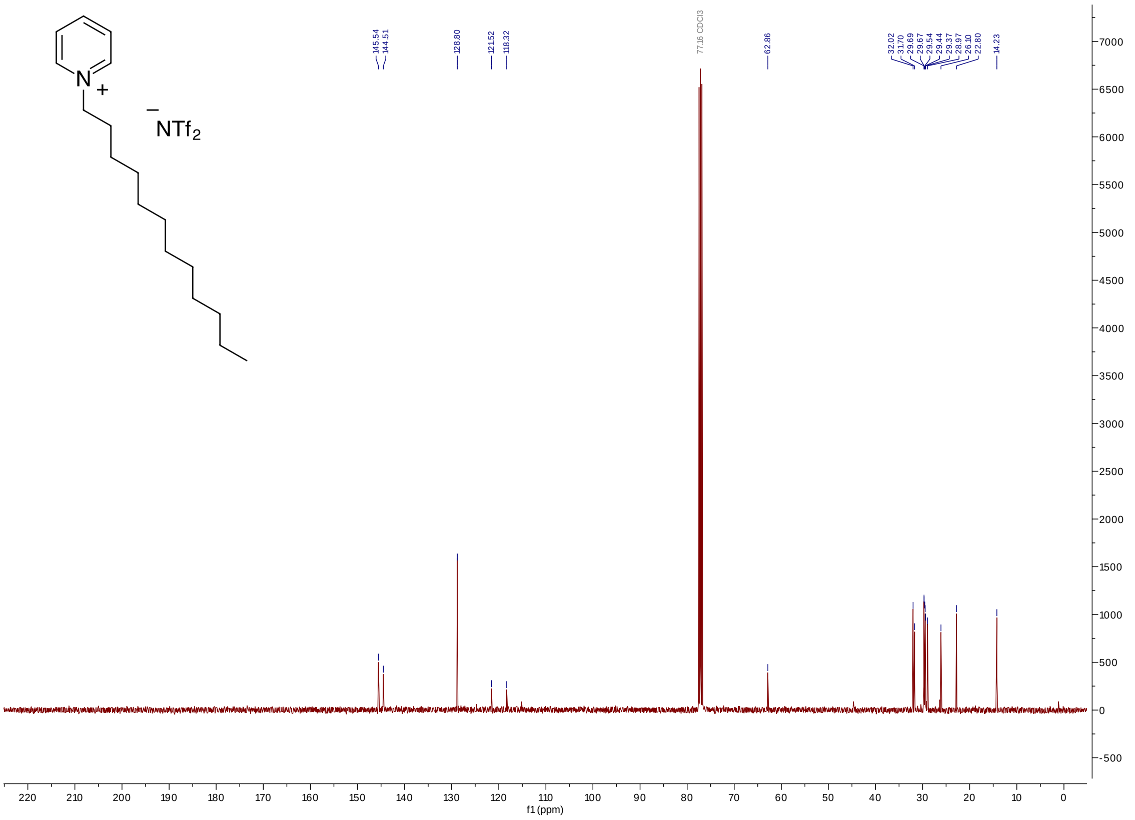

Figure S50. ^{13}C-NMR spectrum of **3e**

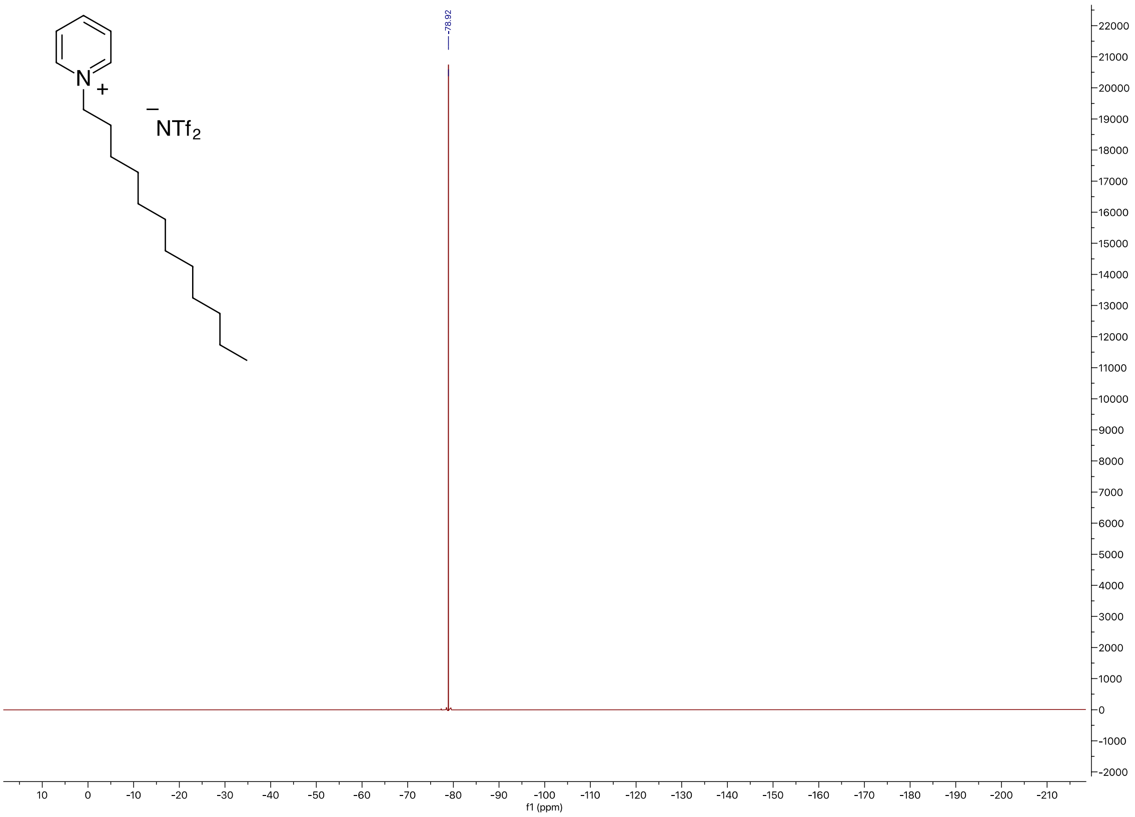

Figure S51. ^{19}F-NMR spectrum of **3e**

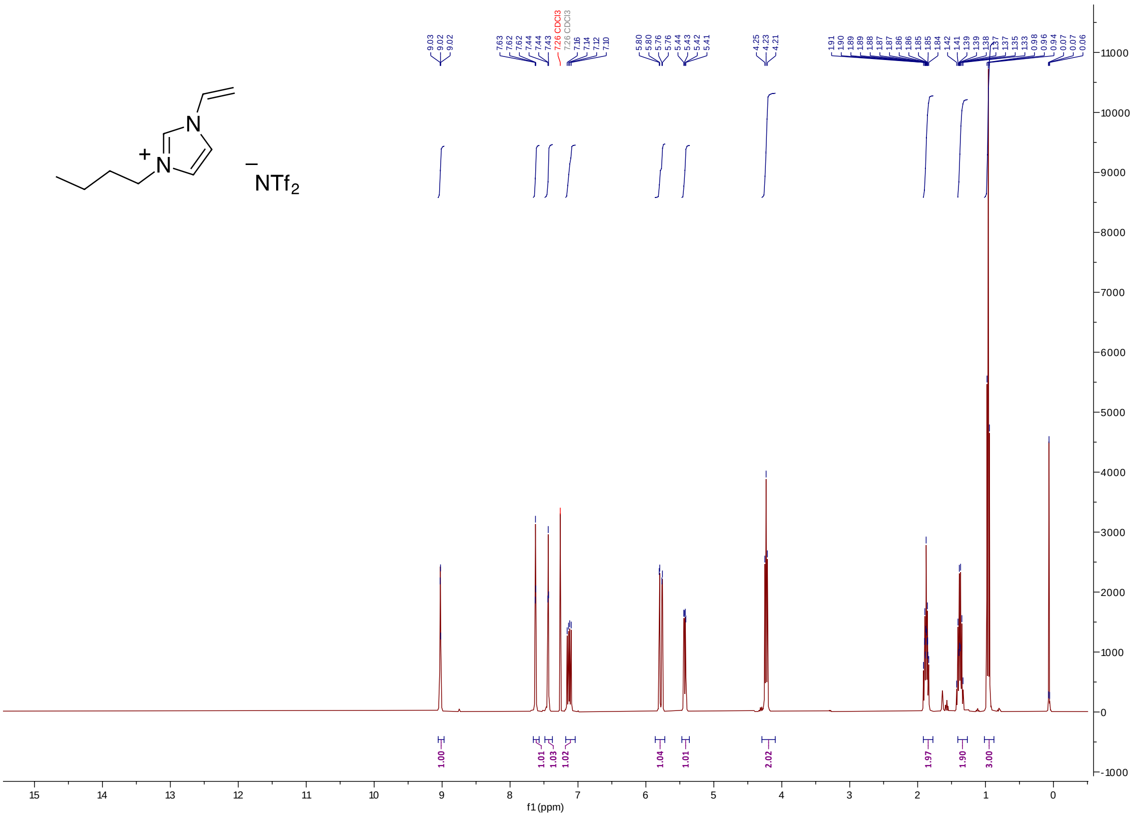

Figure S52. ^{1}H-NMR spectrum of **4a**

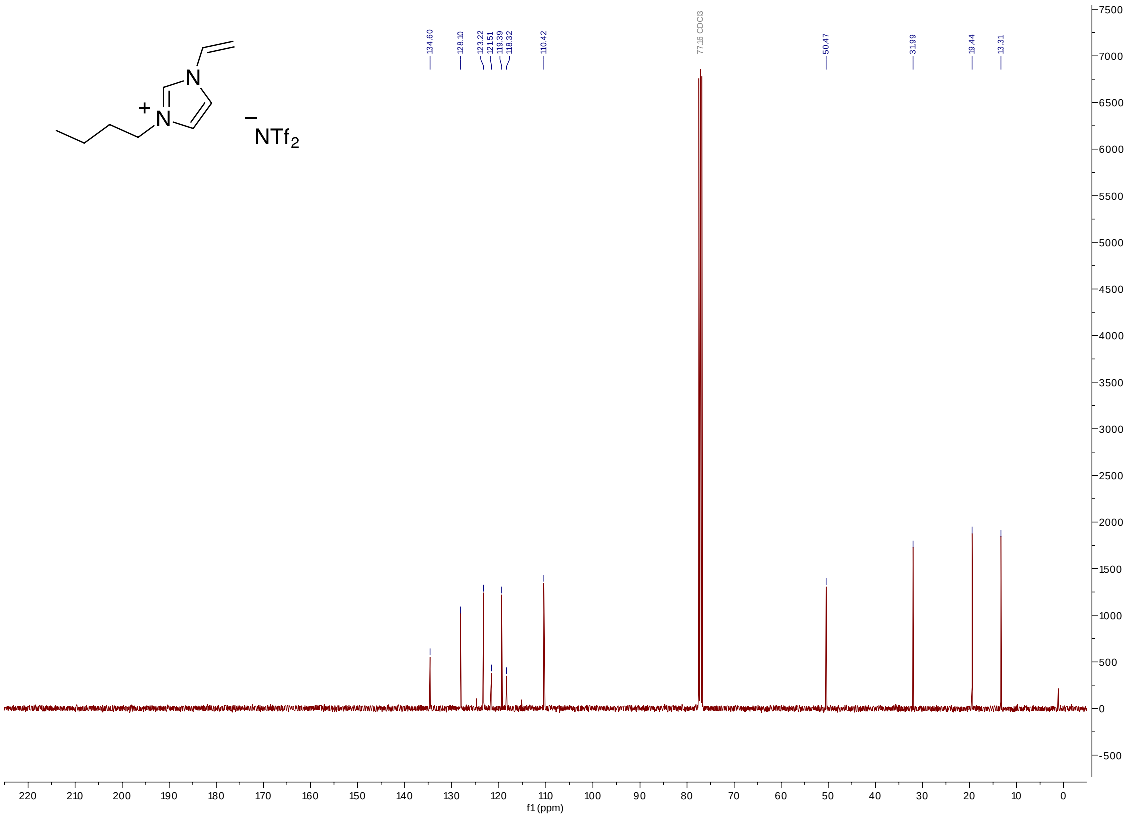

Figure S53. ^{13}C-NMR spectrum of **4a**

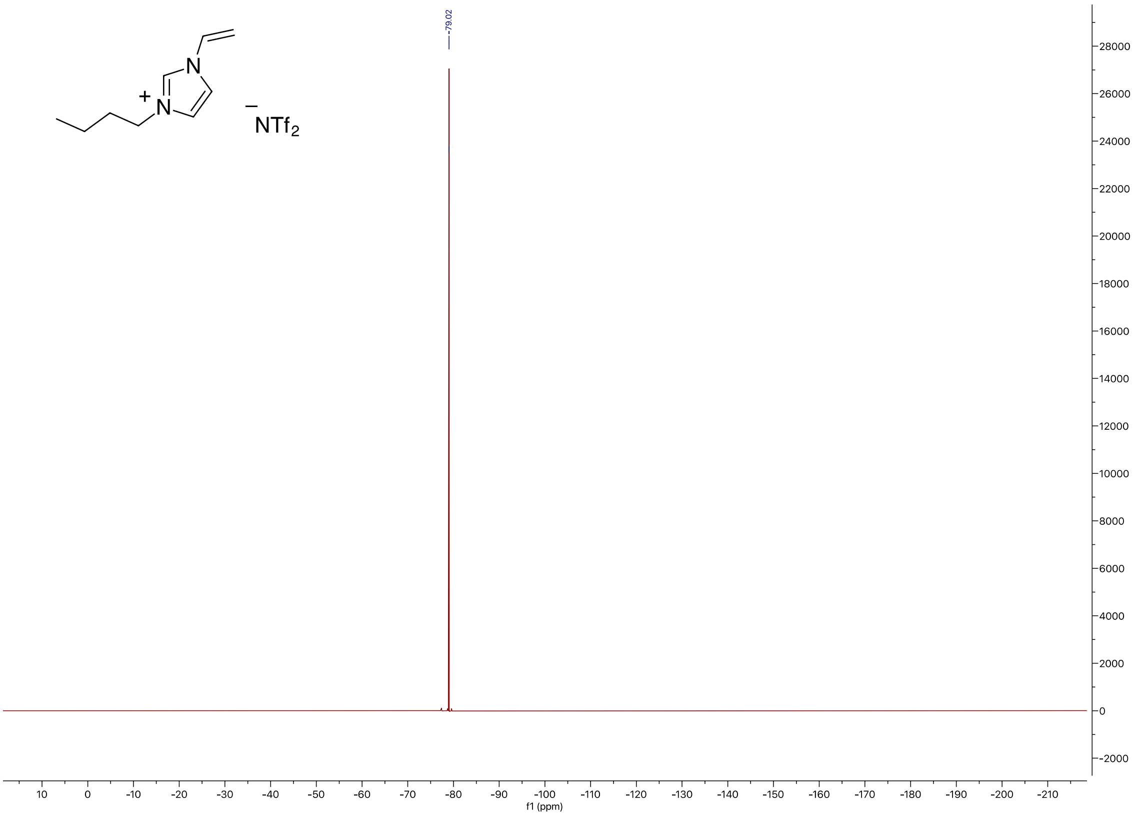

Figure S54. ^{19}F-NMR spectrum of **4a**

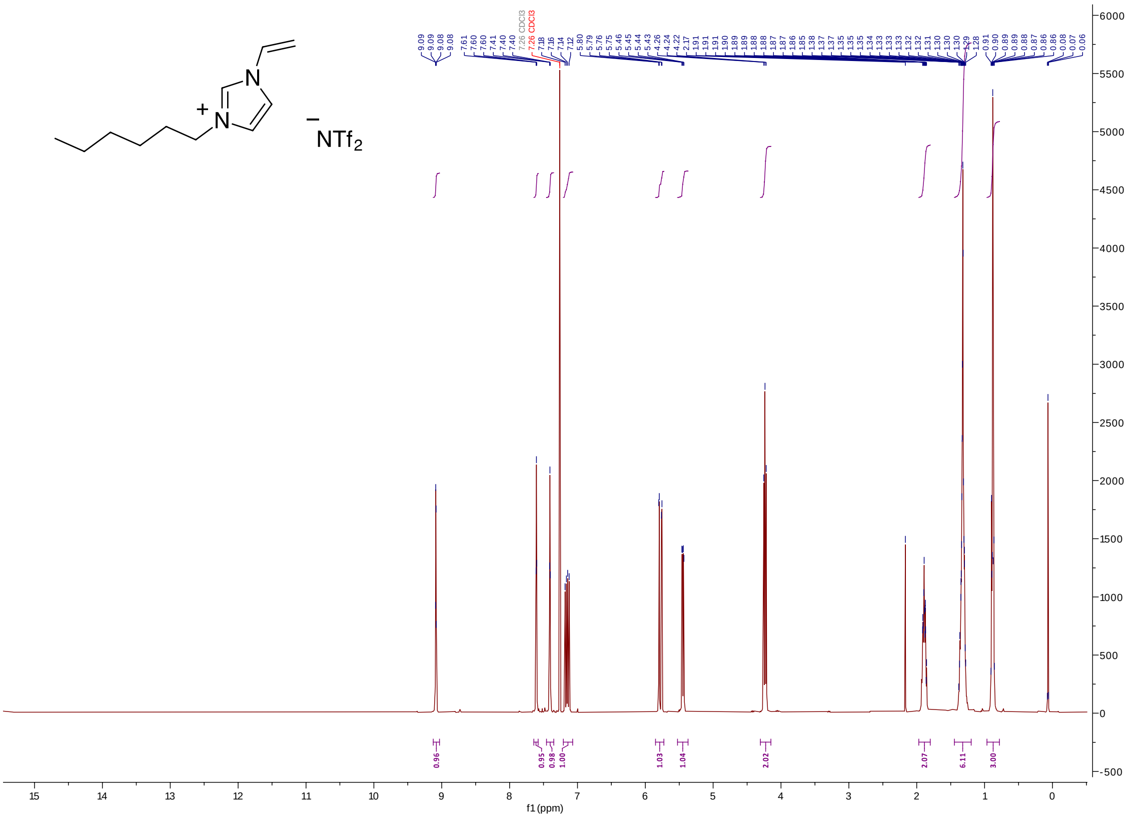

Figure S55. ^{1}H-NMR spectrum of **4b**

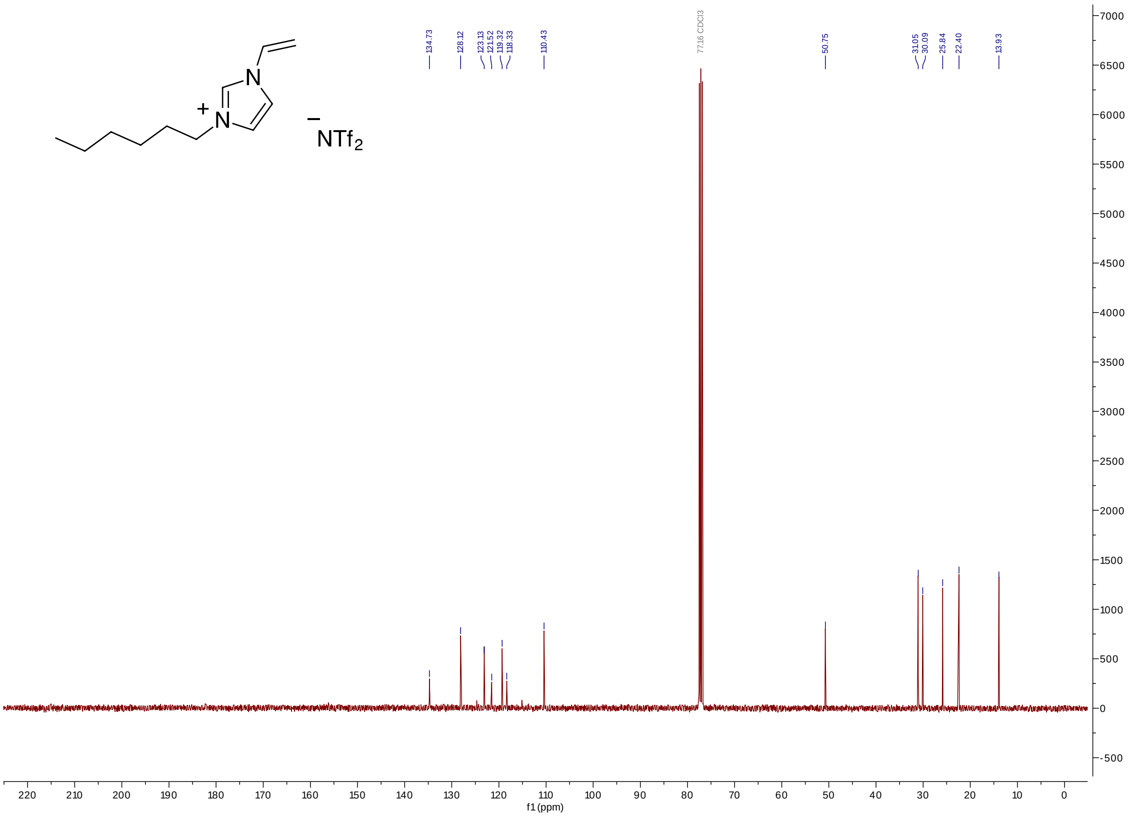

Figure S56. ^{13}C-NMR spectrum of **4b**

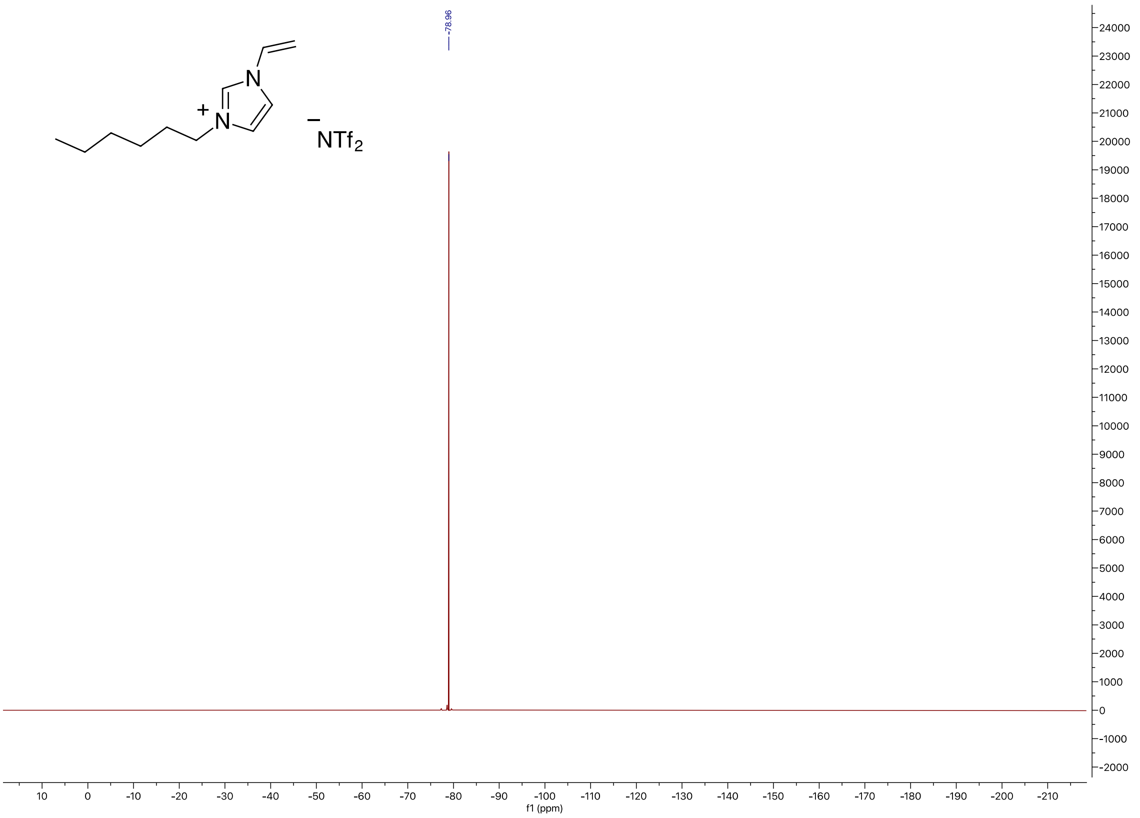

Figure S57. ^{19}F-NMR spectrum of **4b**

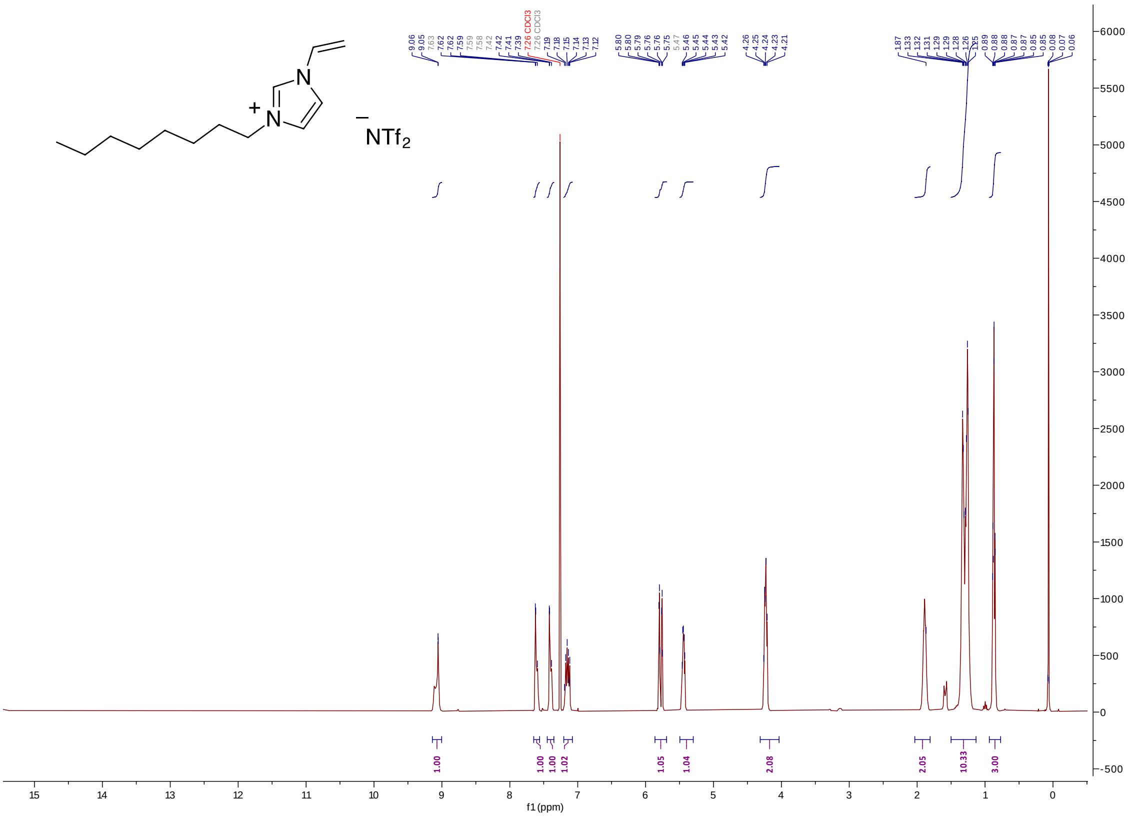

Figure S58. ^{1}H-NMR spectrum of **4c**

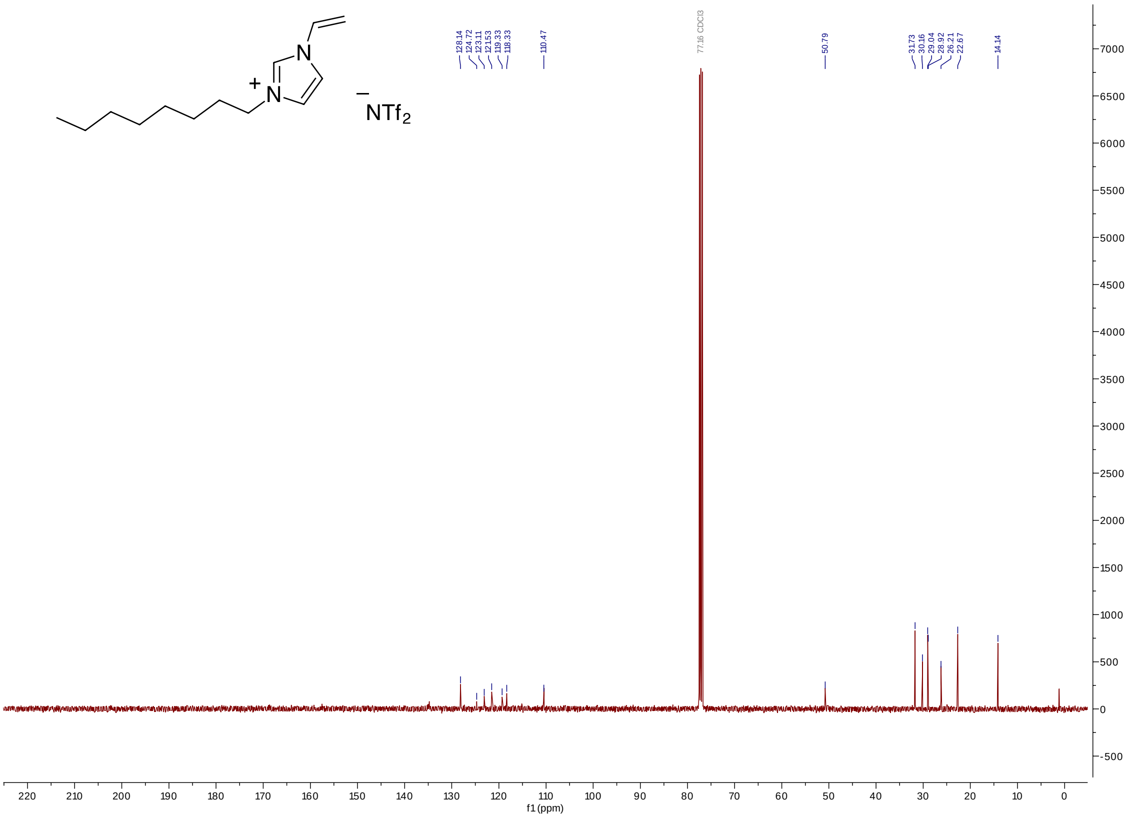

Figure S59. ^{13}C-NMR spectrum of **4c**

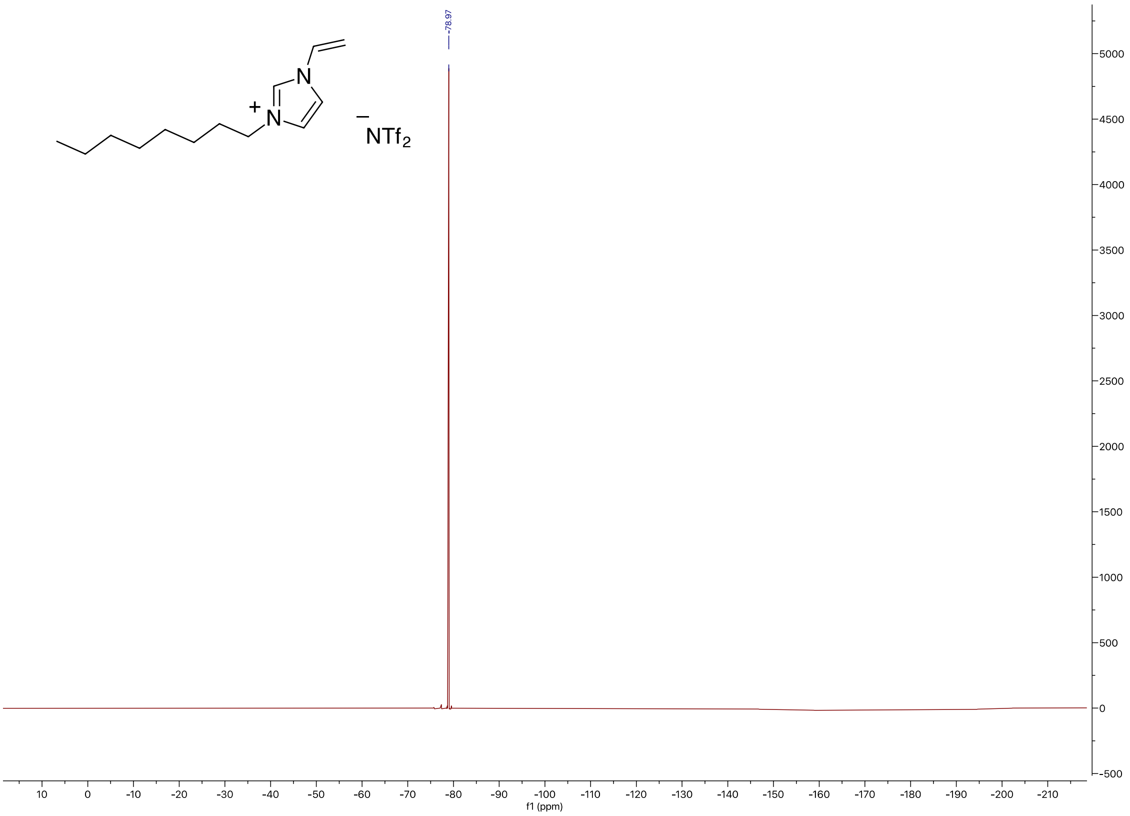

Figure S60. ^{19}F-NMR spectrum of **4c**

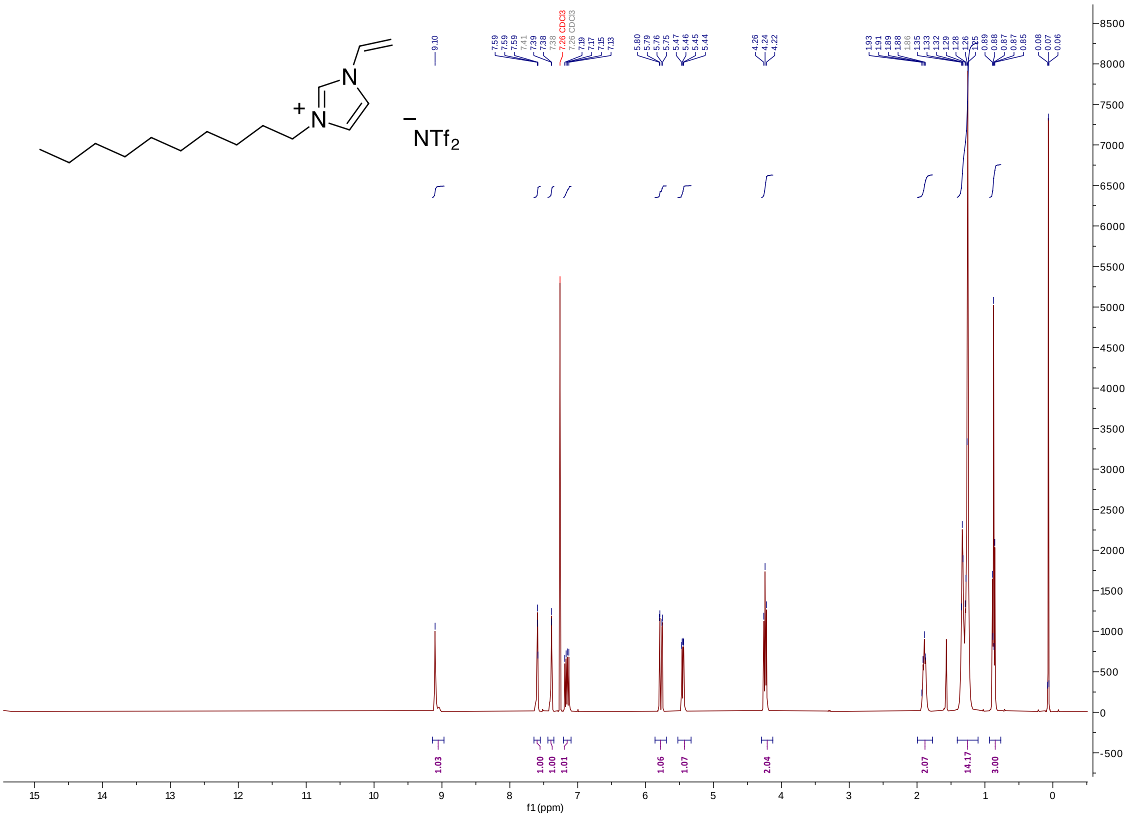

Figure S61. ^{1}H-NMR spectrum of **4d**

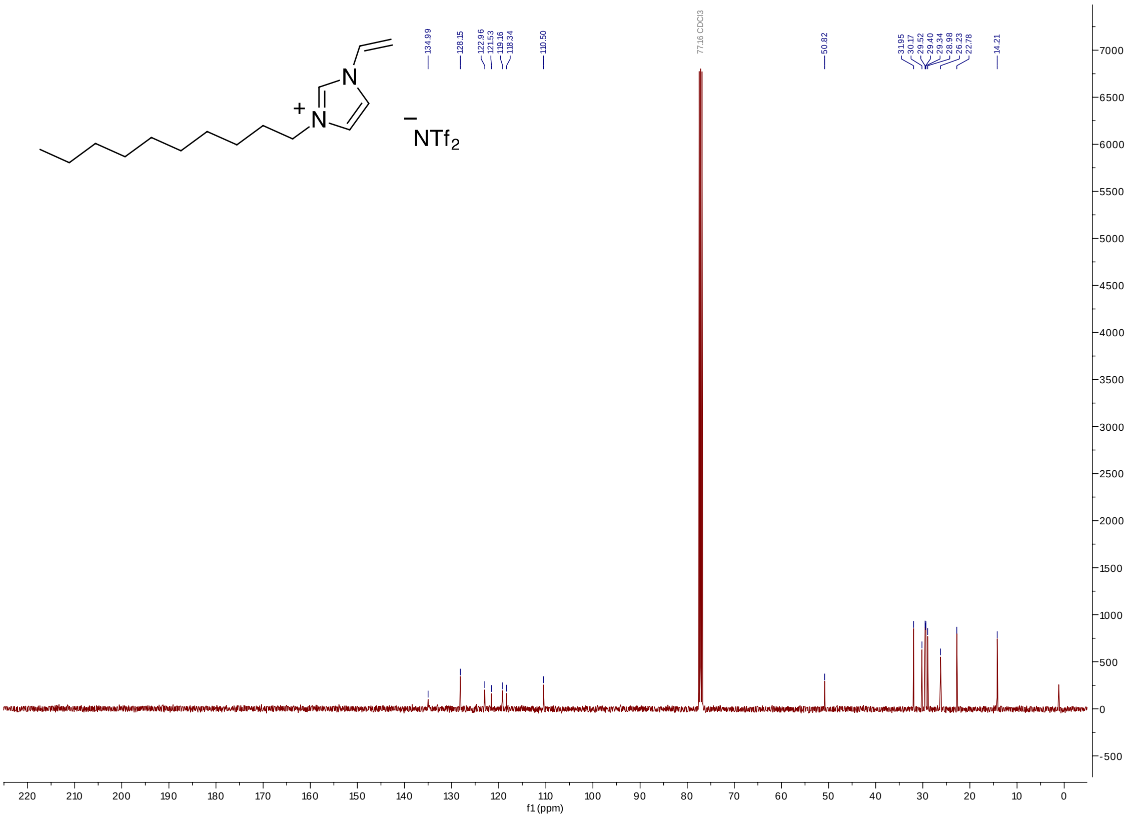

Figure S62. ^{13}C-NMR spectrum of **4d**

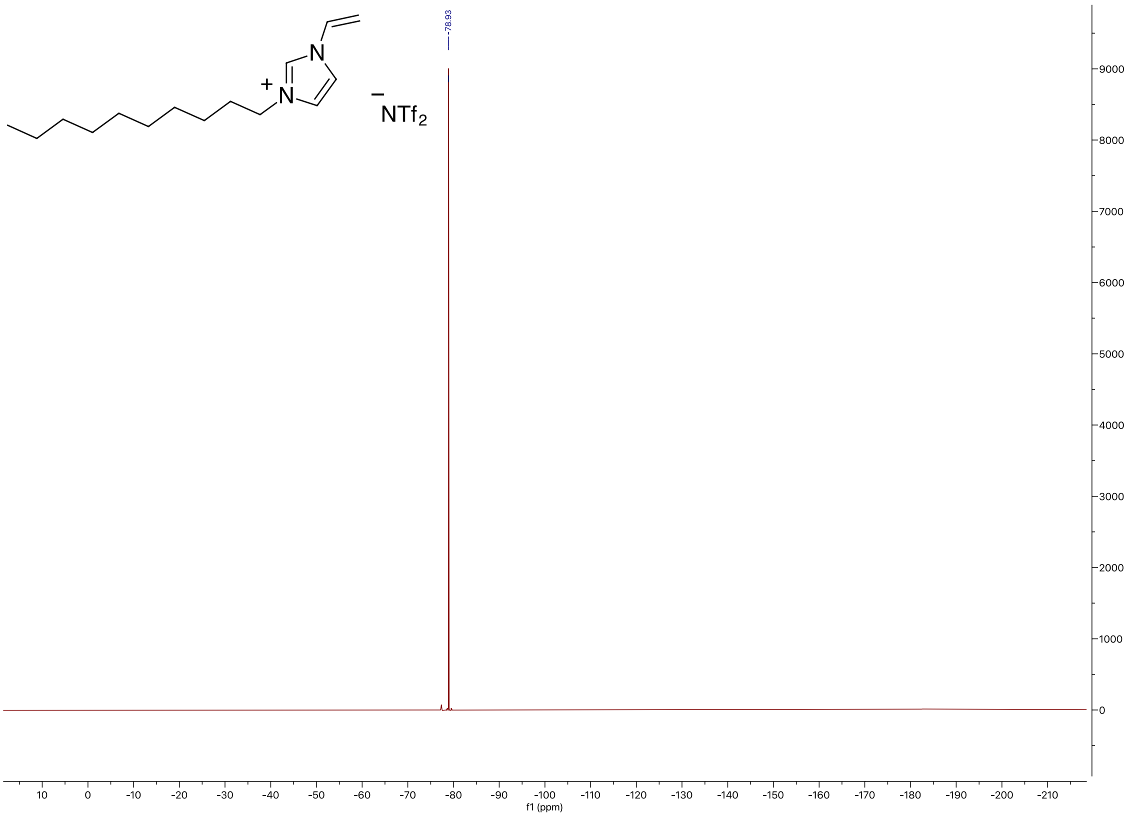

Figure S63. ^{19}F-NMR spectrum of **4d**

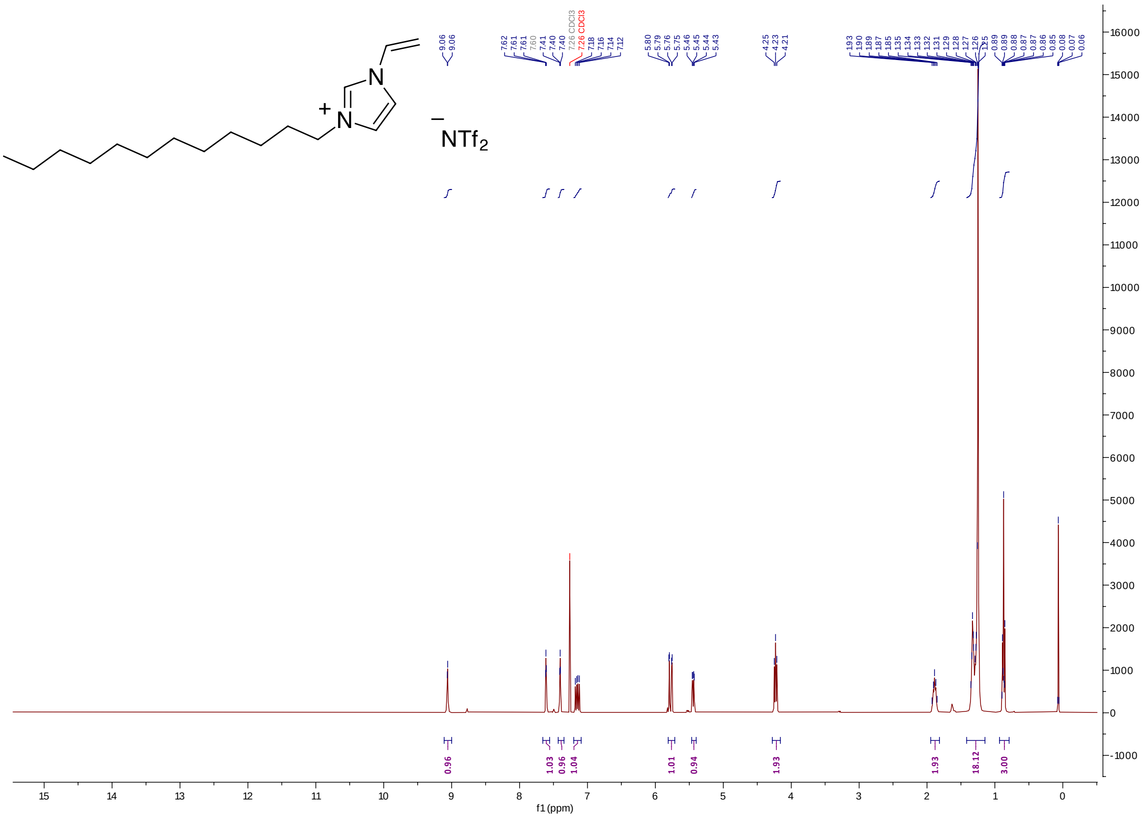

Figure S64. ^{1}H-NMR spectrum of **4e**

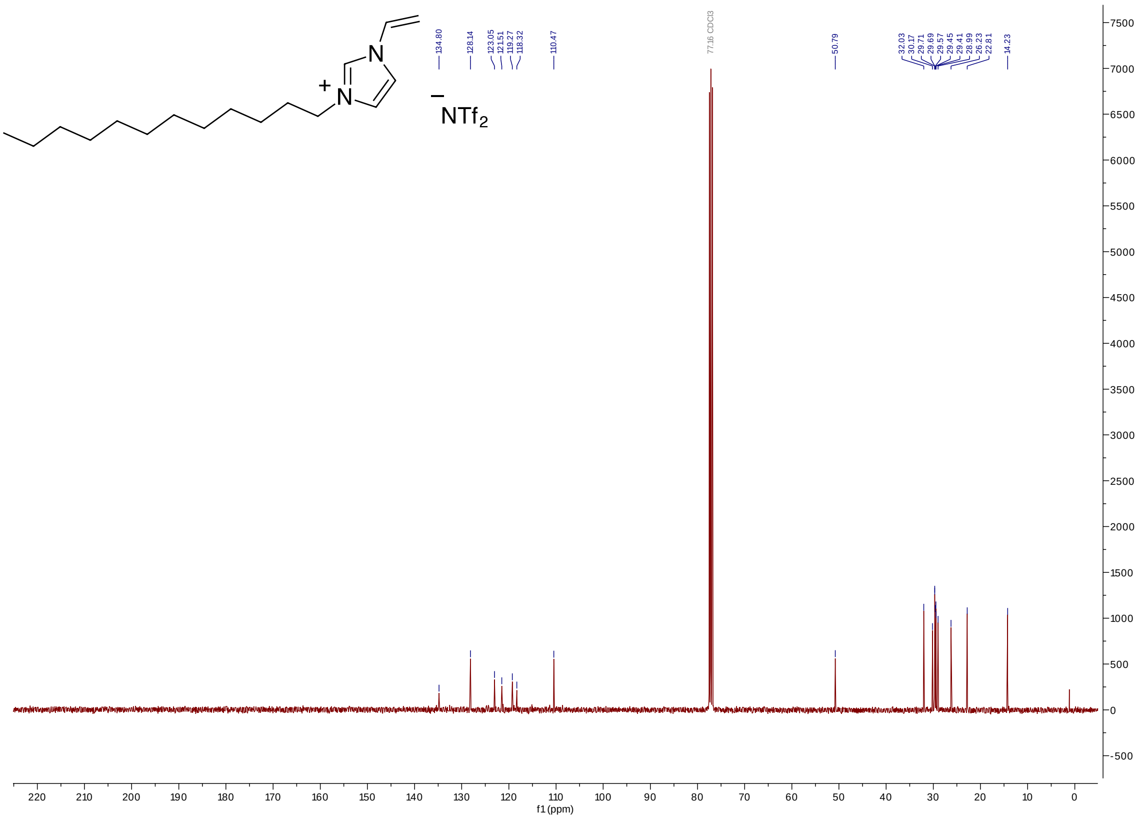

Figure S65. ^{13}C-NMR spectrum of **4e**

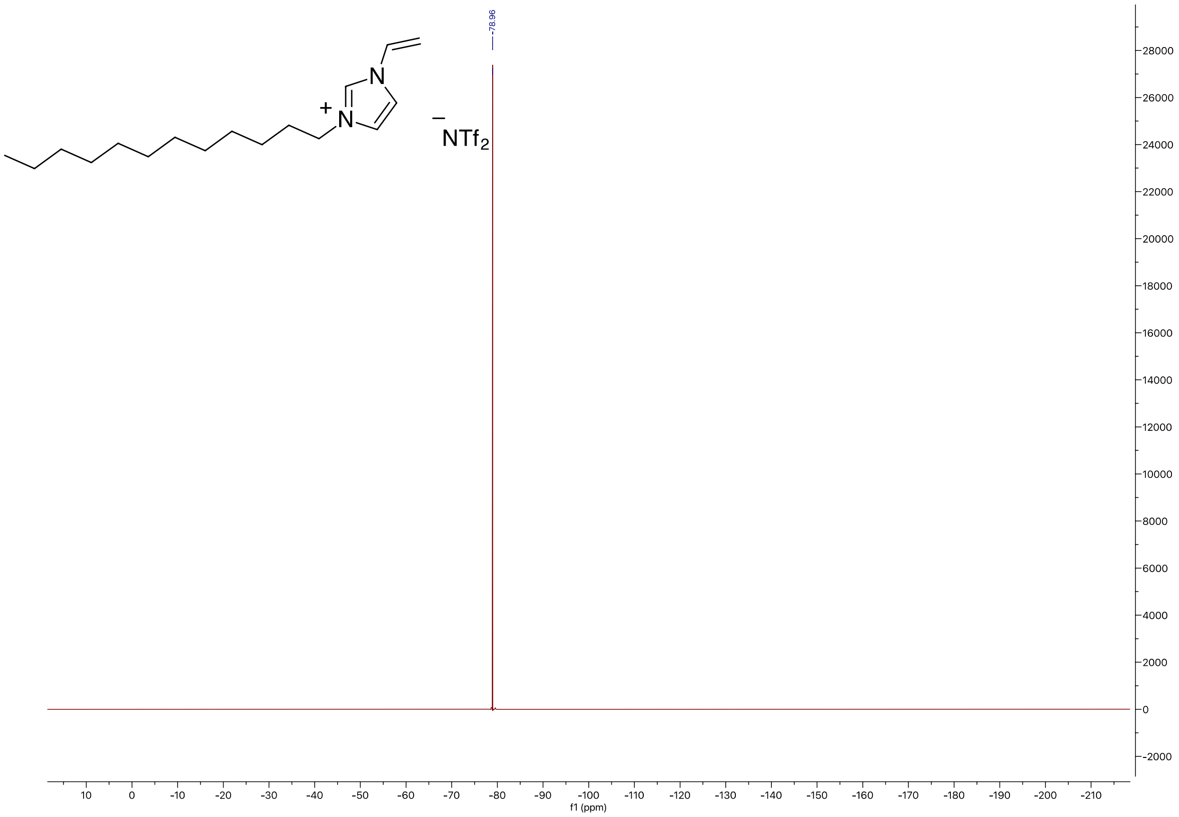

Figure S66. ^{19}F-NMR spectrum of **4e**

14. References

(1) Bica, K.; Schön, M.; Gärtner, P.; Mihovilovic, M. Method for producing ionic liquids. WO 2017/112972 AI, **2017**.

(2) Kawai, R.; Yada, S.; Yoshimura, T. Physicochemical and solution properties of quaternary-ammonium-salt-type amphiphilic trimeric ionic liquids. *Phys. Chem. Chem. Phys.* **2019**, *21*, 25065-25071.
